# Inhaled Corticosteroids Alone and in Combination With Long-Acting β_2_ Receptor Agonists to Treat Reduced Lung Function in Preterm-Born Children: A Randomized Clinical Trial

**DOI:** 10.1001/jamapediatrics.2021.5111

**Published:** 2021-12-13

**Authors:** Nia Goulden, Michael Cousins, Kylie Hart, Alison Jenkins, Gill Willetts, Louise Yendle, Iolo Doull, E. Mark Williams, Zoe Hoare, Sailesh Kotecha

**Affiliations:** 1 NWORTH, Bangor University, Bangor, United Kingdom; 2 Department of Child Health, Cardiff University School of Medicine, Cardiff, United Kingdom; 3 Department of Paediatrics, Cardiff and Vale University Health Board, Cardiff, United Kingdom; 4 Faculty of Life Sciences and Education, University of South Wales, Pontypridd, United Kingdom

## Abstract

**Importance:**

Decreases in future lung function are a hallmark of preterm birth, but studies for management of decreased lung function are limited.

**Objective:**

To determine whether 12 weeks of treatment with inhaled corticosteroids (ICS) alone or in combination with long-acting β_2_ agonists (LABA) improves spirometry and exercise capacity in school-aged preterm-born children who had percent predicted forced expiratory volume in 1 second (%FEV_1_) less than or equal to 85% compared with inhaled placebo treatment.

**Design, Setting, and Participants:**

A double-blind, randomized, placebo-controlled trial was conducted to evaluate ICS and ICS/LABA against placebo. Preterm-born children (age, 7-12 years; gestation ≤34 weeks at birth) who did not have clinically significant congenital, cardiopulmonary, or neurodevelopmental abnormalities underwent spirometry, exercise testing, and measurement of fractional exhaled nitric oxide before and after treatment. A total of 144 preterm-born children at the Children’s Hospital for Wales in Cardiff, UK, were identified and enrolled between July 1, 2017, and August 31, 2019.

**Interventions:**

Each child was randomized to 1 of 3 cohorts: fluticasone propionate, 50 μg, with placebo; fluticasone propionate, 50 μg, with salmeterol, 25 μg; or placebo inhalers, all given as 2 puffs twice daily for 12 weeks. Children receiving preexisting ICS treatment underwent washout prior to randomization to ICS or ICS/LABA.

**Main Outcomes and Measures:**

The primary outcome was between-group differences assessed by adjusted pretreatment and posttreatment differences of %FEV_1_ using analysis of covariance. Intention-to-treat analysis was conducted.

**Results:**

Of 144 preterm-born children who were identified with %FEV_1_ less than or equal to 85%, 53 were randomized. Treatment allocation was 20 children receiving ICS (including 5 with prerandomization ICS), 19 children receiving ICS/LABA (including 4 with prerandomization ICS), and 14 children receiving placebo. The mean (SD) age of children was 10.8 (1.2) years, and 29 of the randomized children (55%) were female. The posttreatment %FEV_1_ was adjusted for sex, gestation, bronchopulmonary dysplasia, intrauterine growth restriction, pretreatment corticosteroid status, treatment group, and pretreatment values. Posttreatment adjusted means for %FEV_1_, using analysis of covariance, were 7.7% (95% CI, −0.27% to 15.72%; *P* = .16) higher in the ICS group and 14.1% (95% CI, 7.3% to 21.0%; *P* = .002) higher in the ICS/LABA group compared with the placebo group. Active treatment decreased the fractional exhaled nitric oxide and improved postexercise bronchodilator response but did not improve exercise capacity. One child developed cough when starting inhaler treatment; no other adverse events reported during the trial could be attributed to the inhaler treatment.

**Conclusions and Relevance:**

The results of this randomized clinical trial suggest that combined ICS/LABA treatment is beneficial for prematurity-associated lung disease in children.

**Trial Registration:**

EudraCT number: 2015-003712-20

## Key Points

**Question:**

Can inhaled corticosteroids (ICS) alone or in combination with long-acting β_2_ agonists (LABA) improve percent predicted forced expiratory volume in 1 second compared with placebo?

**Findings:**

In a randomized clinical trial evaluating 53 preterm-born children, although ICS treatment for 12 weeks improved percent predicted forced expiratory volume in 1 second by 7.7%, the improvement after use of the combination of ICS/LABA was significantly greater at 14.1% compared with the placebo group. Active treatment decreased fractional exhaled nitric oxide and improved postexercise bronchodilator response but did not improve exercise capacity.

**Meaning:**

This trial suggests that combined ICS/LABA treatment is beneficial for prematurity-associated lung disease in preterm-born children.

## Teaser

This randomized clinical trial compares the use of inhaled corticosteroids alone or in combination with long-acting β_2_ agonists in spirometry measures and exercise capacity in preterm-born school-aged children.

## Introduction

Preterm birth, including birth at 33 to 34 weeks’ gestation,^1^ is associated with increased respiratory symptoms^2,3^ and lung function deficits in the longer term, especially in infants who develop bronchopulmonary dysplasia (BPD), also called chronic lung disease of prematurity, in infancy.^4,5^ However, treatment recommendations during childhood and beyond remains largely unknown.^6^ A systematic review on the use of bronchodilators for prematurity-associated lung disease after preterm birth only identified studies evaluating responses after single doses of bronchodilators showing improved forced expiratory volume in 1 second (FEV_1_),^7^ but assessment of longer-term use of bronchodilators is lacking.^8^ Similarly, data on the use of inhaled corticosteroids (ICS) in childhood are limited to 2 studies of preterm-born infants from the presurfactant/perisurfactant era demonstrating no improvement in baseline lung function but improved bronchial lability.^9,10^ The recent European Respiratory Society Task Force on the management of BPD after discharge from the neonatal unit highlighted the substantial lack of evidence on how to treat children who had developed BPD in infancy.^6^

Given the lack of evidence on how to treat the lung disease in individuals with preterm birth, we conducted a double-blind, randomized, placebo-controlled trial to evaluate whether 12 weeks of treatment with ICS alone or in combination with long-acting β_2_ agonists (LABA) improved spirometry measures and exercise capacity in preterm-born children aged 7 to 12 years who had percent-predicted FEV_1_ (%FEV_1_) of less than or equal to 85% compared with inhaled placebo treatment. The primary outcome was between-group differences assessed by pretreatment and posttreatment differences of %FEV_1_, using analysis of covariance (ANCOVA), after 12 weeks of inhaler intervention in the 3 groups. Secondary outcomes included change for other spirometry measures, exercise capacity, and fractional exhaled nitric oxide (FE_NO_) level before and after inhaler intervention.

## Methods

### Participants

The Respiratory Health Outcomes in Neonates (RHiNO) study is a comprehensive study of respiratory disease of preterm-born children evaluating mechanisms, hyperpolarized xenon 129 magnetic resonance imaging, and the current randomized clinical trial.^11^ The protocol and statistical analysis plan of this randomized blinded trial are available in Supplement 1. To identify the participants with %FEV_1_ less than or equal to 85%, we supplemented the responders from a 2013 questionnaire study^3,12^ with additional potential participants identified from the National Welsh Informatics Service and invited them to join the RHiNO study if they were born at 34 weeks’ gestation or less, were aged 7 to 12 years, and did not have substantial congenital abnormalities or severe cardiopulmonary or neurodevelopmental impairment. Data on self-reported race were collected. Recruitment to the trial occurred between July 1, 2017, and August 31, 2019, for children from South Wales who were assessed at the Children’s Hospital for Wales in Cardiff, United Kingdom. Ethical approval was obtained from the South-West Bristol Research Ethics Committee; and parents gave informed written consent and children provided assent. This study followed the Consolidated Standards of Reporting Trials (CONSORT) reporting guideline.

### Outcomes

Two trained research nurses (G.W. and L.Y.) assessed respondents undergoing spirometry (Microloop; CareFusion) before and 15 minutes after bronchodilator administration with 4 puffs of salbutamol (albuterol) (100 μg Salamol [TEVA UK Ltd] administered via a spacer device) and FE_NO_ estimation (NIOX VERO, Circassia Ltd) as previously described^11^ and as detailed in eMethods 1 of Supplement 2. Children who had prebronchodilator %FEV_1_ less than or equal to 85% were invited to the randomized clinical trial including spirometry, exercise capacity testing using cycle ergometry, FE_NO_ assessment, and skin prick testing by a trained physician (M.C.) and advanced nurse practitioner (K.H.) as previously described^13^ before and after 12 weeks of inhaler treatment. The neonatal course was recorded from the neonatal medical notes. Bronchopulmonary dysplasia was diagnosed if supplemental oxygen was required at age 28 days if the infant was born at less than 32 weeks’ gestation or at age 56 days if born at 32 weeks’ gestation or more (mild/moderate/severe BPD according to the National Institute of Child Health and Human Development definitions).^14^

Details on the inhalers, randomization, and masking are given in eMethods 2 of Supplement 2. Briefly, owing to the presence or absence of a counter, a double metered-dose inhaler (MDI) design was used with each child randomized to receive 2 puffs twice daily of placebo/placebo; fluticasone propionate, 50 μg, with placebo; or fluticasone propionate, 50 μg, with salmeterol, 25 μg, inhalers. After extensive discussion, including with 2 independent experts, given the lack of evidence of effectiveness of ICS treatment,^9,10^ children who were using ICS inhalers at the time of the pretreatment visit and who had not had recent respiratory exacerbations, hospital admissions for respiratory reasons, or were not deemed to be ICS dependent were washed out of their corticosteroids under supervision over 4 weeks prior to randomization and then received active treatment (ie, either ICS/placebo or ICS/LABA combination).

The children were monitored for any adverse events during the 12-week treatment period. The trial was overseen by an independent trial and safety monitoring committee.

### Statistical Analyses

Additional detailed description of inhaler intervention, randomization and statistical methods used are given in eMethods2 in Supplement 2.

It was estimated that a third of the children would be receiving preexisting ICS treatment; thus, estimated allocation ratios were 1:1.3:1.3 for placebo:ICS:ICS/LABA. Details of the initial sample size calculation are in eMethods 2 in Supplement 2. During recruitment, the SD of the %FEV_1_ was reviewed. It was also suggested that an improvement of 10% absolute increase in the %FEV_1_ was a more clinically appropriate outcome. A revised power calculation using a conservative SD for %FEV_1_ of 10, α level of .05, and power of 80% suggested that 53 participants with completed data would be required.

ANCOVA was used in accordance with our predefined statistical analysis plan and guidelines provided by the US Food and Drug Administration^15^ and by the European Medicine’s Agency^16^ to assess the pretreatment vs posttreatment differences. Details of imputation used for missing data are given in eMethods 2 in Supplement 2. In the ANCOVA models, the data were adjusted for sex, gestation, BPD, intrauterine growth restriction, pretreatment corticosteroid use status, group, and pretreatment values. Sensitivity analysis was performed for children who were not previously receiving ICS treatment. Assumptions of ANCOVA were tested prior to final model fitting. Repeated-measures analysis of variance was performed to assess change in spirometry measures from baseline to postexercise and from postexercise to postexercise bronchodilator spirometry measures across the 3 treatment groups at pretreatment and posttreatment visits.

All data analyses were performed using Stata, version 15. A *P* value <.05 was considered significant. Adjustments for multiple comparisons were made using the Games-Howell method^11^ for ANCOVA models. All analysis was conducted according to the principle of intention to treat.

## Results

**Figure 1.**
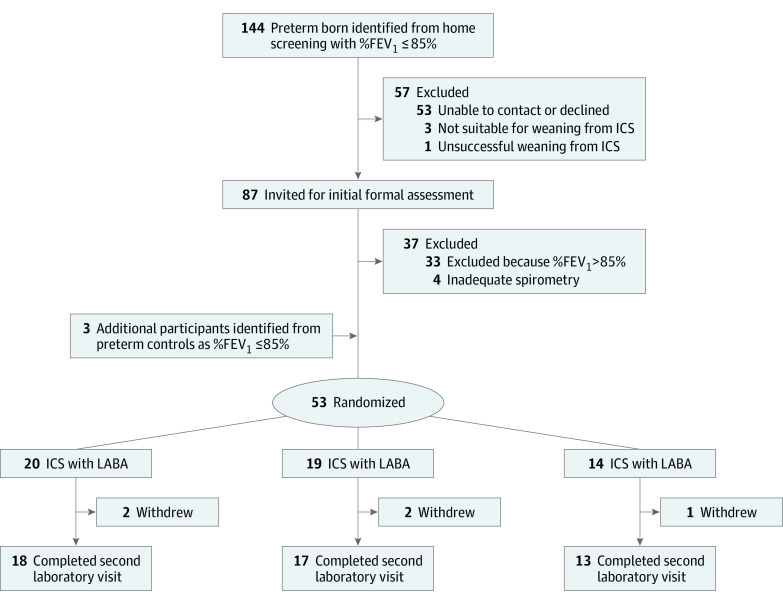
Consolidated Standards of Reporting Trials Flow Diagram %FEV_1_ indicates percent predicted forced expiratory volume in 1 second; ICS, inhaled corticosteroids; and LABA, long-acting β_2_ agonist.

Details of the whole cohort have been described elsewhere.^11^ Of 144 preterm-born children identified with %FEV_1_ less than or equal to 85%, 53 (37%) could not be recontacted or declined participation (Figure 1). From 13 children (9%) receiving ICS treatment, 3 were unsuitable for washout owing to recent admissions or oral corticosteroid treatment, and 1 child developed symptoms during weaning. From the remaining 87 children, including 9 with successful washout from ICS therapy, 4 did not complete satisfactory spirometry tests, and 33 (38%) were excluded because their %FEV_1_ value was greater than 85% at the pretreatment visit. However, 3 children from the preterm control group (who were being studied in parallel) who had %FEV_1_ levels greater than 85% at their screening entered the trial because their %FEV_1_ level was less than or equal to 85% at the pretreatment visit. Thus, 53 were randomized (eTable 1 in Supplement 2). The characteristics of the children who could not be contacted and those who entered the trial were similar (eTable 2 in Supplement 2). Five of the children with washout from ICS treatment were randomized to ICS and 4 were randomized to ICS/LABA. The final allocations were 20 to the ICS, 19 to the ICS/LABA, and 14 to the placebo groups. Five children (ICS, 2; ICS/LABA, 2; and placebo, 1) withdrew, as described in eTable 3 in Supplement 2, including 1 child who developed cough on starting inhaler treatment. There were no other adverse events reported during the trial that could be attributed to the inhaler treatment. However, 2 children (both receiving placebo) developed respiratory exacerbations requiring albuterol and a short course of oral corticosteroids, with 1 of these 2 children also treated with antibiotics; 1 child (ICS/LABA) had a respiratory exacerbation that responded to albuterol.

The mean (SD) age of children was 10.8 (1.2) years, 29 of the children (55%) were female, and 24 (45%) were male; 1 was Asian, 50 were White, and 2 were other race. As reported in Table 1, the participants were well matched. Approximately 40% had BPD in infancy. Respiratory morbidity, including wheeze-ever, recent wheeze, asthma diagnosis, or skin prick results, was similar in all groups.

**Table 1.** Characteristics of the Randomized Children

Characteristic	No. (%)		
	ICS	ICS/LABA	Placebo
No.	20	19	14
Age, mean (SD), y	10.8 (1.3)	10.8 (1.2)	11.0 (1.2)
Sex			
Female	12 (60)	9 (47)	8 (57)
Male	8 (40)	10 (53)	6 (43)
Height, mean (SD), cm	141.4 (10.6)	143.7 (12.2)	145.7 (11.1)
Height, *z* score, mean (SD)	−0.17 (0.9)	0.03 (1.1)	0.21 (1.0)
Weight, mean (SD), kg	36.7 (12.4)	36.5 (9.7)	38.9 (11.3)
Weight, *z* score, mean (SD)	−0.05 (1.1)	−0.06 (0.9)	0.16 (1.0)
BMI, mean (SD)	17.9 (3.9)	17.4 (2.3)	18.0 (3.6)
BMI, *z* score, mean (SD)	0.04 (1.2)	−0.11 (0.7)	0.09 (1.1)
**Perinatal demographic characteristics**			
Gestation, mean (SD), wk	29^+3^ (2)	30^+6^ (2)	29^+5^ (3)
Birth weight, mean (SD), g	1243 (530)	1535 (582)	1412 (592)
Birth weight, *z* score, mean (SD)	0.03 (0.9)	–0.26 (1.0)	0.25 (1.0)
IUGR	4 (20)	6 (32)	2 (14)
Antenatal corticosteroids	19 (95)	16 (84)	13 (93)
Cesarean delivery	11 (55)	12 (63)	6 (43)
Invasive mechanical ventilation	11 (55)	6 (32)	8 (57)
Noninvasive ventilation	6 (30)	6 (32)	3 (21)
Postnatal corticosteroids	2 (10)	0	0
BPD, mild	1 (5)	3 (16)	3 (21)
BPD, moderate/severe	6 (30)	3 (16)	4 (29)
Home oxygen	2 (10)	2 (11)	1 (7)
ROP, IVH, or NEC	6 (30)	5 (26)	5 (36)
PDA	3 (15)	2 (11)	4 (29)
**Family history**			
Antenatal maternal smoking	2 (10)	3 (16)	2 (14)
Current maternal smoking	2 (10)	1 (5)	3 (21)
Family history of asthma^a^	15 (75)	11 (58)	7 (50)
**Respiratory history**			
Bronchiolitis	9 (45)	4 (21)	4 (29)
Positive skin-prick test	6 (30)	3 (16)	5 (36)
Physician-diagnosed asthma	9 (45)	4 (21)	3 (21)
Wheeze, ever	15 (75)	12 (63)	11 (79)
Wheeze, recent^b^	3 (15)	7 (37)	6 (43)
Short-acting bronchodilator use	6 (30)	5 (26)	2 (14)
ICS use	5 (25)	4 (21)	0

Abbreviations: BMI, body mass index (calculated as weight in kilograms divided by height in meters squared); BPD, bronchopulmonary dysplasia; ICS, inhaled corticosteroid; IVH, intraventricular hemorrhage; IUGR, intrauterine growth restriction; LABA, long-acting β_2_ agonist; NEC, necrotizing enterocolitis, PDA, patent ductus arteriosus; ROP, retinopathy of prematurity.

^a^ Physician-diagnosed asthma in parents or siblings.

^b^ Wheeze in past 12 months.

The unadjusted pretreatment and posttreatment spirometry and FE_NO_ measurements are reported in Table 2. The mean (SD) %FEV_1_ for the 53 children entering the trial was 75.3% (9.0%) (range, 53.0%-85.0%). The %FEV_1_ increased from 75.1% to 81.1% (mean difference, 6.0%) and forced midexpiratory flow of 25% to 75% of FVC (%FEF_25%-75%_) increased from 48.1% to 57.6% (mean difference, 9.5%) after ICS treatment. After ICS/LABA treatment, the %FEV_1_ increased from 77.9% to 86.2% (mean difference, 8.3%) and %FEF_25%-75%_ increased from 54.6% to 70.8% (mean difference, 16.2%). The FEV_1_/forced vital capacity (FVC) ratio increased and FE_NO_ levels decreased after both ICS (29.8 to 15.8 ppb) and ICS/LABA (25.2 to 15.9 ppb) treatment, but not in the placebo group (26.4 to 24.2 ppb) with the proportion with FE_NO_ greater than 30 decreasing from 35.0% to 5.6% in the ICS group, and from 26.3% to 5.9% in the ICS/LABA group.

**Table 2.** Pretreatment and Posttreatment Measures

Characteristic	ICS		ICS/LABA		Placebo	
	Pretreatment	Posttreatment	Pretreatment	Posttreatment	Pretreatment	Posttreatment
**Spirometry**						
No.	20	18	19	17	14	13
%FEV_1_, mean (SD)	75.1 (8.0)	81.1 (12.3)	77.9 (7.9)	86.2 (6.4)	72.4 (11.2)	71.2 (12.1)
%FEV_1_ ≤85%, No. (%)	20 (100)	11 (61)	19 (100)	8 (47)	14 (100)	13 (100)
%FEF_25%-75%_, mean (SD)	48.1 (15.2)	57.6 (15.1)	54.6 (19.4)	70.8 (17.0)	48.1 (21.7)	48.2 (23.4)
%FVC, mean (SD)	91.0 (10.5)	91.9 (14.3)	91.8 (8.3)	91.9 (11.0)	90.0 (7.3)	88.8 (12.2)
FEV_1_/FVC, mean (SD)	0.73 (0.10)	0.78 (0.08)	0.75 (0.11)	0.83 (0.08)	0.71 (0.12)	0.71 (0.14)
PEFR, % predicted, mean (SD)	81.3 (13.5)	95.5 (16.3)	81.6 (15.5)	102.0 (14.1)	75.4 (10.8)	79.5 (10.4)
**FE_NO_**						
No.	20	18	19	17	14	13
FE_NO_, mean (SD), ppb	29.8 (31.4)	15.8 (9.8)	25.2 (23.8)	15.9 (14.2)	26.4 (27.4)	24.2 (25.5)
FE_NO_ >30 ppb, No. (%)	7 (35)	1 (6)	5 (26)	1 (6)	4 (29)	4 (31)
**Exercise capacity**						
No.	16	14	18	16	13	11
Peak heart rate, mean (SD), bpm	185.3 (10.7)	181.8 (14.1)	189.3 (14.8)	187.9 (11.2)	189.1 (8.7)	177.8 (17.0)
Peak respiratory rate, mean (SD), bpm	62.0 (10.9)	63.2 (10.1)	65.6 (7.2)	63.7 (10.3)	58.5 (10.6)	60.0 (12.4)
Relative workload, mean (SD), watts/kg	2.2 (0.5)	2.2 (0.5)	2.5 (0.6)	2.5 (0.5)	2.2 (0.5)	2.2 (0.5)
Relative peak O_2_ uptake, mean (SD), mL/kg/min	30.6 (5.8)	31.8 (6.2)	33.2 (6.2)	34.0 (7.2)	31.8 (5.8)	30.7 (5.4)
Relative peak CO_2_ production, mean (SD), mL/kg/min	35.6 (7.6)	36.2 (8.2)	38.7 (8.7)	40.1 (8.2)	37.1 (7.6)	35.2 (8.3)
VE, L (SD)	45.3 (13.5)	50.9 (16.3)	53.0 (16.7)	54.1 (17.9)	51.3 (11.5)	50.6 (14.6)
Relative V̇E, mean (SD), L/kg	1.2 (0.3)	1.4 (0.6)	1.4 (0.4)	1.5 (0.3)	1.3 (0.2)	1.3 (0.3)
VE vs height, mean (SD), L/m	31.6 (7.6)	35.5 (10.9)	36.1 (9.8)	36.7 (9.6)	34.7 (5.6)	33.8 (8.2)
Highest RER, mean (SD)	1.2 (0.1)	1.2 (0.1)	1.2 (0.1)	1.2 (0.1)	1.2 (0.1)	1.2 (0.1)
Breathing reserve maximum, mean (SD), %	17.9 (18.5)	21.5 (20.6)	15.5 (17.0)	20.6 (18.8)	9.1 (15.4)	13.5 (15.6)

Abbreviations: %FEF_25%-75%_, forced midexpiratory flow of 25% to 75% of FVE; FE_NO_, fractional exhaled nitric oxide; %FEV_1_, percent predicted forced expiratory volume in 1 second; FVC, forced vital capacity; ICS, inhaled corticosteroids; LABA, long-acting β_2_ agonist; PEFR, peak expiratory flow rate; RER, respiratory exchange ratio; V̇E, minute ventilation.

The ANCOVA results are presented in eTable 4 in Supplement 2 and the adjusted spirometry data in Table 3. For %FEV_1_, there was a significant interaction between the pretreatment value and the difference between the placebo and ICS/LABA group (*t* = −2.4; *P* = .02). This finding suggests that the gradients of the lines drawn between the pretreatment and posttreatment values for the placebo and ICS/LABA groups are significantly different. Similarly, for the FEV_1_/FVC ratio, there was a significant interaction between the pretreatment values and the difference between the placebo and ICS/LABA group (*t* = −2.55; *P* = .02), as well as the ICS group (*t* = −2.49; *P* = .02), suggesting differences between the placebo group and both the ICS/LABA and the ICS groups. No significant interactions were noted between placebo and active treatment with pretreatment values for %FVC, %FEF_25%-75%_, or peak expiratory flow rate, suggesting that differences between the placebo and active treatment groups were unlikely. The adjusted means for the ICS/LABA group for %FEV_1_ (14.1%; 95% CI, 7.3% to 21.0%; *P* = .002), FEF_25%-75%_ (19.5%; 95% CI, 5.8% to 33.1%; *P* = .03), and peak expiratory flow rate (20.7%; 95% CI, 12.2% to 29.1%; *P* < .001) were significantly higher than those for the placebo group (Table 3). In contrast, the intermediate increases for spirometry measures were not statistically significant between the ICS and placebo groups (%FEV_1_: 7.7%; 95% CI, −0.3% to 15.7%; *P* = .16; FEF_25%-75%_: 7.8%; 95% CI, −5.5% to 21.2%; *P* = .50; and peak expiratory flow rate: 10.8%; 95% CI, 2.1% to 19.4%; *P* = .05).

**Table 3.** Adjusted Mean Spirometry Data From ANCOVA Analysis

Measure	ICS, mean (SD)	ICS/LABA, mean (SD)	Placebo, mean (SD)	ICS – Placebo, mean (95% CI)	*P* value	ICS/LABA – Placebo, mean (95% CI)	*P* value	ICS/LABA - ICS, mean (95% CI)	*P* value
**All randomized children**									
% Predicted FEV_1_	81.6 (12.0)	88.0 (7.1)	73.9 (11.5)	7.7 (–0.3 to 15.7)	.16	14.1 (7.3 to 21.0)	.002	6.4 (0.25 to 12.6)	.12
% Predicted FEF_25%-75%_	59.3 (15.5)	71.0 (16.4)	51.5 (21.9)	7.8 (–5.5 to 21.2)	.50	19.5 (5.8 to 33.1)	.03	11.7 (1.6 to 21.7)	.07
% Predicted FVC	91.0 (13.9)	90.7 (10.8)	90.4 (11.4)	0.6 (–8.0 to 9.1)	.99	0.3 (–7.4 to 8.0)	>.99	–0.3 (–7.5 to 8.1)	>.99
FEV_1_/FVC ratio	0.78 (0.08)	0.82 (0.08)	0.74 (0.13)	0.04 (–0.04 to 0.11)	.59	0.08 (0.01 to 0.16)	.10	0.04 (0.00 to 0.09)	.16
% Predicted PEFR	93.8 (15.5)	103.7 (14.6)	83.0 (10.2)	10.8 (2.1 to 19.4)	.05	20.7 (12.2 to 29.1)	<.001	9.9 (0.5 to 19.4)	.11
**Corticosteroid-naive children**									
% Predicted FEV_1_	79.4 (12.0)	89.6 (8.0)	72.4 (11.5)	7.0 (–0.9 to 15.0)	.26	17.2 (10.2 to 24.2)	<.001	10.2 (3.8 to 16.5)	.03
% Predicted FEF_25%-75%_	56.5 (15.1)	75.9 (15.6)	50.6 (21.9)	5.9 (–7.4 to 19.1)	.69	25.3 (11.9 to 38.8)	.004	19.4 (9.8 to 29.1)	.005
% Predicted FVC	89.6 (14.2)	90.2 (11.0)	88.8 (11.5)	0.8 (–7.9 to 9.5)	.99	1.4 (–6.4 to 9.2)	.94	0.6 (–7.4 to 8.5)	.99
FEV_1_/FVC ratio	0.77 (0.08)	0.84 (0.07)	0.74 (0.13)	0.03 (–0.05 to 0.11)	.74	0.10 (0.03 to 0.18)	.04	0.07 (0.02 to 0.12)	.04
% Predicted PEFR	95.3 (16.7)	100.6 (14.2)	82.4 (10.3)	12.9 (3.9 to 22.0)	.047	18.2 (9.9 to 26.6)	.001	5.3 (–4.5 to 15.0)	.63

Abbreviations: %FEF_25%-75%_, forced midexpiratory flow of 25% to 75%; %FEV_1_, percent predicted forced expiratory volume in 1 second; FVC, forced vital capacity; ICS, inhaled corticosteroids; LABA, long-acting β_2_ agonist; PEFR, peak expiratory flow rate; RER, respiratory exchange ratio.

The results of sensitivity analyses using ANCOVA for the corticosteroid-naive group at randomization were largely unchanged (Table 3; and eTable 5 in Supplement 2) with significant differences again noted for comparisons between the ICS/LABA and placebo groups for %FEV_1_, %FEF_25%-75%_, and peak expiratory flow rate and for the FEV_1_/FVC ratio on this occasion. A significant difference was noted between the ICS and ICS/LABA groups for %FEV_1_, %FEF_25%-75%_, and FEV_1_/FVC ratio for corticosteroid-naive children.

**Figure 2.**
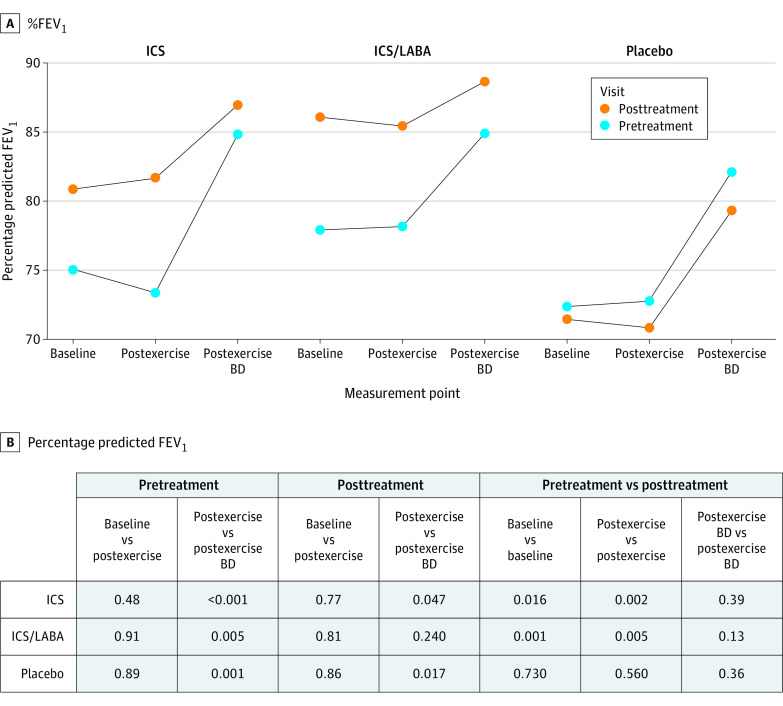
Percent Predicted Forced Expiratory Volume in 1 Second (%FEV_1_) Spirometry Data A, %FEV_1_ at baseline, after exercise, and after postexercise bronchodilator (BD). B, Associated *P* values from comparisons of means of the %FEV_1_. ICS indicates inhaled corticosteroid; LABA, long-acting Β_2_ agonist.

The exercise capacity, including relative workload and relative maximal oxygen uptake, remained unchanged after intervention (Table 2). The pretreatment and posttreatment spirometry data at baseline, after exercise, and after postexercise bronchodilator are reported in eTable 6 in Supplement 2 and Figure 2 for %FEV_1_ and in the eFigure in Supplement 2 for %FVC and FEV_1_/FVC ratio. The baseline %FEV_1_ values improved after intervention with active drugs but not after placebo. There were minimal decreases in %FEV_1_ for each of the 3 groups after exercise in both the pretreatment and posttreatment groups. However, postexercise bronchodilator administration significantly increased %FEV_1_ for all 3 intervention groups at the pretreatment assessment (11.4% [*P* < .001] for ICS, 6.8% [*P* = .005] for ICS/LABA, and 9.5% [*P* = .001] for the placebo groups). After intervention, these significant increases after postexercise bronchodilator administration decreased markedly for both the ICS (4.8%; *P* = .047) and ICS/LABA (2.4%; *P* = .24) treatment groups but continued to improve significantly after placebo treatment (9.9%; *P* = .02).

## Discussion

This trial showed that 12-week treatment with both ICS and combined ICS/LABA improved spirometry in preterm-born children with low lung function compared with placebo treatment. Treatment with ICS/LABA resulted in significant improvements that were additive to ICS treatment alone. For corticosteroid-naive children, there were significant differences between the combined ICS/LABA and placebo groups but also between the ICS and ICS/LABA groups. The FE_NO_ decreased after both active treatments but not after placebo treatment. Neither active treatment affected exercise capacity, but postexercise bronchodilator responses in the ICS and ICS/LABA arms at the pretreatment visit were significantly less at the posttreatment visit.

The European Respiratory Society Task Force report on management of BPD after discharge from the neonatal unit highlighted the lack of evidence on how to treat the respiratory symptoms and decreased lung function noted in childhood and beyond in those who had BPD in the neonatal period.^6^ Data on how to manage lung disease observed in late preterm-born children are also lacking.^17^ Because late preterm-born children, especially those born at 33 to 34 weeks’ gestation, are also at risk of developing lung disease,^1^ we focused on preterm-born children born at 34 weeks’ or less who had low lung function close to the lower limit of normal rather than just focusing on those with BPD especially as a significant proportion of those with BPD have normal lung function in childhood and beyond.

The results showed improvements after ICS treatment which is likely to target any continuing inflammatory processes. Although FE_NO_ has not shown to be increased in prematurity-associated lung disease including those who had BPD in infancy,^18^ the current results show that FE_NO_ decreased from 29.8 to 15.8 ppb in the ICS group, and from 25.2 to 15.9 ppb in the ICS/LABA group. These data suggest that inflammation is likely to play a role. Infants who die of BPD have been shown to have increased airway smooth muscle deposition extending much further distally than in term-born or no-BPD preterm infants.^19,20^ The additional improvements after the addition of LABA is likely to target these structural elements. These observations need further investigation because they suggest the presence of different phenotypes of lung disease after preterm birth.^21^

Previous studies using corticosteroids in children after preterm birth, including those who had BPD in infancy, are limited to 2 studies of children born in the presurfactant/perisurfactant era. Chan and Silverman^9^ studied 15 children in 1993 with low birth weight of (<2500 g), including 10 weighing less than 1500 g at birth, and who had airway response to aerosolized histamine in a 4-week crossover study with beclomethasone dipropionate, 200 μg, twice daily. No improvements from baseline spirometry were noted. In a study from 2001, Pelkonen at al^10^ studied budesonide, 400 μg, twice daily for 4 months in 21 preterm-born children (mean birth weight, 1025 g; range, 640-1600 g; mean gestation at birth, 28 weeks; range, 24-35 weeks), with 18 children completing the study. Baseline spirometry measures did not improve, but bronchial lability improved with the treatment. In our study of a population managed differently from those of the previous studies,^9,10^ we noticed improvements in spirometry with corticosteroids alone; this improvement did not reach the required significance but clearly had some effect given the decrease in FE_NO_.

The addition of the LABA to the ICS resulted in clinically acceptable improvements in spirometry measures. Presumably, the treatment combination targeted both the functional (excessive smooth muscle, as shown previously^19,20^) and any inflammatory processes that may be occurring (as suggested by decreased FE_NO_) in these children with lung dysfunction. It is surprising that the improvements in the 21 studies reported in a systematic review^7^ have not been followed up with longer-term assessments of bronchodilators except the one by Pelkonen et al.^8^ Their 2-week study using terbutaline did not report the posttreatment spirometry levels but noted improved diurnal variation after treatment. In our study design, we had intended for a separate arm of only bronchodilators, but this would have entailed using short-acting β_2_ agonists (given the controversy of using LABA by themselves), which would need to be administered 4 times per day and also entail a complex design of ICS and placebo inhalers also administered 4 times a day; this regimen would have been a great inconvenience to the children who were at school during the treatment period. A study design with twice-daily treatment was deemed more acceptable by several parents interviewed during the study design.

There were no improvements in exercise capacity after treatment with either ICS alone or with the ICS/LABA combination. In another systematic review of exercise capacity,^22^ only an approximate 5% decrease in oxygen uptake (VO_2max_) in those born preterm without BPD and approximate 10% deficits in those who had BPD in infancy compared with term-born control children were noted. Similarly, approximate 10% deficits were noted for physical activity measurements in children who were born preterm compared with term controls.^23,24^ In our present study, the exercise capacity testing may not have been sensitive enough to note small differences, especially if the children had become habitually inactive.^13^ An exercise program taken together with improved spirometry after combined ICS/LABA therapy is very likely to improve physical activity in these children.

It remains to be seen whether the combined treatment has sustained longer term improvements in lung function, exercise capacity, and respiratory symptoms. The goal is to ensure that such treatment is disease modifying, resulting in longer-term improvements of lung function deficits that are increasingly purported to be precursors of development of chronic obstructive pulmonary disease.^25^

The sample size calculation had indicated that it was necessary to recruit 53 children to the trial to achieve 80% power. This was achieved. However, 5 children withdrew from the trial treatment. Using multiple imputation, the analysis was performed on data for 53 children as required; thus, the analysis was adequately powered for detecting a 10% absolute difference in %FEV_1_. It would be beneficial to replicate the findings in other populations to ensure the results are applicable to all groups of preterm-born children with respiratory disease.

### Strengths and Limitations

The main study has, after 2 decades, shown clear benefits of combined ICS/LABA treatment in preterm-born children with lung disease. We had to screen a large number of children to reach an acceptable number to be studied. We deliberately chose a pragmatic value for %FEV_1_ so the results can easily be implemented in the outpatient or general practitioner’s office setting.

The study has limitations. Although our power calculation was modified before and during the study based on the observed SD for %FEV_1_, we would like to have studied larger numbers, with at least 65 participants completing the trial, including a 20% dropout rate, but recruiting was more onerous than we had anticipated given our strict criteria to study only children who would potentially benefit from treatment. In addition, we randomized children who were receiving ICS at the time of randomization. This treatment may have introduced some bias, although this was most acceptable ethically. Results of the analyses of the corticosteroid-naive children were very convincing but had decreased the sample size. Thus, in the future, it would be helpful to replicate the findings in multicenter studies to ensure our results are applicable to all populations of preterm-born children with lung deficits.

## Conclusions

Two decades from the last relevant study, we have shown that combined treatment with ICS and LABA results in significantly improved lung spirometry in contemporary preterm-born children with significant lung function deficits compared with either ICS alone or with placebo treatment.

## Notes

Supplement 1. Trial Protocol and Statistical Analysis Plan

Supplement 2. **eMethods 1.** Physiologic Testing **eMethods 2.** Inhaler Intervention, Randomization, and Statistical Methods **eTable 1.** Randomized Groups **eTable 2.** Comparisons Between Attenders and Nonattenders **eTable 3.** Reasons for Withdrawal **eTable 4.** Results of ANCOVA of Spirometry Measures **eTable 5.** Sensitivity Analyses Showing ANCOVA Results of Spirometry Measures of Children Who Were Corticosteroid Naive at Randomization **eTable 6.** Spirometry at Baseline, Postexercise and After Postexercise Bronchodilator **eFigure.** %FVC and FEV_1_/FVC Ratio at Baseline, After Exercise, and After Postexercise Bronchodilator
eReferences

Supplement 3. Data Sharing Statement
