# Supplementary material for: Inhaled Corticosteroids Alone and in Combination With Long-Acting β2 Receptor Agonists to Treat Reduced Lung Function in Preterm-Born Children: A Randomized Clinical Trial
Source: JAMA Pediatr. 2021 Dec 13;176(2):1–9. doi: 10.1001/jamapediatrics.2021.5111 (PMC8669602; doi:10.1001/jamapediatrics.2021.5111)
Supplement: Supplement 1. — Trial Protocol and Statistical Analysis Plan [file jamapediatr-e215111-s001.pdf]

RHiNO

Respiratory Health Outcomes in Neonates

A randomised, double blind, double-dummy placebo-controlled trial of inhaled treatment to establish the mechanisms of prematurity-associated airway obstruction and inflammation

Version 10

16/10/2018

Identifying Numbers:

Sponsor: SPON1451-15

EudraCT: 2015-003712-20

ISRCTN14767962

General Information

This document describes the trial and provides information about procedures for entering participants into it. The protocol should not be used as an aide-memoir or guide for the treatment of other participants; every care was taken in its drafting, but corrections or amendments may be necessary. Clinical problems relating to this trial should be referred to the relevant Investigator.

Statement of Compliance

This study will adhere to the conditions and principles outlined in the EU Directive 2001/20/EC, EU Directive 2005/28/EC and the ICH Harmonised Tripartite Guideline for Good Clinical Practice (CPMP/ICH/135/95). It will be conducted in compliance with the protocol, the Research Governance Framework for Health and Social Care (Welsh Assembly Government November 2001 and Department of Health 2nd July 2005), the Data Protection Act 1998, and other regulatory requirements as appropriate.

Funding

Funding was awarded from the MRC Experimental Medicine Challenge Grant fund.

Approval signatures

Chief Investigator: .... [signature] .... Date:.22/10/2018......

Name: Sailesh Kotecha

Statistician: ....[signature]........................ Date: 23/10/2018......

Name: Zoë Hoare

Contact Details: Institutions

Sponsor:	Funder:	Clinical Trials Unit/ Coordinating Centre
Cardiff University<br>Research and Innovation Services<br>7th Floor<br>30-36 Newport Road<br>Cardiff<br>CF24 0DE<br>Tel: 029 2087 9131<br>Fax: 029 2087 9280<br>Email: resgov@cardiff.ac.uk	Medical Research Council<br>1 Kemble Street<br>London<br>WC2B 4AN	NWORTH<br>Bangor University<br>Y Wern<br>The Normal Site<br>Holyhead Road<br>Bangor<br>LL57 2PZ<br>Tel: 01248 388095<br>Fax: 01248 382229<br>Email: nworth@bangor.ac.uk

Contact Details: Individuals

Chief Investigator authorised to Sign the Protocol and Protocol Amendments on behalf of the Sponsor:	Lead for Respiratory Physiology	Medical Experts who will Evaluate SAE Reports (In absence of Investigator)
Prof Sailesh Kotecha<br>Department of Child Health<br>Cardiff University<br>University Hospital of Wales<br>Heath Park<br>Cardiff CF14 4XW<br>Tel: 029 2074 4187<br>Fax: 029 2074 4283<br>Email: KotechaS@cardiff.ac.uk	Dr Mark Williams<br>Faculty of Life Sciences and Education<br>University of South Wales<br>Glyn-Taff Campus<br>Pontypridd CF37 4BD<br>Tel: 029 2074 4187<br>Fax: 029 2074 4283<br>Email: mark.williams@southwales.ac.uk	Dr Iolo Doull<br>Children's Hospital of Wales<br>Cardiff<br>CF14 4XW<br>Tel: 029 2074 2159<br>Fax: 029 2074 4283<br>Email: DoullIJ@cardiff.ac.uk
**Head of Trial Management**	**Head of Statistics Team**	**Magnetic Resonance Imaging**
Jean Ryan<br>NWORTH<br>Bangor University<br>Y Wern<br>The Normal Site<br>Holyhead Road<br>Bangor LL57 2PZ<br>Tel: 01248 383238<br>Fax: 01248 382229<br>Email: j.ryan@bangor.ac.uk	Dr Zoe Hoare<br>NWORTH<br>Bangor University<br>Y Wern<br>The Normal Site<br>Holyhead Road<br>Bangor LL57 2PZ<br>Tel: 01248 388840<br>Fax: 01248 382229<br>Email: z.hoare@bangor.ac.uk	Professor Jim Wild<br>Academic Unit of Radiology<br>University of Sheffield<br>Royal Hallamshire Hospital<br>Sheffield S10 2JF<br>Tel: 0114 226 5389<br>Fax: 0114 271 1714<br>Email: j.m.wild@sheffield.ac.uk

Table of Contents

1 **Protocol Summary** 8
2 **Background Information** 10
2.1 Introduction 10
2.2 Aims and Objectives 13
2.3 Potential Benefits and Risks 14
3 **Trial design** 16
3.1 Primary Outcome 16
3.2 Secondary Outcomes 16
4 **Study Population** 17
4.1 Part 1 17
4.1.1 Inclusion 17
4.1.2 Exclusion 17
4.2 Part 2 18
4.2.1 Inclusion 18
4.2.2 Exclusion 18
4.3 Part 3 18
4.3.1 Inclusion 18
4.3.2 Exclusion 18
5 **Assessments and Procedures** 19
5.1 Questionnaire study 19
5.2 Part 1: Screening 20
5.2.1 Screening visit 20
5.3 Part 2 23
5.3.1 Visit 1 23
5.3.2 During the treatment period 28
5.3.3 Visit 2 28
5.4 Part 3 29
5.5 Biological samples 29
6 **Trial Treatment** 31
6.1 Formulation, packaging and supply 31
6.2 Prescription and administration of trial treatment 35
6.3 Storage and stability 35
6.4 Returns and compliance 36
6.5 Unblinding 36
6.5.1 Unblinding of Individual Participants During the Trial 36
6.5.2 At completion of Treatment Phase 37
6.6 Accountability Procedures for Study Treatment/s 37
6.7 Assessment of Compliance with Study Treatment/s 38
6.8 Non-Investigational Medicinal Products 38
6.9 Concomitant medications/Treatments 38
6.10 Co-enrolment Guidelines 39
7 **Statistical Considerations** 41
7.1 Method of Randomisation 41
7.2 Sample size 41
7.2.1 Part 1 41
7.2.2 Part 2 42
7.2.3 Part 3 43

7.3 End of Trial ... 43
7.4 Analysis Plan ... 43
7.5 Interim safety monitoring and analysis ... 44

8 **Pharmacovigilance ... 45**
8.1 Terms and Definitions ... 45
8.2 Reference Safety Information ... 46
8.3 Treatment withdrawal criteria ... 46
8.3.1 Stopping criteria ... 46
8.3.2 Consideration of reproductive potential and administration of IMP ... 46
8.4 Reporting procedures ... 47
8.4.1 Non-Serious AEs ... 47
8.4.2 SAEs ... 48
8.4.3 SUSAR reporting in relation to nIMPs ... 49
8.5 Follow-up After SAEs ... 49
8.6 Nature and frequency of safety assessments ... 49
8.7 Maintenance of Blinding ... 50

9 **Ethical considerations ... 51**
9.1 Approval ... 51
9.2 Considerations ... 51
9.3 Informed consent in the paediatric population ... 51
9.4 Procedure for weaning off steroids ... 53
9.5 Post-treatment management plan ... 54
9.6 Study discontinuation ... 54

10 **Regulatory approval ... 55**

11 **Data collection and Trial Monitoring ... 56**
11.1 Risk Assessment ... 56
11.2 Source data ... 56
11.3 Monitoring ... 56
11.3.1 Direct access to data ... 56
11.3.2 Confidentiality ... 57
11.3.3 Quality Assurance and Quality Control of Data ... 57
11.4 Records Retention ... 58

12 **Indemnity ... 59**

13 **Trial committees ... 60**
13.1 Trial management Group (TMG) ... 60
13.2 Independent Scientific Committee ... 60
13.3 Independent Data and Safety Monitoring Committee (IDSMC) ... 60
13.4 Trial Steering Committee (TSC) ... 61

14 **Publication ... 62**

15 **Protocol amendments ... 63**
15.1 Current version ... 63
15.2 Amendments ... 63

16 **References ... 68**

Abbreviations

ADC	Apparent Diffusion Coefficient
AE	Adverse Event
AR	Adverse Reaction
BPD	Bronchopulmonary Dysplasia
CA	Competent Authority
CISC	Child Information and Assent Form
CLD	Chronic Lung disease of Prematurity
CRF	Case Report Form
FEF	Forced Expiratory Flow
FeNO	Fraction of Exhaled Nitric Oxide
FEV_1	Forced Expiratory Volume in 1 second
FVC	Force Vital Capacity
GP	General Practitioner
IDSMC	Independent Data and Safety Monitoring Committee
ICS	Inhaled Corticosteroids
ISF	Investigator Site File
nIMP	Non-Investigational Medicinal Product
NWORTH	North Wales Organisation for Randomised Trials in Health
PEF	Peak Expiratory Flow
PISC	Parent Information and Consent Form
REC	Research Ethics Committee
RSI	Reference Safety Information
SAE	Serious Adverse Event
SAR	Serious Adverse Reaction
SPC	Summary of Product Characteristics
SUSAR	Suspected Unexpected Serious Adverse Reaction
TMF	Trial Master File
TMG	Trial Management Group
TSC	Trial Steering Committee

1 PROTOCOL SUMMARY

**Title**	RHiNO: Respiratory Health Outcomes in Neonates
**Study design**	Double blind, double-dummy, randomised, placebo controlled
**Phase**	IV
**Population**	Children aged 7-12, born preterm (≤34 weeks)
**Sites**	4
**Duration**	3 years
**Description of intervention**	Fluticasone 50µg/Salmeterol 25µg combination metered dose inhaler, or Fluticasone 50µg, or placebo, two inhalations twice daily for 12 weeks
**Primary objective**	To establish the underlying mechanisms of chronic airway obstruction observed in symptomatic children who are born preterm and to establish if there are different phenotypes of this condition that do or do not respond to standard inhaled therapy.

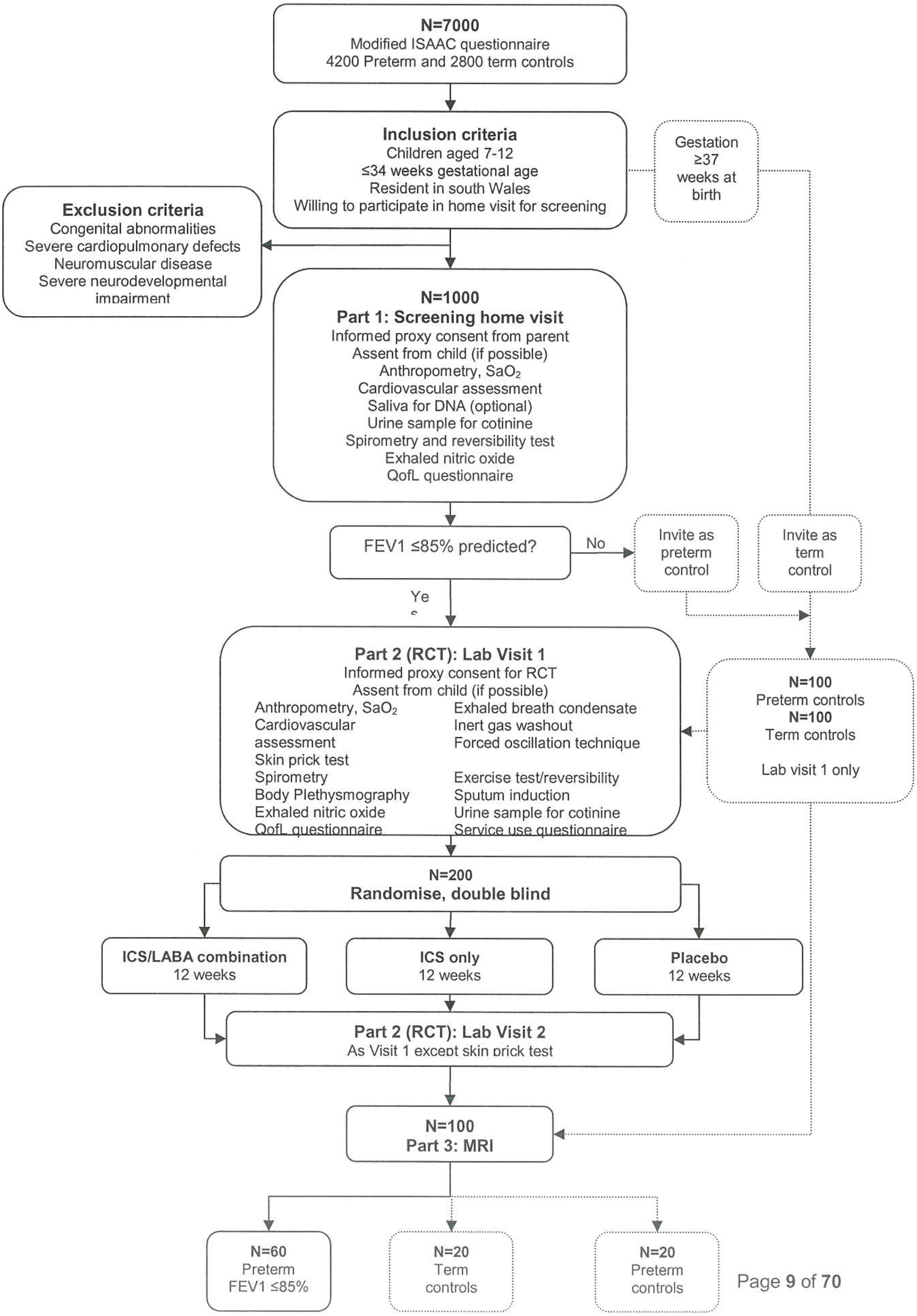
N=7000
Modified ISAAC questionnaire
4200 Preterm and 2800 term controls
Inclusion criteria
Children aged 7-12
≤34 weeks gestational age
Resident in south Wales
Willing to participate in home visit for screening
Gestation ≥37 weeks at birth
Exclusion criteria
Congenital abnormalities
Severe cardiopulmonary defects
Neuromuscular disease
Severe neurodevelopmental impairment
N=1000
Part 1: Screening home visit
Informed proxy consent from parent
Assent from child (if possible)
Anthropometry, SaO_2
Cardiovascular assessment
Saliva for DNA (optional)
Urine sample for cotinine
Spirometry and reversibility test
Exhaled nitric oxide
QofL questionnaire
FEV1 ≤85% predicted?
No
Yes
Invite as preterm control
Invite as term control
Part 2 (RCT): Lab Visit 1
Informed proxy consent for RCT
Assent from child (if possible)
Anthropometry, SaO_2
Cardiovascular assessment
Skin prick test
Spirometry
Body Plethysmography
Exhaled nitric oxide
QofL questionnaire
Exhaled breath condensate
Inert gas washout
Forced oscillation technique
Exercise test/reversibility
Sputum induction
Urine sample for cotinine
Service use questionnaire
N=100
Preterm controls
N=100
Term controls
Lab visit 1 only
N=200
Randomise, double blind
ICS/LABA combination
12 weeks
ICS only
12 weeks
Placebo
12 weeks
Part 2 (RCT): Lab Visit 2
As Visit 1 except skin prick test
N=100
Part 3: MRI
N=60
Preterm
FEV1 ≤85%
N=20
Term controls
N=20
Preterm controls

2 BACKGROUND INFORMATION

2.1 Introduction

Whilst survival of extremely preterm born babies (of 32 weeks or less in gestation) has markedly improved, it is increasingly recognised that these babies may have adverse longer term respiratory outcomes and are indeed now considered at risk of developing COPD in the future (Bolton et al.,2012). Although repeated studies report deficits for FEV_1, there is limited evidence of whether the airway obstruction is reversible and if any treatment has longer term benefits. Pelkonen et al have shown in the 1990's that school age children born preterm commonly have bronchial lability and increased bronchial responsiveness (Pelkonen et al., 1997). More recently, the EPICure study of children born at 26 weeks or less in gestation reported that half the children who had been asymptomatic of respiratory illness over the previous 12 months had reduced lung function (Fawke et al., 2010). Furthermore, a third of the children had reversible airway disease (>12% change in FEV_1) but only around 10% were receiving bronchodilators. Our own data has shown that children who had CLD in infancy, commonly had reversible exercise induced broncho-constriction (Figure 1) (Joshi et al., 2012) but few children were receiving any treatment for their reversible airway disease. Medications such as inhaled long-acting $ß_2$ receptor agonists and corticosteroid are licenced to treat symptoms such as reduced FEV_1 and bronchoconstriction on exercise but their efficacy in unproven in the preterm population.

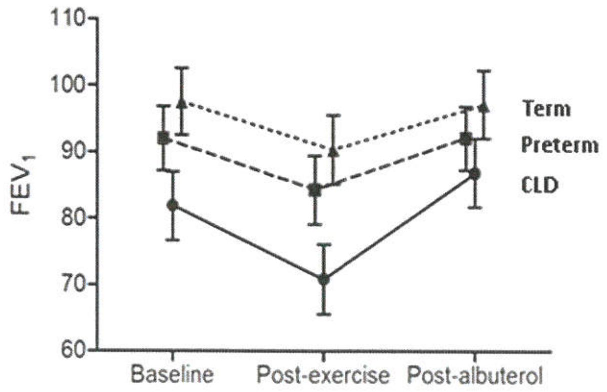

Figure 1: pre-and-post exercise FEV1 and reversibility using FEV1 in preterm children (from Joshi et al., 2012)

Rates of preterm birth have increased in many countries. In Wales, from 35,849 registrable births in 2011, approximately 7 – 8% were born preterm (<37 weeks of gestation) (Kotecha S et al., 2012; ONS, 2012) whilst in the USA the rates of preterm birth have increased up to 12% (Goldenberg et al., 2010). The vast majority of these preterm born infants are born late preterm, i.e. between 33 and 37 weeks gestation, and have been assumed to have normal longer term respiratory outlook but increasingly this proportionately larger group of infants are recognised as having greater respiratory morbidity than previously appreciated (Kotecha SJ, et al., 2012a). In these infants, rates of respiratory disease including respiratory distress syndrome, transient tachypnoea and infections are greater in late preterm infants than term born infants (The Consortium on Safe Labour, 2010). Data beyond this period are very limited but we have reported the only study of longer term respiratory outcomes of children who were born moderately preterm, at between 33 and 34 weeks gestation and were largely not mechanically ventilated at birth, showing deficits of FEV_1 in childhood (Kotecha SJ et al., 2012b). At eight years the decrements in FEV_1 were similar to those born at ≤32 weeks gestation but showed some improvements after undergoing puberty. Most children were not on regular bronchodilator treatment. Children who are born preterm may have wheezing and are often labelled as suffering from asthma but the rates of asthma and atopy between our preterm groups were similar or only marginally different (Kotecha SJ et al., 2012b). Clearly the underlying mechanisms are likely to be related to the developmental abnormalities (Joshi S & Kotecha S, 2007) resulting from preterm birth and from interventions such as mechanical ventilation and oxygen therapy thus need further investigation (Chakraborty M, et al. 2010).

The incidence of preterm birth is increasing, as is overall birth rate. More children born extremely preterm (≤28 week) are likely to survive, and are treated differently due to advances in care. They most likely had RDS and probably CLD in the neonatal period, and form a new population because they are born at different stage in lung development.

Concurrently there are also the group of infants born very preterm (29-34 weeks gestation) comparatively treated as though term, however they were also born at an important time in lung development and less is known about how they progress. Those of 35-36 weeks gestation will not be included in our studies.

It has been show in large cohort studies (ALSPAC and EPICure) that children born preterm have decrements in lung function at school age, children with a history of Bronchopulmonary Dysplasia/Chronic Lung Disease of Prematurity (BPD/CLD) are worse still. Some children suffer from mild respiratory symptoms but are generally well. Preterm children appear to have similar exercise capacity to term children, but report less exercise albeit with use of greater ventilatory reserve. On objective monitoring however they seem to be equally active. This may be so because very few children participate in recommended levels of activity (Lowe J, 2015).

The mechanisms for reduced lung function are not clear, and long term outcomes are not known because the post-surfactant population is still young. Currently they are not under any extra surveillance by healthcare professionals but the burden may start to be recognised. From ALSPAC we envisage that approximately 20% of preterms will have FEV_1 under 85% predicted. Possible causes of this could be a combination of processes with smooth muscle, atopy and inflammation all playing a role. These participants may respond to treatment with low dose combination therapy. Others may not be responsive, and perhaps related to structural aspects. There is growing interest in identifying the phenotype of these participants and how this relates to development of asthma and/or respiratory disease in adulthood.

Children born extremely preterm have been reported to have increased symptoms in childhood but it is unclear if the lung spirometry deficits we have reported for late preterm born children are translated into respiratory symptoms. In order to address this issue, we have recently mailed 26,722 questionnaires (including 13,361 to preterms & 13,361 to terms; 12,812 to 5 - 9 year old children & 13,910 to <5 years of age) to all preterm-born children in Wales who are now aged 1, 2, 3, 5, 7 and 9 years together with matched controls to assess their respiratory symptoms, atopy, hospital admissions and drug usage, etc. using a standardised validated respiratory questionnaire (Asher MI, et al.).

Late preterm born children have thus far been largely ignored and dismissed as being no different to term born children but increasing evidence suggests that this large population of children do have limitations of lung function which may be responsive to bronchodilation. Furthermore, extremely preterm born infants, who are well known to have longer term FEV_1 decrements, have not been systematically investigated for

improvements of symptoms or lung spirometry after bronchodilator treatment. This study will systematically investigate both extremely and late preterm infants for exercise-induced bronchoconstriction and for improvements in symptoms. The results will accurately delineate the reversible respiratory disease in preterm born children and will provide the urgently required evidence-base to guide treatment of respiratory disease for children who were born preterm – both extremely and moderately.

2.2 Aims and Objectives

The overall aim is to establish the underlying mechanisms of chronic airway obstruction observed in symptomatic children who are born preterm and to establish if there are different phenotypes of this condition that do or do not respond to standard inhaled therapy. The specific objectives are:

Primary

To assess if 12-week treatment with:

- bronchodilator (salmeterol) and anti-inflammatory (fluticasone)
- anti-inflammatory (fluticasone) alone

modifies the underlying mechanisms and/or improves airway obstruction when compared to placebo

Secondary

- Obtain repeat questionnaire responses to re-assess respiratory and neurological outcomes in this cohort and to investigate longitudinal changes in these data.

- To identify and characterise the mechanisms of airway obstruction in preterm-born children (≤34 weeks of gestation) aged 7-12 years old including assessing the role of atopy, airway inflammation (neutrophilic or eosinophilic), smooth muscle reactivity/bronchial hyper-reactivity, structural abnormalities, cardiorespiratory responses including bronchoconstriction during exercise, response of any post-exercise bronchoconstriction to β_2 adrenoceptor agonists and identifying candidate genes which may be important in

mediating response to inhaled treatment.

- To study the pulmonary structure and function by hyperpolarised gases (^{3}He and ^{129}Xe) MRI in (a) preterm-born children with FEV1 ≤85% and (b) preterm and (c) term controls without airway obstruction.

2.3 Potential Benefits and Risks

The medications used in this study are subject to marketing authorisations, and will be prescribed within their licensed indications. The management of any respiratory symptoms or exacerbations will be in accordance with usual clinical practise and the research nurse, research fellow and Investigator will be available throughout the study to discuss specific issues with participants. Any patient can withdraw from the study at any time with no detriment to future care. All ethical aspects of the study will be discussed when informed consent is obtained. Appropriate participant information has been developed and will be discussed prior to entry in to each part of the trial. Families will be provided a copy of the information sheet and their signed consent/assent forms.

Potential benefits

This study will establish the underlying mechanisms of airway obstruction observed in preterm-born survivors and will also elucidate if interventions can modulate these mechanisms thus suggesting specific targeted therapies for this large vulnerable population.

Therefore the work will benefit prematurely born children and their parents, care givers, policy makers and will provide potential for commercial companies to develop targeted inhaled drug therapies.

Our recent systematic review showed reversibility in the decrements of FEV_1 to single doses of bronchodilators (Kotecha SJ 2015), however only one study investigated bronchodilator treatment for greater than two weeks (showing improvement) (Pelkonen 1997) but this is limited by small numbers. Only one longer term study investigated inhaled corticosteroid treatment (showing improved bronchial lability) (Pelkonen 2001). It is very likely that we shall identify several phenotypes including (a) responders who

should be treated with standard inhaler treatment including (i) those who have "classical asthma" and (ii) those whose airway obstruction is consequent to preterm birth; and (b) non-responders who may not be on treatment or may even be prescribed chronic high dose ineffective corticosteroid therapy which is associated with adverse effects including growth suppression. The optimal management strategy remains unclear whether bronchodilators are more effective than corticosteroids especially as the underlying mechanisms remain poorly understood. There are isolated reports of oxidant damage or neutrophilic inflammation in ex-preterm children with respiratory disease (Teig 2013, Fillippone 2007) but it is unclear if these are primary or secondary effects or if they are altered by treatment.

Potential Risks

The potential risks, special warnings and precautions for the use of the two products used in the interventional part of the study (part 2) are detailed in the relevant Summary of Product Characteristics (SPCs). Our recent systematic review did not identify any adverse events (Kotecha SJ 2015).

We do not believe there are any applicable risks to participation in part 1 (screening) or part 3 (MRI study).

3 TRIAL DESIGN

The study is centred around a single-centre, double blind, double-dummy, randomised, placebo controlled trial.

3.1 Primary Outcome

The primary outcome will be the difference in pre and post treatment percent predicted FEV_1 after 12 weeks of therapy between the active groups and placebo

3.2 Secondary Outcomes

1) Differences in measures of obstructive airway disease (pulmonary function tests)
2) Differences in response to exercise challenge between treatment groups.
3) Differences in biomarkers of airway inflammation between treatment groups
4) Differences in respiratory and neurological symptoms (questionnaire)
5) MRI parameters: apparent diffusion coefficient (ADC) between 3 comparison groups (preterm, $FEV_1 \leq 85\%$ at baseline; preterm control, $FEV_1 \pm 1$ standard deviation from normal; and term control, $FEV_1 \pm 1$ standard deviation from normal).
6) Adverse events

4 STUDY POPULATION

From our cohort of 7000, we have 4200 children born preterm (<37 weeks gestation) and 2800 term-born controls (≥37 weeks gestation) for questionnaire survey; 1000 children (resident in south Wales) aged 7-12, born at a gestational age of ≤34 weeks, to be screened in part 1; 200 children with FEV_1 ≤85% predicted to be randomised in part 2; approximately 60 with FEV_1 ≤85% predicted to be included in part 3. A sub-set of preterm-born and term-born controls identified during screening (part 1) will also be asked to participate in part 2 (visit 1 only, approximately n=100 in each group) and part 3 (n=20 in each group).

4.1 Part 1

4.1.1 Inclusion

1) Children aged 7-12 at the time of screening
2) Born at a gestational age ≤34 weeks (NB. Approximately n=100 term controls will also be invited)
3) Resident in the south Wales area whom, in the opinion of the Investigator, are possible to follow up
4) Fully informed proxy consent from parents/guardians and assent from child where possible

4.1.2 Exclusion

1) Respiratory tract infection within the last three 3 weeks (will be asked to consider participating at a later date)
2) Congenital abnormalities
3) In the opinion of the Investigator:
   - severe cardiopulmonary defects, or
   - neuromuscular disease, or
   - severe neurodevelopmental impairment

   Which prohibit the possibility of compliance with the study protocol

4.2 Part 2

4.2.1 Inclusion

1) As part 1
   plus
2) Preterm-born children found during part 1 to have FEV1 ≤85% predicted
   NB. Approximately N=100 preterm-born (FEV_1 >85% predicted) and N=100 term-born controls (FEV_1 >90% predicted) will also be invited to Part 2 visit 1

4.2.2 Exclusion

1) As part 1
   plus
2) Known hypersensitivity to salmeterol and/or fluticasone

4.3 Part 3

4.3.1 Inclusion

1) Preterm-born children found to have FEV_1 ≤85% at baseline laboratory visit (part 2, visit 1) who participated in the treatment trial
2) Willing to travel to the University of Sheffield Academic Unit of Radiology, Royal Hallamshire Hospital

NB. Approximately n=20 preterm-born and n=20 term-born controls will also be invited to Part 3

4.3.2 Exclusion

1) Surgery of any type in the previous 2 months
2) Previous brain surgery involving placement of clips or shunts
3) Eye injuries resulting from metal fragments
4) Presence of any metal implants including pacemakers
5) Possibility to be or known to be pregnant

5 ASSESSMENTS AND PROCEDURES

5.1 Questionnaire study

A respiratory and developmental questionnaire will be sent to approximately 7000 families in Wales (4200 preterm-born and 2800 term-born children) who have previously given consent to be contacted regarding further research (RANOPS, MREC 12/WA/0155).

For 1000/4200 families of preterm-born children resident in south Wales the package of information will include a parent information and consent form (PISC) and child information and assent forms (CISC) with an invitation to take part in the home screening visit. A reminder (either by telephone call or repeat mailing) will be scheduled in the event of non-response at approximately 6 and 12 weeks; additional numbers shall be invited if initial uptake is not sufficient to meet the recruitment target for part 2. Sufficient term-born children (≥37 weeks) will also be invited for screening order to establish a control population of 100 consenting participants.

In order to increase the pool of potential participants, we shall also contact additional families of children, who were not originally sampled in the previous study (RANOPs), through our additional research sites and participant identification centres (PICs). Namely, children born preterm in Wales between 2004 and 2012. Each research site or PIC will, using contact information provided by the NHS Wales Informatics Service, extend the invitation to the study on behalf of their local health board. Personal identifiable information of those invited will not be transmitted to the research team, whom will only make contact with participants once consent has been obtained to do so (by return of the respiratory and developmental questionnaire). Research sites or PICs will be notified of non-responders by the research team in order to send a reminder mailing at approximately 6 weeks.

Those returning the questionnaire and expressing an interest in participating in the screening home visit will receive a follow-up phone call to assess baseline eligibility before organising a date and time for the screening visit.

5.2 Part 1: Screening

Part 1 of the study will look to ascertain the proportion of participants in the study population with reduced lung function (FEV_1 ≤85%) and to obtain anthropomorphic data on the cohort as a whole for use in phenotyping and hypothesis-generating exploratory analyses.

5.2.1 Screening visit

Prior to the screening visit, contact will be made to ensure that the child withholds the following where possible:

- Long-acting $ß_2$ agonists for 48 hours before visit before visit
- Inhaled corticosteroids for 24 hours before visit
- Short-acting $ß_2$ agonists for 8 hours before visit (unless symptomatic)
- Leukotriene receptor antagonists for 48 hours before the visit
- Caffeine for 24 hours before the visit
- Antihistamines for 48 hours before the visit
- Consumption of food or drink (except water) in the last hour
- Consumption of foods containing nitrate/nitrites on the day of testing

Informed consent/assent

On attendance at the screening visit (home or clinic), the research nurse will explain the purpose of the visit and place it in to context of the study as whole (i.e. explain that there are options for continued participation in parts 2 and 3). Informed consent and assent will be taken prior to any protocol procedures taking place.

Medical history

The research nurse will take a short medical history to document any relevant information relating to the study and to confirm eligibility. A short examination including temperature and oxygen saturation will be conducted.

Anthropometric measures

Weight will be measured using portable floor scales, and height recorded with a stadiometer. BMI will be calculated and adjusted for gender. Body composition will be

measured using bio-electric impedance. Pubertal status will be assessed using an orchidometer in boys and a short questionnaire in girls.

Cardiovascular assessment

Cardiovascular assessment will be performed using the Vicorder device (Smart medical, Gloucestershire, UK). Measurements of peripheral and central blood pressure will be obtained using a single brachial cuff.

Saliva sample

A saliva sample will be taken by asking the child to expectorate in to a container, or using a buccal swab for analysis of candidate genes.

Urine sample

A urine sample will be taken in to a 50ml universal container for later cotinine analysis.

Questionnaires

The completeness and accuracy of the respiratory and developmental questionnaire will be reviewed. The CHU-9D will be completed by the child. This is a short 9-item questionnaire regarding paediatric quality of life.

Spirometry

Percent predicted values (Global Lung Initiative reference ranges) based on age, gender, and height for Forced Expiratory Volume in 1 second (FEV_1), forced vital capacity (FVC), forced expiratory flow at 25 to 75% of the vital capacity (FEF_{25-75}) and peak expiratory flow (PEF) will be collected using a calibrated portable spirometer.

The forced expiratory methods will be demonstrated to each participant, who will be given the opportunity to practice the manoeuvre using an animated incentive tool designed to encourage maximal effort. Nose clips and mouthpieces with bacterial filters will be worn throughout testing. Each child will be encouraged to perform at least three acceptable and reproducible spirometric manoeuvres as per the ATS/ERS guidelines. Reproducibility will be assessed visually by observing the flow-volume loops and by ensuring that the differences in the FVC and FEV_1 between the manoeuvres are within 5%.

Reversibility test

Participants will be tested for airflow-limitation reversibility by delivery of 400µg salbutamol (4x 100µg actuations) from a metered-dose inhaler via a paediatric spacer (Volumatic, Allen & Hanburys, UK) with an appropriately sized mouthpiece. Children will be instructed to take 10 normal breaths through the device for each actuation of the inhaler (approx. 30 second intervals). Spirometry will then be repeated 10-15mins following dosing. The batch number and expiry date of the salbutamol inhaler used will be recorded on the CRF. The inhaler will be used until depleted however the spacer will be cold-water sterilised prior to re-use.

Results from the reversibility test will **not** be used as criteria for eligibility to part 2 of the study, but may be used in later data analysis to investigate a sub-group of participants identified as having reversible airways obstruction.

Exhaled Nitric Oxide

Fraction of Exhaled Nitric oxide (FE_{NO}), a marker of airway inflammation, will be measured using a NiOX chemiluminescence analyser (NiOX VERO, Aerocrine, Sweden). The participant is first asked to exhale to ERV, then, inserting the mouthpiece, inhales NO-free air until Total Lung Capacity (TLC). The participant then exhales slowly at a constant flow rate of approximately $50mL\cdot s^{-1}$ for 10 seconds. An incentive cartoon program encourages maintenance of a constant flow rate. The NO level is identified as a plateau lasting greater than 2 seconds. The average of three measurements within 10% (or 2 within 5%), taken at greater than 30s intervals, will be reported as per ATS/ERS standards.

Eligibility for Part 2

Preterm-born children found to have reduced lung function (FEV1 ≤85%), regardless of reversibility status, will be referred for participation in part 2 of the study.

Lung function data collected during Part 1 screening is for referral purposes only; the results of the lab-based spirometry (% predicted FEV_1) at Part 2 visit 1 will be used to make the decision on eligibility for randomisation to the treatment trial.

Approximately n=100 preterm-born children with a % predicted FEV_1 >85%will be invited to take part in Part 2 visit 1 only as controls.

Approximately n=100 term-born children with a % predicted FEV_1>90% will be invited to take part in Part 2 visit 1 only as controls.

5.3 Part 2

Preterm-born children participating in part 2 of the study will attend two clinic visits lasting approximately 3-4 hours each, during which they will complete an array of lung physiology tests as detailed below. Visits 1 and 2 will be separated by the 12-week (up to 16 weeks to ensure convenience of children and parents) treatment trial. Wherever practically possible, visits 1 and 2 will be conducted at approximately the same time of day (morning or afternoon session). Control participants (preterm and term) will attend visit 1 only.

5.3.1 Visit 1

Prior to visit 1, telephone contact will be made to ensure that the child withholds the following (where possible):

- Long-acting $ß_2$ agonists for 48 hours before visit before visit
- Inhaled corticosteroids for 24 hours before visit
- Short-acting $ß_2$ agonists for 8 hours before visit (unless symptomatic)
- Leukotriene receptor antagonists for 48 hours before the visit
- Caffeine for 24 hours before the visit
- Antihistamines for 48 hours before the visit
- Consumption of food or drink (except water) in the last hour
- Consumption of foods containing nitrate/nitrites on the day of testing

It will also be ascertained if the participant has had a recent respiratory tract infection within the last 3 week. If yes, they will be booked to attend a later date.

Informed consent/assent

Participant information and letters to participate will be provided in advance (after conclusion of the part 1 visit for both parent(s)/guardians(s) and children. On

attendance at the clinic, informed consent and assent will be taken prior to any protocol procedures taking place.

Questionnaires

A short questionnaire, focussed on healthcare service use, time off school and time off work will be completed by the parent(s)/guardian(s). The CHU-9D will be completed by the child.

If part 1 visit 1 is > 3 months after the screening respiratory and neurological questionnaire was completed, this will be repeated.

Physical exam/anthropometry

A physical exam will be performed by a qualified paediatrician/paediatric nurse including measurement of temperature oxygen saturation.

Weight will be measured using calibrated floor scales, and height recorded with a stadiometer. Body composition will be analysed using bioelectric impedance. Pubertal status will be estimated using an orchidometer in boys (testicular volume) and a questionnaire in girls

Saliva sample

A repeat saliva sample will be obtained to ensure adequate quality in participants who will be receiving the study intervention

Urine collection

Approximately 20ml urine will be collected in a universal container for later cotinine analysis.

Cardiovascular assessment

Cardiovascular assessment will be performed using the Vicorder device (Smart medical, Gloucestershire, UK). Measurements of peripheral and central blood pressure will be obtained using a single brachial cuff.

Skin Prick test:

Allergies to cat, house dust mite and grass pollen, in addition to positive (histamine) and negative (glycerol) control, will be applied to forearm and pricked using a microlancet. After 15 minutes, response will be reviewed and wheel size measured

with ≥3mm indicating a positive response. Atopy will be defined as a positive response to 1 or more of the allergens.

Spirometry

Percent predicted values based on age, gender, and height for Forced Expiratory Volume in 1 second (FEV_1), forced vital capacity (FVC), forced expiratory flow at 25 to 75% of the vital capacity (FEF_{25-75}) and peak expiratory flow (PEF) will be collected using a calibrated spirometer.

The results of the lab based spirometry (% predicted FEV_1) will be used to make the decision on eligibility for randomisation to the treatment trial; data collected during Part 1 screening is for referral purposes only.

The forced expiratory methods will be demonstrated to each participant, who will be given the opportunity to practice the manoeuvre using an animated incentive tool designed to encourage maximal effort. Nose clips and mouthpiece bacterial filters will be worn throughout testing. Each child will be encouraged to perform at least three acceptable and reproducible spirometric manoeuvres as per the ATS/ERS guidelines. Reproducibility will be assessed visually by observing the flow-volume loops and by ensuring that the differences in the FVC and FEV_1 between the manoeuvres are within 5%. All flow-volume loops will be assessed for compliance to guidelines by an experienced respiratory physiologist or paediatric respiratory clinician.

Exhaled Nitric Oxide (FE_{NO})

FE_{NO}, a marker of airway inflammation, will be measured using a NiOX chemiluminescence analyser (NiOX VERO, Aerocrine, Sweden). The participant is first asked to exhale to ERV, then, inserting the mouthpiece, inhales NO-free air until Total Lung Capacity (TLC). The participant then exhales slowly at a constant flow rate of approximately $50mL \cdot s^{-1}$ for 10 seconds. An incentive cartoon program encourages maintenance of a constant flow rate. The NO level is identified as a plateau lasting greater than 2 seconds. The average of three measurements within 10% (or 2 within 5%), taken at greater than 30s intervals, will be reported as per ATS/ERS standards.

Forced Oscillation Technique

Measures of airway reactance and resistance will obtained with using the FOT method. The test requires a short period of tidal breathing through the mouthpiece attached to

the device. A loudspeaker generates a series of impulses which travel through the airways- this is repeated at a range of frequencies in order to measure behaviour of the different sized airways.

Whole body plethysmography

Measures of Functional Residual Capacity (FRC) and Specific Airway Resistance (sR_{aw}) will be collected using whole body plethysmography. In the manoeuvre, the participant will be asked, wearing a noseclip and supporting both cheeks with their hands, to establish a period of regular tidal breathing for approximately 5-10 seconds. When at or approaching FRC, the airflow is occluded using a shutter, and the child will be asked to perform a series of gentle pants. Once the shutter is released, the participant slowly breathes out to Expiratory Reserve Volume (ERV) and breathes back in to Inspiratory Reserve Volume (IRV).

Inert gas washout

Inert gas washout will be used as an additional measure of FRC and to assess ventilation perfusion inhomogeneity (lung clearance index). The participant, wearing a nose clip, will be asked to breathe tidally for approximately 1 minute, after which the breathing circuit will be opened to the test gas. Tidal breathing continues until the change in inert gas concentration equilibrium is reached, after which the test gas is removed from the circuit.

Exhaled Breath Condensate (EBC)

Collection of EBC is a simple non-invasive method of sampling the respiratory tract. The participant will be asked (whilst wearing a noseclip) to breathe tidally through the cooling tube (Rtube, Respiratory research Inc., Texas USA) for 15 minutes, stopping briefly if they need to swallow salvia.

Exercise challenge

Maximal exercise capacity will be measured using a cycle ergometer to ascertain peak oxygen consumption (VO_2), peak carbon dioxide output (VCO_2) and minute ventilation (V_E) in the last 15 seconds of exercise. Participants will be asked to wear a facemask, connected to the gas analyser, and a telemetric heart rate monitor. The child will be instructed to pedal at an unloaded constant rate of 60 rpm. The load will be increased over the duration of the test until 2 of the following criteria are met:

1) maximum heart rate is between 80-90% of predicted maximum (220bpm-age)
2) respiratory exchange ratio is >1
3) peak oxygen consumption rate (VO_2) plateau was reached
4) volitional exhaustion was present as assessed by Borg's scale of perceived exhaustion

Spirometry will be performed at 5, 10, 15, 30 and 40 minutes to assess if exercise-induced bronchoconstriction has occurred. 400µg Salbutamol will be given using a spacer (Volumatic, Allen & Hanburys, UK) to assess the reversibility of any obstruction, with the FEV_1 manoeuvre being repeated 15 minutes following the final salbutamol dose. The batch number and expiry date of the salbutamol inhaler used will be recorded on the CRF. The inhaler will be used until depleted however the spacer will be cold-water sterilised prior to re-use.

Sputum Induction

Sputum will be collected to assess cellular airway inflammation. Participants will be asked to inhale ultrasonically nebulised 3-7% hypertonic saline (Nebusal 7%, Forest Laboratories, UK; or Mucoclear 4 & 6%, PARI Medical, UK) in 5 minute intervals for 20 minutes. After each inhalation, the child will be asked to cough and expectorate any sputum into a container. FEV_1 will be measured prior to the start of the test, and must be within 90% of baseline value prior to beginning induction. Otherwise, a further dose of salbutamol 4x100µg will be administered. FEV_1 will also be assessed following each of the inhalation periods. Any participant exhibiting a decline in FEV_1 <75% predicted will be discontinued and bronchodilators administered if necessary.

Randomisation to trial treatment

On completion of the Part 1 visit 1 schedule of assessments, eligibility will be confirmed by the attending study clinician and recorded on the CRF; the participant will be randomised to receive either monotherapy (fluticasone), combination therapy (fluticasone/salmeterol) or placebo. The IMP will be prescribed by the attending study clinician or qualified nurse prescriber.

Inhaler technique will be demonstrated by the attending members of the clinical and nursing team.

5.3.2 During the treatment period

During the 12 week period of treatment children participating in the RCT will be asked to perform peak expiratory flow twice per day (prior to taking the morning and evening inhaler). We shall also ask the preterm and term control groups to collect 4 weeks of peak flow data in the same manner for comparison. The peak flow meter and training to perform the manoeuvre will be provided by the research nurse or medic at the conclusion of part 2 visit 1.

At least one telephone consultation will be conducted by the research nurses to check treatment compliance and to monitor any new, or follow-up on, adverse events. A dedicated telephone line will be available for participants to contact a member of the trial team (research medic or nurse) during working hours (9-5 Monday to Friday). Further calls will be made if the initial call raises issues related to compliance or adverse events. All reported adverse events will be reviewed by the Investigator or designee as described in section 8.

5.3.3 Visit 2

All tests will be repeated at visit after the 12 week treatment trial with the exception of saliva sampling and skin prick testing.

Families will be asked to return all used and unused inhalers for treatment compliance and IMP reconciliation checks.

Eligibility for part 3

All children who participated in Part 2 will be eligible to participate in Part 3 of the study.

Approximately n=20 of the term-born and n=20 preterm-born controls will also be invited to part 3.

5.4 Part 3

MRI Scan

Families eligible for part 3 will be invited to attend the clinic at the Academic Unit of Radiology, University of Sheffield, Royal Hallamshire Hospital, to undergo hyperpolarised xenon MRI scanning.

For participants who are born prematurely (at 34 weeks or less) and took part in the inhaler study and are agreeable, a MRI scan will be performed prior to, and after participants have received a bronchodilator_to show if we can open up the breathing tubes for the gas to enter. Those agreeing to participate will have travel, accommodation and subsistence costs covered by the project funds.

Inert gas washout

Inert gas washout will be used as an additional measure of FRC and to assess ventilation perfusion inhomogeneity (lung clearance index) at the University of Sheffield. The participant, wearing a nose clip, will be asked to breathe tidally for approximately 1 minute, after which the breathing circuit will be opened to the test gas. Tidal breathing continues until the change in inert gas concentration equilibrium is reached, after which the test gas is removed from the circuit

Informed consent/assent

Participant information and letters to participate will be provided in advance (after conclusion of the part 2 visit(s) for both parent(s)/guardians(s) and children. On attendance at the unit, informed consent and assent will be taken prior to any protocol procedures taking place. Screening checklists will be completed to ensure children are suitable for scanning as detailed in section 4.

5.5 Biological samples

Saliva

Saliva samples will be obtained either by direct expectoration in to the collection container or by use of buccal swabs. All samples will be stored at ambient temperature prior to DNA extraction and analysis. DNA will be scanned for candidate genes related to airway obstruction and inflammation and will also be used to contribute to Genome-

wide Association Studies. Consent will be specifically sought for the ongoing storage, and use of DNA. Saliva samples may also be used to compare the metabolomic profiles of groups of participants (e.g. preterm vs. term, pre-and post RCT intervention)

Exhaled Breath Condensate

The resultant sample will be immediately aliquoted and stored at -70°C pending analysis

Urine

The resultant sample will be aliquoted and stored at -70°C pending cotinine and inflammatory marker analysis

Sputum

After collection, sputum will be placed on ice prior to processing (within two hours of collection). The sample will be placed in a petri dish and sputum plugs will first be separated from saliva.

The selected sputum will be processed in accordance with established guidelines. A proportion of the cell suspension will be used for total cell count. Cell viability will be reported as a percentage of the total cell count.

The remainder of the suspension will then be centrifuged and supernatant will be aspirated for storage at -70°C. The cell pellet will be prepared for cytospin and fixed to slides in order to provide a differential cell count.

6 TRIAL TREATMENT

Eligible participants will be randomised to receive blinded study medication, stratified by inhaled steroid status: not currently taking inhaled steroids (non-ICS) or weaned from inhaled steroids (see section 9.4). Non-ICS participants, expected to comprise approximately 87% of the eligible children, will be randomised to:

a) Combination therapy- Inhaled long acting β_2 agonist (25µg salmeterol xinafoate, total daily dose of 100µg) and corticosteroid (50µg fluticasone propionate, total daily dose of 200µg);

b) Monotherapy- Inhaled steroid only (50µg fluticasone propionate, total daily dose of 200µg);

c) Placebo.

Participants who have been successfully weaned off steroids prior to visit 1 of part 2, expected to comprise approximately 15% of children, will be randomised to:

a) Combination therapy- Inhaled long acting β_2 agonist (25µg salmeterol xinafoate, total daily dose of 100µg) and corticosteroid (50µg fluticasone propionate, total daily dose of 200µg);

b) Monotherapy- Inhaled steroid only (50µg fluticasone propionate, total daily dose of 200µg)

6.1 Formulation, packaging and supply

Serevent® (25µg salmeterol xinafoate), and Flixotide® (50µg fluticasone propionate) metered-dose inhalers will be used for the active arms of the trial. The drug suspensions are contained in an internally lacquered, 8ml aluminium alloy pressurised container sealed with a metering valve. The containers are fitted into plastic actuators (identical aside from colour) incorporating an atomising mouthpiece and fitted with dustcaps. One pressurised container delivers 120 actuations. A matching placebo canister, containing no active substances, will be sourced from GlaxoSmithKline (GSK). The remaining excipients (hydrofluroalkane as propellant) are identical between active and placebo canisters. The placebo canisters will be QP released to St Mary's Pharmaceutical Unit, Cardiff, who will produce and QP release the finished IMP.

For blinding purposes, all canisters will be removed from their original actuators and placed in plain-coloured actuators provided to the IMP manufacture by GSK. GSK has provided evidence of the equivalency of Serevent and Flixotide actuators. These inhalers have an expiry date of August 2018.

Further inhalers will be procured in early 2018 for the remainder of the study. GSK will provide the inhalers directly. The active inhalers are identical and will be provided 'off the shelf', with no manufacturing of the products required. The placebo inhalers' contents are exactly the same as those produced by GSK and are a CE marked product.

First batch (expiry August 2018)

Treatment packs will be produced containing 3 sets of 2 inhalers: 3 sets are sufficient for the treatment period. A further back-up set is to be manufactured and be retained as a reserve (e.g. in the event one set is lost during the treatment period) (Figure 2a). Each participant will also be provided with a salbutamol inhaler, for use as rescue treatment (taken as required).

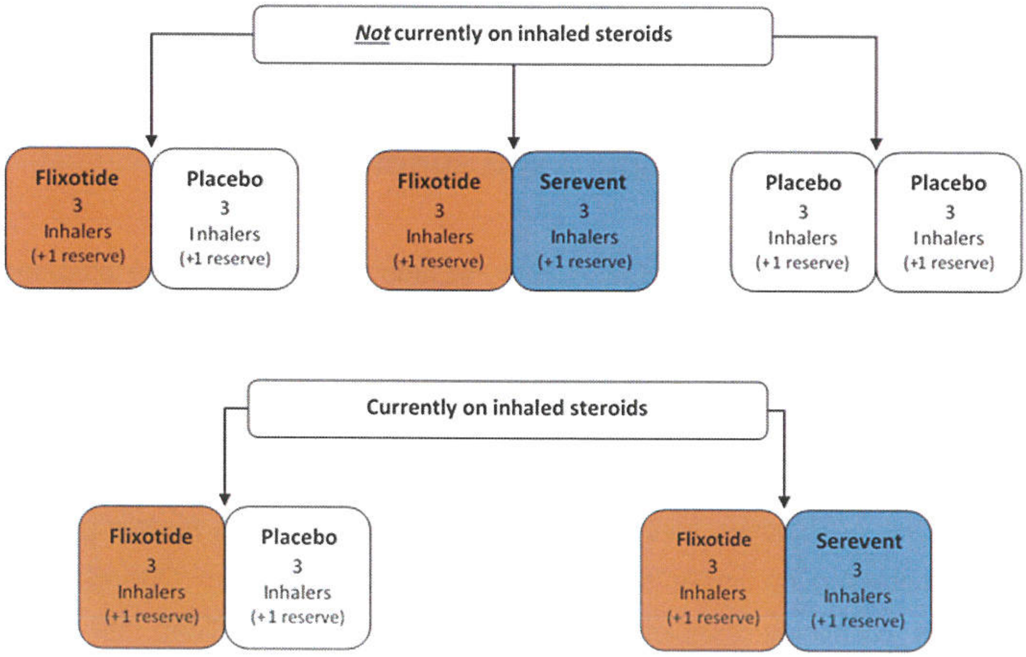

Figure 2a Schematic of double-dummy design

Second batch (produced early 2018)

For the further inhalers procured in 2018, treatment packs will be produced containing 4 sets of the 2 inhalers to ensure children do not run out inhalers beyond the 12-week treatment period. Again, a further back-up set is to be manufactured and retained as a reserve (Figure 2b).

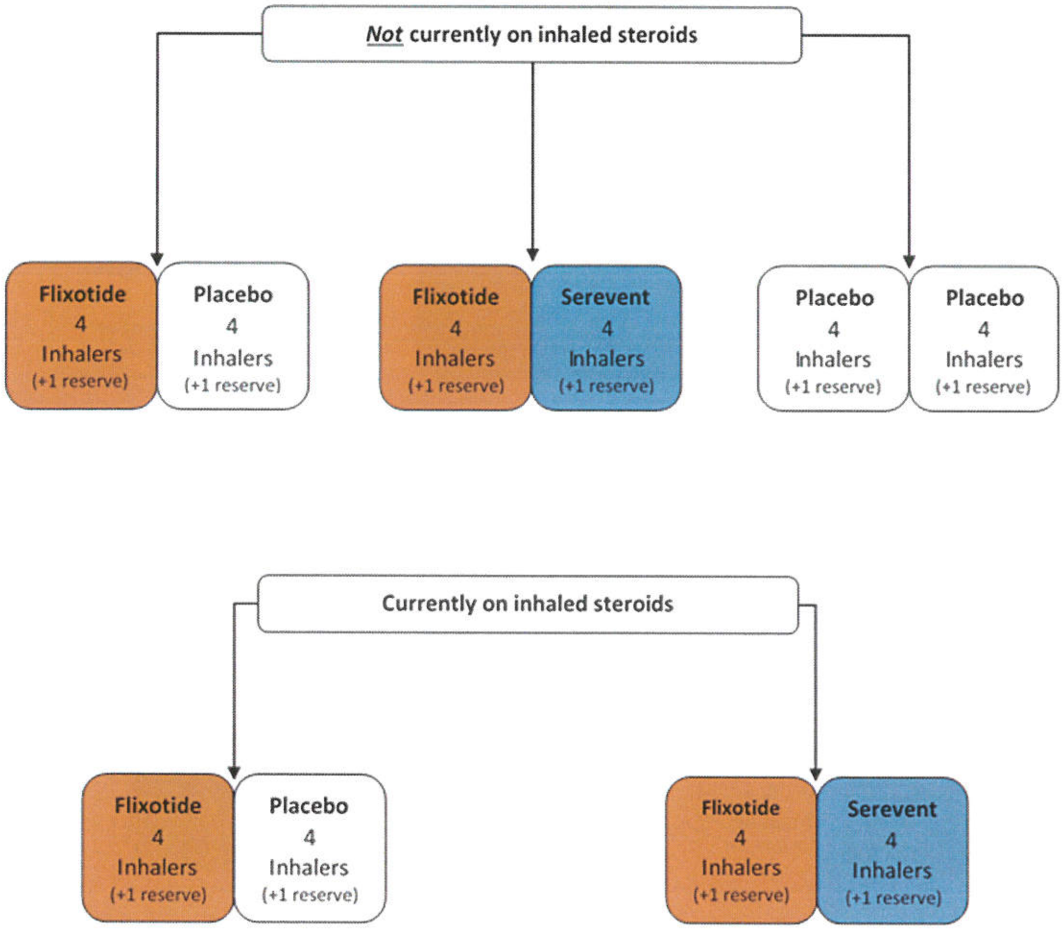

Figure 2b Schematic of double-dummy design for further procurement of inhalers

Third batch (produced late 2018)

For the further inhalers procured in 2018, treatment packs will be produced containing 4 sets of the 2 inhalers to ensure children do not run out inhalers beyond the 12-week treatment period. Again, a further back-up set is to be manufactured and retained as a reserve (Figure 2c).

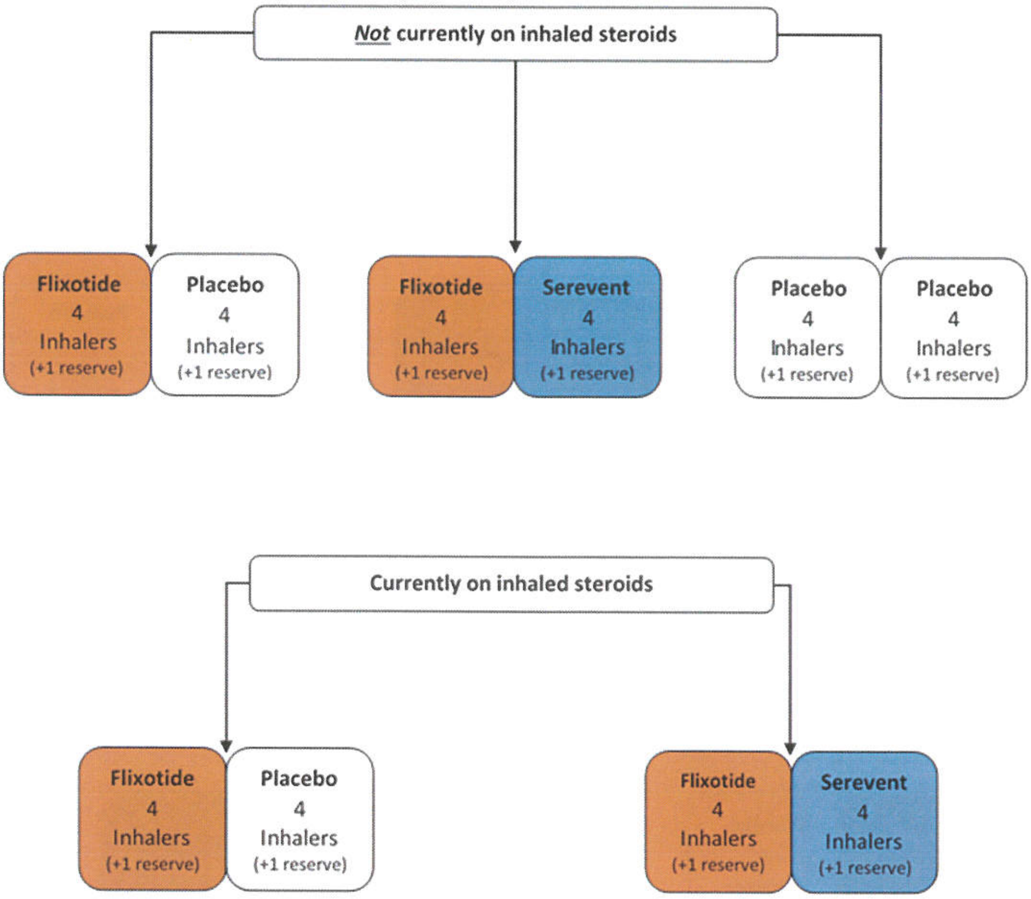

Figure 2c Schematic of double-dummy design for further procurement of inhalers

Labelling will be in compliance with Annex 13 of GMP according to the Treatment Pack Codes on the randomisation lists provided by the NWORTH statistics team. Each inhaler (canister and actuator) will be labelled as A (first product as named in the randomisation list), or B (second product as named in the randomisation list). Each inhaler will be packaged in a secondary container, also labelled as A or B, which will constitute the final IMP for QP release. Post QP certification labelling and packaging

for safety purposes will then be undertaken to package the secondary containers in an outer box which will be similarly labelled and tamper-evident sealed.

Treatment Pack Codes will be in the format XXXYYY, where XXX is a site identifier and YYY is a sequential number starting with 001.

Batches of treatment packs will be delivered to the pharmacy at the University Hospital of Wales and stored in a temperature and access controlled area at a temperature not exceeding 25°C. For the further procurement of inhalers in 2018, the treatment packs will be stored at a temperature not exceeding 30°C. A limited number of packs will be issued to delegated members of the study team for storage in the RHiNO laboratory in order to allow out-of-hours randomisation. Storage will be in a lockable cupboard within an access-controlled room. The storage area will be monitored with a calibrated thermometer.

6.2 Prescription and administration of trial treatment

Treatment packs will be prescribed to participants (as allocated by the online randomisation system) by the Investigator or appropriately trained designee.

Participants will be instructed to inhale two actuations, twice daily (in the morning and evening), *from each inhaler*, through a spacer device (given to each participant; Volumatic, Allen & Hanburys, UK). Training will be given by the research team in good inhaler technique, with use of practise doses using a dummy inhaler where deemed necessary. Participants will be instructed that the inhalers must be taken as prescribed and technique will be assessed by the study nurse prior to ending the visit.

6.3 Storage and stability

All IMPs and nIMPs (see 6.8) will be stored in an access-controlled, lockable cupboard at a temperature not exceeding 25°C. For the further procurement of inhalers in early 2018, the treatment packs will be stored at a temperature not exceeding 30°C. The temperature of the cupboard will be continuously monitored using a calibrated electronic data logger which will alarm above the set temperature. The device will also

display the current temperature and the maximum and minimum values of the period since it was last reset. When IMP is removed from the cupboard, the temperature will be noted a log sheet and the device reset. Thus, the study team will be aware of any temperature deviation which has occurred since the last issue of IMP.

In the event a temperature excursion is identified, any IMP or nIMP affected will be withdrawn and quarantined before return to pharmacy for destruction.

Records of the storage temperature will be downloaded monthly from the data logger and records kept in the Investigator Site File (ISF).

6.4 Returns and compliance

All participants will be instructed to return used in inhalers for accountability checks at the conclusion of the trial treatment (end of part 2). These will be returned to University Hospital of Wales pharmacy where, along with any unused medication, it will be destroyed periodically, in batches, in accordance with local policy for disposal of medicines on permission from the Sponsor.

If participants wish to prematurely withdraw from the trial, the study nurse will collect all used and unused medication and packaging if the participants do not wish to attend for Part 2, visit 2.

6.5 Unblinding

6.5.1 Unblinding of Individual Participants During the Trial

Breaking the statistical blind should be considered only when knowledge of the treatment assignment is deemed essential for the participant's care by the participant's physician or a regulatory body; In general, unblinding of participants during conduct of the clinical trial is not allowed unless there are compelling medical or safety reasons to do so.

N.B. If simply ceasing study treatment is a viable option for the participant's care, it should not be necessary for unblinding to occur.

6.5.1.1 Procedure

a. The decision to unblind a single case should be made when knowledge of an individual's allocated treatment is essential to:
    i. Enable treatment of severe adverse event/s, or
    ii. Enable administration of another therapy that is contraindicated by the trial treatment
b. *Where possible* (during office hours), consent for individual unblinding should be made via NWORTH who will seek agreement of the Investigator
c. Unblinding codes will be provided by NWORTH in the form of tamper-evident envelopes (one for each participant) which will be stored in a secure access-controlled room at the study site
d. Individual participants only are to be unblinded and the following is to be documented:
    i. Date information needed
    ii. Detailed reason for unblinding
    iii. Identity of recipients of the unblinding information
e. Allocation should not routinely be revealed to NWORTH personnel or the trial team

6.5.2 At completion of Treatment Phase

Participants will be encouraged to make an appointment with their GP to discuss future treatment options, and will be provided with a management plan from the study team. The family will be made aware of this procedure in the information sheets and at their final study visit. On completion of the trial, families will have the opportunity to obtain details of their treatment allocation following publication of the main study report.

6.6 Accountability Procedures for Study Treatment/s

Treatment packs will be stored at the manufacturer and released for storage at the University Hospital of Wales Pharmacy on a periodic basis. Packs will be released to

patients on completion of a prescription by the Investigator or designee. A detailed accountability log will be kept detailing the receipt, issue and return of the medications from storage at pharmacy.

6.7 Assessment of Compliance with Study Treatment/s

Participants will be asked to return all inhalers and used treatment pack for compliance checks at Part 2, visit 2.
Participants will be asked, with support from parent(s)/guardian(s), to complete a daily diary of treatment compliance. This will be reviewed at part 2, visit 2. In addition, a minimum one follow-up phone call performed by a research nurse will enquire regarding any issues with treatment compliance.

6.8 Non-Investigational Medicinal Products

Salbutamol has been identified as a non-investigational Medicinal Product (nIMP), and will be obtained from the site pharmacy. It has a marketing authorisation in the UK and will be used within this authorisation for two purposes:

a) To induce a physiological response (bronchodilation) during the reversibility testing in part 1 and part 2
b) To be issued as a rescue treatment as part of the treatment pack supplies to randomised participants, and also if required during the part 2 laboratory visits

Storage conditions will be as per IMPs (section 6.3). For Salbutamol used in part 1 and part 2 reversibility tests, the batch number and expiry date of the inhaler used will be recorded on the CRF. For salbutamol used as a rescue treatment, this will be packaged and labelled alongside the IMP in treatment packs. The supplier shall ensure the expiry date of the salbutamol inhaler matches or exceeds that of the packaged IMP.

6.9 Concomitant medications/Treatments

Details of concomitant medications will be collected during part 1, and during the medical history taken at visit 1 of part 2. These will be reviewed at each follow-up phone call and the 2nd clinic visit. The study treatments have very few adverse interactions

with other medicinal product so concomitant medications, with the exception of those listed below, are permissible at the discretion of the Investigator.

Medications not permitted whilst receiving study intervention (unless prescribed by health professional in the event prescribed IMP is not controlling symptoms, or in an emergency)

- Inhaled corticosteroids (other than the study treatment)
- Long-acting $ß_2$ agonists (other than the study treatment)
- Leukotriene receptor antagonists
- All beta-blockers
- Theophylline

Each treatment pack will also contain a salbutamol inhaler for use as rescue treatment (taken as required).

6.10 Co-enrolment Guidelines

To avoid potentially confounding issues, at the point of randomisation, participants should not currently be, or recently (within past 3 months) have been taking part in trials of other Investigational Medicinal Products.

Participation in other forms of clinical research should be on prior agreement with the Investigator.

Table 1 Schedule of procedures

PART	Questionnaire study	1		2			3
**PROCEDURES**		Pre-screening	Screening visit	Laboratory visit 1	Telephone contact	Laboratory visit 2	^{3}HE MRI scan
Respiratory/developmental questionnaire	X			X†			
Inclusion/Exclusion checklist		X	X	X			X
Signed informed consent/assent			X	X			X
Medical history			X	X			X
Physical examination			X	X		X	
Cardiovascular assessment			X	X		X	
Spirometry			X	X		X	
Reversibility test			X	X		X	
Saliva sample			X	X			
Body plethysmography				X		X	
Exhaled nitric oxide			X	X		X	
Multiple breath Washout				X		X	X
Forced oscillation technique				X		X	
Cardiopulmonary exercise test				X		X	
Sputum induction				X		X	
Exhaled breath condensate				X		X	
Randomisation to treatment				X			
Quality of Life questionnaire			X	X		X	
Service use questionnaire				X		X	
Compliance monitoring					X	X	
Adverse event monitoring				X	X	X	X
MRI							X

† If >3 months since screening questionnaire was completed

7 STATISTICAL CONSIDERATIONS

A separate and full statistical analysis plan (SAP) will be developed prior to the analysis of the study data. The SAP will be agreed by the Trial Steering Committee before being sent to the Independent Data and Safety Monitoring Committee for comment and approval.

7.1 Method of Randomisation

Randomisation will be completed using a dynamic adaptive randomisation system and will be stratified by inhaled steroid status (weaned from inhaled steroids or not currently taking inhaled steroids).

Randomisation will be performed by trained members of the research team through secure 24 hour web access to the randomisation system based at NWORTH. This system will be maintained and monitoring independently of the trial statistician.

Randomly generated lists will be provided to St Marys Pharmaceutical Unit / GSK by an independent member of the NWORTH team that state which Treatment Pack Codes relate to which medication. The outcome from the randomisation system will therefore simply be a Treatment Pack Code number, which is assigned to the participant. This will ensure that the trial team remain blinded when completing randomisations.

7.2 Sample size

7.2.1 Part 1

The main aim is to identify 200 children with FEV_1 ≤85% predicted by screening approximately 1000 children who were born preterm at ≤34 weeks' gestation. In addition we shall identify 100 preterm-born with FEV_1>85% and 100 term-born control children with FEV_1>90%.

It is expected from the RANOPS study (Edwards 2016) that the worst-case scenario would mean 600 children will fulfil the criteria to take part in the screening but it is expected the sample is more likely to be 1000 children. The main analysis for the screening part of the study is an exploratory regression model on this data set. There will be approximately ten variables for inclusion into the model to begin with and so this

worst-case scenario will be considered for the power calculation. Therefore, the power of a multiple regression model using an alpha of 0.05, ten independent variables with an R^2 value of 0.2 (to be conservative) and a sample of 600 participants results in a power of 100%. There have been no controlled independent variables included in the calculation as this model is being created from scratch with no variables automatically assumed for inclusion. There is sufficient power for this analysis with the minimum expected sample size and if all of the variables result in the model.

7.2.2 Part 2

Using the data from the ALSPAC study, (Kotecha 2012b) 21.8% of pre-term born children had predicted lung function of $FEV_1 \leq 85\%$ and it is expected that all of these children that are eligible will take part in the RCT. The RANOPS study (Edwards 2016) found that 13.3% of very pre-term children were using corticosteroid inhaler medication and therefore 86.7% are anticipated to be steroid-free. Those not on steroids will be allocated to either: placebo, monotherapy or combination therapy, whereas those already using corticosteroids can only be allocated to monotherapy or combination therapy. This gives an approximate final allocation ratio of 1:1.3:1.3 of placebo: monotherapy: combined therapy.

A sample size calculation using a one way ANOVA indicates that a sample of 53 (15:19:19) will have approximately 80% power to detect difference of 10 (assumed SD of 10) among the means using a one-way ANOVA at the 5% significance level. This assumes a 10% absolute difference in predicted percentage FEV_1, which is considered a minimal clinically relevant difference. Data from the ALSPAC study (Kotecha 2012b) has suggested the upper confidence interval for the standard deviation on the predicted percentage FEV_1 is 9.5 and current accumulated baseline data (02/10/2018) indicates that the standard deviation is around 9, therefore we have assumed a standard deviation of 10 to allow some additional variability of the data. Assuming an attrition of 20% would require a sample recruited of 67 (19:24:24). This sample would be the minimal recommended sample to achieve to detect differences of those noted. Allowing for a larger SD of around 11 will require a sample of 64 (18:23:23) inflated to 81 to include 20% attrition.

7.2.3 Part 3

A previous study (Diaz 2008) showed a maximum standard deviation of 0.06 on the primary outcome of ADC and a clinically significant difference would be considered at the 0.10 level. Given these parameters with an alpha of 0.05 and power of 80% on an ANOVA with three groups (preterm with reduced lung function, preterm controls and term controls), a sample of just 21 is required. As all children from the RCT will be invited to the MRI should they wish, which includes a sample of approximately 100 of preterm controls and 100 term controls, we should have more than sufficient numbers at this stage to detect a difference.

7.3 End of Trial

The end of the trial will be considered as last participant last visit

7.4 Analysis Plan

Part 1: Using the data from the screening section of the study, a regression model will be produced to evaluate whether any pre-defined variables relating to the child's characteristics are linked to their lung function.

Testing between the lung function of the children pre and post taking the bronchodilator will be assessed using a t-test to evaluate any effect this may be having.

Part 2: The RCT data will be used to evaluate whether either active treatment improves the lung function throughout the duration of the trial. Also tested will be whether the pre-term born children with airway obstruction have different lab data than those within either of the control groups. These comparisons will be made using ANOVAs and post-hoc t-tests where appropriate.

Regression models will also be produced in an attempt to predict the marked airway obstruction and drug responses in relation to specific characteristics. If this analysis shows that prior treatment has an effect on either of these outcomes then a further exploratory sub-group analysis will be completed.

Part 3: An ANOVA will be used to evaluate the difference in apparent diffusion coefficient (ADC) between the groups of pre-term born children with airway obstruction and the control children.

Analysis will be completed to assess if the ADC in groups is related to specified characteristics of the child.

7.5 Interim safety monitoring and analysis

RHINO will be monitored by an IDSMC. The IDSMC will be responsible for reviewing and assessing recruitment, interim monitoring of safety, trial conduct and external data. Missing data will be monitored and strategies developed to minimise its occurrence.

In initial analysis of the study data for IDSMC review is planned for 6 months after the first randomisation (or with a minimum of 50 participants enrolled)to assess screening vs. recruitment rates, and to undertake an internal pilot estimation of the standard deviation of the primary outcome at baseline. This will be checked against the standard deviation used in the sample size calculation to ensure it is within a sensible range. This blinded pilot is not deemed to have any significant impact on the final analysis. If the standard deviation is smaller than that used in the sample size calculation, suggested that fewer participants are required than initially proposed then no action will be taken and the size of the study will remain as planned. If the standard deviation is larger than assumed suggesting the need for more participants, then under advice from the IDSMC, the TSC will aim to increase recruitment and consider implications for funding and existing resources.

Subsequent timing of the next analysis of the data will be determined on the basis of recruitment rates at the initial IDSMC meeting although it is anticipated that his will be approximately half way through the accrual period. The IDSMC may request additional analyses if triggered by a concern regarding SUSARs. The IDSMC chair will receive details of SUSARs as they occur. All results of the above analyses in relation to investigation of SUSARSs will remain confidential to the IDSMC members and will not be for review by the TMG (except the statistical team preparing the IDSMC report).

8 PHARMACOVIGILANCE

The trial will be conducted in accordance with the Pharmacovigilance requirements set out in the Medicines for Human Use (Clinical Trials) Regulations 2004 plus amendments.

The following definitions and arrangements will apply to the coding and reporting of adverse events (AEs).

The Investigator or designee is required to use their clinical judgment in conjunction with the Reference Safety Information to assess which events are **untoward** and **unexpected** in each participant.

8.1 Terms and Definitions

Adverse Event (AE)
Is defined as any **untoward** medical occurrence in a participant to whom a medicinal product has been administered, including occurrences which are not necessarily caused by or related to that product.

Adverse Reaction (AR)
Is defined as an AE that in the opinion of the Investigator is suspected to be causally linked to the investigational medicinal products or placebo.

Serious Adverse Event (SAE)
Is defined as the occurrence of an AE where the death of the participant resulted or was otherwise threatened; or where the participant required prolonged hospital stay; or resulted in persistent or significant disability; or incapacity; or was a congenital anomaly/birth defect. Medical judgement may consider other adverse events fall also in this category.

Serious Adverse Reaction (SAR)
Is defined as a serious adverse event that is suspected to be causally linked to the study investigational medicinal product or placebo.

Suspected Unexpected Serious Adverse Reaction (SUSAR)
Is defined as any suspected adverse reaction to the investigational medicinal product or placebo that is unexpected and serious; i.e. its nature and severity are not consistent with the information described in the SPC.

Expectedness
Unexpected is defined as an event where the nature and severity is not consistent with the reference safety information.

8.2 Reference Safety Information

The SPCs for Serevent® (section 4.8) and Flixotide® (section 4.8) will be considered the Reference Safety Information for assessment of expectedness of adverse reactions. Each SPC will be checked monthly to check for any applicable updates.

8.3 Treatment withdrawal criteria

8.3.1 Stopping criteria

There are no statistically defined stopping criteria for the withdrawal of trial treatment. Adverse events will be assessed as described in section 8.4 and any decisions for individual participants will be made dependent upon the frequency, nature and severity of the event by the Investigator.

8.3.2 Consideration of reproductive potential and administration of IMP

The study is specifically designed to include pre-pubertal children by limiting the inclusion criteria to 7-12 year olds thus the chances of pregnancy in this group are extremely small. Nevertheless, pubertal staging will be assessed during the part 1 home screening visit and girls will be closely observed for any signs of pregnancy in any child who participates in the randomised controlled trial.

8.4 Reporting procedures

An algorithm is presented below, summarising the reporting requirements:

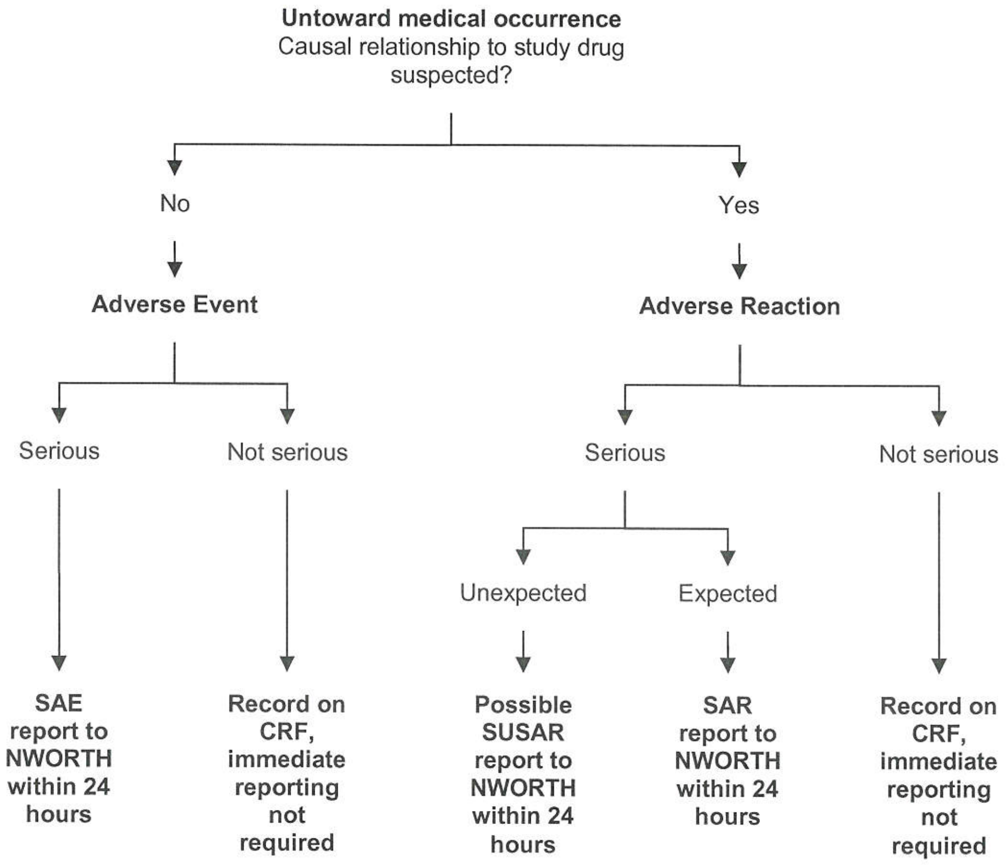

All adverse events fulfilling reporting criteria should be recorded on the CRF and submitted to NWORTH within the defined timelines, beginning from the time that written informed consent is obtained (i.e. prior to undergoing any study-related procedure and/or receiving investigational medicinal product) and continuing until the final study visit for that participant.

8.4.1 Non-Serious AEs

All such events, whether expected or not, should be recorded on the CRF

8.4.2 SAEs

NWORTH will be notified of potential SAEs at the earliest opportunity and within one working day of becoming aware of the occurrence and provide, at least, the minimum information required for reporting using the SAE report form:

Minimum information required for reporting:

- Study identifier
- Treatment pack code
- A description of the event
- Date of onset
- Current status in trial
- Whether study treatment was discontinued
- The reason why the event is classified as serious
- Investigator assessment of the association between the event and study treatment

- The Investigator or person with delegated responsibility will review all SAEs as reported within one working day of their receipt, and will consider causality and expectedness for each medicinal product. Where there is suspicion that an SAE is associated with any of the trial interventions, and is graded as unexpected, that event will be classified as a potential SUSAR.

- SUSARS will be confirmed by unblinding the participant at NWORTH. If a participant is found to be on active treatment, NWORTH will notify the Sponsor. For participants found not to be on active treatment (i.e. placebo), no notification to the Sponsor is necessary unless there is a SUSAR that may relate to components of the placebo.

- NWORTH will notify the MHRA and REC of all SUSARs within 7 days of first knowledge of the event if the SUSAR is linked to a death or considered life-threatening. Additional information sent within a further 8 days. All other SUSARs will be notified to the CA/REC within 15 days of first knowledge of the event.

- NWORTH will maintain a detailed record of all SUSARs reported to them. Details of this record will be provided by the study team in annual Development Safety Update Reports to the MHRA and REC.

8.4.3 SUSAR reporting in relation to nIMPs

SUSARs relating the nIMPs (see section 6.8) will only be reported where there is a possibility of an interaction between the nIMP and an IMP. If a SUSAR which can be linked to either the nIMP or IMP, but cannot be attributed to only one, this must be reported. In any case, if the SAR relating to the nIMP is likely to affect the safety of trial subjects, this will be reported to the competent authority as an urgent safety measure, a substantial amendment, or early termination of the trial, as appropriate under direction from the Sponsor.

8.5 Follow-up After SAEs

All reported SAEs should be followed until satisfactory resolution or until the Investigator responsible for the care of the participant deems the event to be chronic or the participant to be stable.
When reporting, the Investigator responsible for the care of the participant should apply the following criteria to provide information relating to event outcomes: recovered; recovering; not recovered; fatal; unknown; recovered with sequelae (specifying with additional narrative).

8.6 Nature and frequency of safety assessments

Monitoring of adverse events in relation to the IMP will begin from entry in to part 2 of the study through to final follow up (end of part 2 visit 2).

- Participants not weaned from inhaled corticosteroids will be contacted at least once during the treatment trial (see section 5.3.2).
- Participants weaned from inhaled corticosteroids will be contacted weekly (see sections 5.3.2 and 9.4).

All participants will advised as to the most common signs and symptoms of expected adverse reactions to the IMPs and be given contact details for the study team. They will be advised to make contact with any ad hoc reports or queries.

The IDSMC will maintain oversight of incidence of adverse events when reviewing reports and will advise the TSC regarding any safety-related trends or concerns.

8.7 Maintenance of Blinding

Systems for SUSAR reporting should, as far as possible, maintain blinding of individual clinicians and of staff involved in the day-to-day running of the trial. Unblinding clinicians may be unavoidable if the information is necessary for the medical management of particular participants. The safety of participants in the trial always takes priority.
Cases that are considered possible SUSARs would have to be unblinded at NWORTH prior to reporting to the MHRA.

9 ETHICAL CONSIDERATIONS

9.1 Approval

The trial protocol, associated documentation, and all substantial amendments thereof will be submitted for review by a Research Ethics Committee (REC) with expertise in studies involving children. Approvals from the local health board will be obtained.

9.2 Considerations

The study will abide by the principles of the World Medical Association Declaration of Helsinki. We consider the specific ethical issues related to participation on this trial to be:

- Informed consent in the paediatric population
- Procedure for weaning off steroids and randomisation to placebo
- Post treatment management plan

9.3 Informed consent in the paediatric population

Informed consent is a process initiated prior to an individual agreeing to participate in a trial and continues throughout the individual's participation. In obtaining and documenting informed consent, the Investigator should comply with applicable regulation requirements and should adhere to GCP and the ethical principles that have their origin in the Declaration of Helsinki.

Proxy informed consent will be obtained prior to entry in to each part (1, 2 and 3) of the study.

Note that consent must be obtained from a person with parental responsibility defined by the Children's Act 1989 and the Chidren and Adoption Act 2002. The following are therefore acceptable

- Mother
- Father if married or divorced

- Unmarried father if named on the birth certificate only for children born after 01/12/2003
- Parental Responsibility agreement or court order

In order to provide information about the trial, parents (those with parental responsibility) will receive an ethically-approved parent information sheet which explains the details of the study including procedures, interventions and associated risks and benefits. Age or stage-of development participant information and assent forms will also be prepared so that children can provide their written assent where appropriate.

The Investigator or an appropriate member of the study team will then explain the research to the participants and answer any questions that may arise. Following an appropriate amount of time to consider the information provided, the parent will sign and personally date the proxy informed consent form. Assent will be obtained from the child to the appropriate level of their capacity using age and state of development information sheets and assent form. Both forms will be countersigned and dated by the Investigator or designee; a copy of the signed information and consent/assent forms will be provided to the participants and the original will be retained in the study notes.

The right of those with parents to refuse consent for the minor to participate in the trial without giving reasons must be respected, as must the rights of the minor to refuse assent.

The informed consent process should not cease once the informed consent form has been signed. The practise of giving information about the study to participants should be an ongoing process performed by all members of the research team to discuss the care of their infant in relation to the trial. This includes giving parents and children continuing opportunities to ask questions, discuss upcoming procedures, clarify any issues about the study and confirm their agreement to their ongoing participation in the study. Comprehension of the trial and its potential risks is enhanced by this 'continuous consent' process (Allmark and Mason, 2006) and thus a more informed consent is obtained.

This is particularly significant with the introduction of protocol amendments and the availability of important new information that may be relevant to the parent(s)/legal

guardian(s) willingness to continue participation in the study. In these circumstances it may be necessary to re-consent using amended information sheets and consent forms in order to continue their involvement in the study. We have established a process to re-consent participants prior to each part of the study in order to assure that they are as informed as possible.

9.4 Procedure for weaning off steroids

Our previous data indicates that a number of potential participants may be receiving inhaled treatment for control of respiratory symptoms. In order to include these participants in the treatment trial in Part 2, it will be necessary to wean those receiving inhaled corticosteroids in order to establish their baseline lung function and eligibility. The Trial Steering Committee believes subsequently randomising such children to placebo would be unethical and potentially harmful. To this end:

- Following screening, eligible children currently taking inhaled corticosteroids will be offered a consultation with a consultant respiratory paediatrician for a review of their medical history and further assessment by a research nurse if they meet any of the following criteria:
    - More than 3 courses of oral steroids in the past year, and/or
    - Hospital admission for an exacerbation of respiratory symptoms in the last year, and/or
    - Ever had intensive care admission for respiratory exacerbation and/or
    - FEV_1 <70% predicted, without associated symptoms and/or
    - FEV_1 <75% but >70% predicted, with associated symptoms and/or
    - Treatment with inhaled steroids >1000mcg budesonide equivalent per day
- If deemed safe to do so, a 4-week washout period prior to scheduling participation in part 2 of the study will be performed
    - The study team will maintain weekly contact with this group of children
    - In the event of a relapse of respiratory symptoms, the participant should seek medical assistance urgently (GP, Hospital depending on severity)

where appropriate treatment can be instituted, or contact the study team who will arrange urgent assessment either by the local general practitioner, consultant respiratory physician or local hospital paediatric department. In any case the study team should be contacted at the family's earliest convenience following the exacerbation
    - An appointment with the respiratory consultant will then be made at the earliest convenience to re-assess whether washout should continue and make a decision regarding timing of baseline assessments for part 2 including if randomisation is reasonable
- Children completing the steroid washout will constitute a separate treatment arm and will be randomised to one of the two active medications only (not placebo)
- The above process will be approved by the study Independent Scientific Committee in the review of this protocol

9.5 Post-treatment management plan

The Trial Steering Committee are aware that participants will be not be informed of their treatment allocation until after publication of the main trial results. Therefore, following participation of the treatment trial:

- Families will be encouraged to make an appointment with their GP to discuss possible treatment options where considered advisable by the study team
- A consultant respiratory paediatrician (member of TSC) will write to the GP with a suggested management plan based on the results of the pre-and-post treatment investigations and best evidence available at the time of writing
- The GP will make the decision on the appropriate treatment for their patient

9.6 Study discontinuation

Both those with parental responsibility and the minor remain free to withdraw at any time from the study treatment, data collection/follow-up or both without giving reasons. This right must be respected. Where possible, in agreement with participants, follow-up and data collection will continue following withdrawal from the intervention unless declined by the participants. Similarly, data from those withdrawn will be used in analysis unless consent for this is specifically withdrawn.

10 REGULATORY APPROVAL

This trial with obtain a Clinical Trials Authorisation (CTA) from the UK competent authority, the MHRA. The EudraCT number is **2015-003712-20**. All substantial amendments judged by the Sponsor to require regulatory opinion will be submitted for approval. Development Safety Update Reports will be submitted by the anniversary date of the CTA.

11 DATA COLLECTION AND TRIAL MONITORING

11.1 Risk Assessment

The RHINO study has undergone a risk assessment to ascertain the monitoring strategy required. In conducting this risk assessment, the contributors considered potential participants, organisational and study hazards, the likelihood of their occurrence and resulting impact should they occur.

The outcome of the risk assessment has been used in the finalisation of this protocol will be used in the development of the monitoring plan and statistical analysis plan.

11.2 Source data

The Case report form (CRF) will be considered the source data, and should be consistent and verifiable with the information recorded on the study database. Information regarding how the data is to be collected, stored, and transferred is included in the study-specific Data Management Plan.

11.3 Monitoring

A monitoring plan, based on the risk assessment, will be prepared prior to participant recruitment detailing the monitoring strategy for the trial. The plan will include procedures for day-to-day centralised monitoring, the requirements for source data verification, Investigator Site File (ISF) audit, and for identification of protocol deviations and serious breaches of protocol and/or GCP.

11.3.1 Direct access to data

In order to perform their role effectively, monitors and persons involved in quality assurance and inspection may need direct access to source data. Since this affects the participant's confidentiality, this fact is included on the Parent Information Sheet and Informed Consent Form.

11.3.2 Confidentiality

Individual participant medical information obtained as a result of this study is considered confidential and disclosure to third parties is prohibited with the exceptions noted below:

- Case report forms will be labelled with participant initials and unique trial screening and/or randomisation number.
- Medical information may be given to the participant's medical team and all appropriate medical personnel responsible for the participant's welfare.

NWORTH will preserve the confidentiality of participants taking part in the study and the Sponsor is registered as a Data Controller with the Information Commissioners Office.

11.3.3 Quality Assurance and Quality Control of Data

QA includes all the planned and systematic actions established to ensure the trial is performed and data generated, documented/recorded and reported in compliance with applicable regulatory requirements. QC includes the operational techniques and activities done within the QA system to verify that the requirements for quality of the trial-related activities are fulfilled. This trial has undergone a risk assessment, the outcome of which will develop the monitoring plan and the QC checks required. To this end:

- The Trial Manager/Coordinating Centre is to verify appropriate approvals are in place prior to initiation of the study and the relevant personnel have appropriate study-specific training and GCP training where applicable
- The Investigator will ensure that all members of the study team are qualified by training and experience to undertake any delegated duties, to be recorded on the 'Delegation of Authority and Signature Log'. Signed, dated CVs of the trial team will be stored in the Trial Master File.
- Oversight of the trial will be provided by the Independent Data and Safety Monitoring Board and Trial Steering Committee

11.4 Records Retention

The Investigator will make arrangements to store the essential trial documents, including the Trial Master File, for up to a maximum of 25 years or until the Sponsor informs that the documents are no longer to be retained.
In addition, the Investigator is responsible for archiving of all relevant source documents so that the trial data can be compared against source data after completion of the trial (e.g. in case of inspection from authorities).

The Investigator is required to ensure the continued storage of the documents, even if they, for example, leave the institution or retire before the end of required storage period. Delegation must be documented in writing.

12 INDEMNITY

Cover for harm as a result of the design or conduct of the trial has been arranged with the study Sponsor.

Cover for negligent harm is in place through the Clinical Negligence Scheme for Local Health Boards. If there is negligent harm during the study when the NHS body owes a duty of care to the person harmed, NHS indemnity covers NHS staff, medical academic staff with honorary contracts, and those conducting the trial. NHS indemnity does not offer no-fault compensation and is unable to agree in advance to pay compensation for non-negligent harm. Ex-gratia payments may be considered in the case of a claim.

For the purposes of the study Clinical Negligence is defined as:

"A breach of duty of care by members of the health care professions employed by NHS bodies or by others consequent on decisions or judgments made by members of those professions acting in their professional capacity in the course of their employment, and which are admitted as negligent by the employer or are determined as such through the legal process". (NHS Indemnity: Arrangements for Clinical Negligence Claims in the NHS (October 1996))

13 TRIAL COMMITTEES

Membership of the trial committees is listed on the supplementary document "RHiNO Trial Committee members".

13.1 Trial management Group (TMG)

The trial management group will be responsible for the day-to-day running and management of the trial and will meeting frequently during set up and subsequently on an agreed periodic basis once the trial is open to recruitment

13.2 Independent Scientific Committee

The Scientific Committee will comprise of experts in paediatric respiratory medicine; they will provide ethical and scientific opinion on the draft study protocol.

13.3 Independent Data and Safety Monitoring Committee (IDSMC)

The Independent Data and Safety Monitoring Committee will consist of an experienced clinicians and a statistician with experience in clinical trials involving children.

The committee will be responsible for reviewing and assessing recruitment, interim monitoring of safety and efficacy, and trial conduct. The IDSMC will first convene prior to the trial opening to recruitment but will have corresponded in order to approve the protocol prior to REC submission. The IDSMC will then define the frequency of subsequent meetings and the content of the reports to be presented at each meeting.

The IDSMC may recommend to the Trial Steering Committee that the trial be stopped or amended if sufficient evidence emerges that the trial intervention is clearly indicted or contra-indicated. Analyses will be reported to the IDSMC members who will consider the data in a clinical context accounting for other emerging evidence and overall clinical relevance.

The IDSMC will comply with a trial-specific charter which will be agreed and signed prior to the start of recruitment.

13.4 Trial Steering Committee (TSC)

Trial Steering Committee will consist of members of the TMG plus expert members and the parent representative. The role of the TSC is to provide overall supervision for the trial and provide advice. The ultimate decision concerning recommendations to the sponsor and the funder about the continuation of the trial lies with the TSC.

14 PUBLICATION

The results will be published as soon as possible after the close of the trial. Individuals must undertake not to submit any data for publication without the prior consent of the Investigator

The Uniform Requirements for Manuscripts Submitted to Biomedical Journals (http://www.icmje.org/) and the CONSORT guidelines will be respected. The ISRCTN allocated to this trial will be attached to any publications resulting from this trial.

BMJ guidance on authorship and contributorship (see http://bmj.com/advice/3.html) will be used to acknowledge the level and nature of contribution of key individuals in publications arising from the trial. The publication strategy shall lie under the jurisdiction of the Trial Steering Committee.

The protocol may be submitted for publication.

15 PROTOCOL AMENDMENTS

15.1 Current version

Final version 10 16/10/2018

15.2 Amendments

Final Version 9 to 10

Pg/section	Changes to text
5.1	typographical error - this should have read 100 consenting participants as controls
5.3	Clarification of number of weeks between visit 1 and 2, to ensure convenience for children and parents
5.4	Change to xenon as the hyperpolarised gas used in MRI<br>Addition of an MRI performed before and after a bronchodilator
6.1	Clarification of placebo canister supplier- GSK<br>Clarification that GSK placebo inhalers are CE marked<br>Clarification that a third batch of inhalers will be procured in late 2018
7.2.1	Clarification of FEV1 % required for pre-term born and term born control children.
7.2.2	Derivation of sample size calculations have been described in more detail

Final Version 8 to 9

Pg/section	Changes to text
5.1	Additional recruitment of potential participants
39	Addition of Adverse event to schedule of procedures for Part 3 MRI

Final version 7 to 8

Pg/section	Changes to text
11.2	Reference to data management plan

6.1	Change to the inhaler procurement<br>Change to the number of inhalers provided to participants<br>Revision of temperature storage requirements
7.1	Change to the inhaler procurement
6.3	Revision of temperature storage requirements
5.3.2	Corrected spelling errors

Final version 6 to 7

**Pg/section**	**Changes to text**
1	Change to number of research sites
Page 2	Addition of Statistician's signature
5.1	Additional research sites added
6.1	Clarification of IMP packaging and labelling procedure
7.1	Change of text in relation to nomenclature for consistency with IMP manufacture and randomisation

Final version 5 to 6

**Pg/section**	**Changes to text**
5.1	Additional recruitment strategy added- contact of participants outside of current RHiNO database
7.2.2	Typographical error: change to $p<0.05$
7.5	Clarification of data required for internal pilot of sample size assumptions

Final version 4 to 5

**Pg/section**	**Changes to text**
5.4	Addition of multiple breath washout (lung clearance index) to part 3 of study (LCI aspect removed from part 2)
8.2	Clarification of source of reference safety information
6.1	New source of placebo canister- Pharmaserve North West Ltd.<br>Removal of detail regarding use of masking device.<br>Change of manufacturer from Catalent to St Marys Pharmaceutical Unit.

Final version 3 to 4

Pg/section	Changes to text
6.1	Clarification of source of placebo canister
8.3	Addition of section "treatment withdrawal criteria". Discussion of potential inclusion of females of reproductive potential as requested by the MHRA
8.6	Addition of section "Nature and frequency of safety assessments" as requested by the MHRA

Final version 2 to 3

Pg/section	Changes to text
Throughout	Version control modified to reflect NWORTH procedures
Cover page	Addition of ISRCRN
Pg8	'Description of intervention' updated with details of new IMP treatment groups
Pg 9	Flowchart updated to include: Cardiovascular assessment, Helium dilution test and FOT test. Removal of CO2 transfer test
5.2.1	Clarification of restrictions prior to part 1 home visit
5.2.1	Reversibility test: Removal of the specific use of Ventolin brand salbutamol
5.3.1	Clarification of restrictions prior to part 2 lab visit 1
5.3.1	Clarification that the respiratory questionnaire will be repeated if part 1 visit 1 is >3 months after the initial questionnaire is completed
5.3.1	Addition of detail regarding cardiovascular assessment
5.3.1	Removal of CO transfer test
5.3.1	Addition of the FOT test
5.3.1	Addition of the inert gas washout test
5.3.1	Exercise challenge: Small clarifications to the procedure
5.3.1	Exercise challenge: Removal of the specific use of Ventolin brand salbutamol
5.3.1	Sputum induction: Addition of safety precaution- FEV1 to be within 90% of baseline before starting test

6	Change of treatment allocation to double-dummy design.<br>Inclusion of Serevent in the combination therapy group and removal of Seretide.
6.1	Inclusion of Serevent in the combination therapy group and removal of Seretide.<br>Explanation that placebo is identical to active drug<br>Detailed explanation of the blinding process included including description of the revised double-dummy design<br>Insertion of explanatory figure for double dummy design<br>Removal of the specific use of Ventolin brand salbutamol<br>Clarification of randomisation number formatting<br>Inclusion of new IMP manufacturer details (Catalent Pharma Solutions)<br>Revision of temperature storage requirements<br>Detail regarding storage of IMP outside of pharmacy added and monitoring procedure
6.3	Clarification that participants must take each inhaler, twice daily (AM and PM)<br>Clarification of temperature storage requirements<br>Clarification that local pharmacy will dispose of returns
6.5.1.1	Change of process from on-line system to envelopes for emergency unblinding procedure
6.8	Removal of the specific use of Ventolin brand salbutamol
6.9	Removal of the specific use of Ventolin brand salbutamol
7.2	Derivation of sample size calculations have been described in more detail
Table 1	Updated to reflect addition/removal of lung function tests

Final version 1.0 to 2.0

Pg/section	Changes to text
Pg 9	Flow chart updated to include QofL and Health Economic questionnaires
5.2.1	Information on accelerometry removed
5.2.1	Information on completion of CHU-9D questionnaire added

5.3.1	Detail on completion of health economic questionnaire and CHU-9D added
5.3.2	Detail added regarding collection of peak flow data
5.3.3	Information on accelerometry removed
6.1	Amended to include detail on placebo formulation
Pg 36	Table 1 scheduled of procedures updated

16 REFERENCES

Allmark P, Mason S. Improving the quality of consent to randomised controlled trials by using continuous consent and clinician training in the consent process. J Med Ethics 2006;32:439-43.

Asher MI, et al. International study of asthma and allergies in childhood (ISAAC): Rationale and methods. Eur Respir J 1995;8:483-491.

Bolton CE, Bush A, Hurst JR, Kotecha S, McGarvey L, Stocks J, Walshaw M. Are Early Life Factors Considered when Managing Respiratory Disease? A British Thoracic Society (BTS) Survey of Current Practice. Thorax 2012;67:1110.

Bolton, CE, Broekhuizen R, Ionescu AA, Nixon LS, Wouters E F, Shale DJ, Schols AM. Cellular protein breakdown and systemic inflammation are unaffected by pulmonary rehabilitation in COPD. Thorax 2007;62:109-14.

Boyd A, Golding J, Macleod J, Lawlor DA, Fraser A, Henderson J, Molloy L, Ness A, Ring S, Davey Smith G. Cohort Profile: The 'Children of the 90s'--the index offspring of the Avon Longitudinal Study of Parents and Children. International Journal of Epidemiology 2013;42:111-127.

Chakraborty M, McGreal EP, Kotecha S. Acute lung injury in neonates: mechanisms and management. Paed Resp Reviews 2010;11:162-170.

Diaz S[1], Casselbrant I, Piitulainen E, Pettersson G, Magnusson P, Peterson B, Wollmer P, Leander P, Ekberg O, Akeson P. Hyperpolarised 3HE apparent diffusion coefficient MRI of the lung: reproducibility and volume dependency in health volunteers and patients with emphysema. J Magn Reson Imaging 2008; 27:763-70.

Edwards, MO., Kotecha, SJ., Lowe, J., Richards, L., Watkins, WJ. & Kotecha, S. (2016). Management of Prematurity-Associated Wheeze and Its Association with Atopy. *PLoS ONE. 11* (5). e0155695.

Fawke J, Lum S, Kirkby J, Hennessy E, Marlow N, Rowell V, Thomas S, Stocks J. Lung function and respiratory symptoms at 11 years in children born extremely preterm: the EPICure study. American Journal of Respiratory and Critical Care Medicine 2010;182:237-45.

Goldenberg RL, Culhane JF, Iams JD, Romero R. Preterm Birth 1. Epidemiology and causes

of preterm birth. Lancet 2008;371:75-84.

Gough A, Spence D, Linden, M, Halliday HL, Mcgarvey LP. General and respiratory health outcomes in adult survivors of bronchopulmonary dysplasia: a systematic review. Chest 2012;141:1554-67.

Joshi S, Kotecha S. Lung Growth and Development. Ear Hum Develop 2007;83:789-94.

Joshi S, Powell T, Watkins W J, Drayton M, Williams EM, Kotecha S. Exercise-Induced Bronchoconstriction in School-Aged Children Who Had Chronic Lung Disease in Infancy. The Journal of paediatrics 2013;162:813-818.

Korhonen P, Laitinen J, Hyodynmaa E, Tammela O. Respiratory outcome in school-aged, very-low-birth-weight children in the surfactant era. Acta Paediatrica 2004;93:316-21.

Kotecha SJ, Dunstan F, Kotecha S. Long term outcomes of late preterm birth. Seminars in Fetal and Neonatal Medicine 2012a;17:77-81.

Kotecha SJ, Watkins WJ, Paranjothy S, Dunstan FD, Henderson AJ, Kotecha S. Effect of late preterm birth on longitudinal lung spirometry in school age children and adolescents. Thorax 2012b;67:54-61.

Kotecha S, Dunstan F, Adappa R, Paranjothy S, Hopkins J, Rolfe K, Watkins J. All Wales Perinatal Survey - Annual Report 2011. Cardiff 2012: Cardiff University.

Kotecha SJ, Edwards MO, Watkins WJ, Lowe J, Henderson AJ, Kotecha S. Effect of inhaled drug treatment and on %FEV_1 in preterm-born subjects - a systematic review. Neonatology 2015;107:231-240.

Lowe J, Watkins WJ, Edwards MO, Kotecha SJ, Henderson AJ, Kotecha S. Levels of physical activity in preterm born school-age children. Journal of Pediatrics 2015 Apr;166:877-8

ONS Office for National Statistics 2012: Births and Deaths in England and Wales (Provisional), 2011. Statistical Bulletin.

Pelkonen, AS, Hakulinen AL, Turpeinen M. Bronchial lability and responsiveness in school children born very preterm. American Journal of Respiratory and Critical Care Medicine 1997:156;1178-84.

Pelkonen, AS, Hakulinen AL, Hallman M, Turpeinen M. Effect of inhaled budesonide therapy

on the lung function in school children born preterm. Respiratory Medicine 2001;95:565-70

Saigal S, Doyle LW. An overview of mortality and sequelae of preterm birth from infancy to adulthood. Lancet 2008;371:261-9.

Teig N, Allali M, Rieger C, Hamelmann E. Inflammatory markers in induced sputum of school aged children born before 32 completed weeks of gestation. Journal of Pediatrics 2012;161:1085-90

The Consortium on Safe Labour. Respiratory Morbidity in Late Preterm Births. JAMA 2010;304:419-425.

North Wales Organisation for Randomised Trials in Health & Social Care
Sefydliad Hap-Dreialon Iechyd a Gofal Cymdeithasol Gogledd Cymru

PRIFYSGOL BANGOR UNIVERSITY

STATISTICAL ANALYSIS PLAN

FOR RHiNO

PART 2 - RCT

Date: 06/08/2019

Version: 2

North Wales Organisation for Randomised Trials in Health (NWORTH)
Bangor University, College of Health & Behavioural Sciences,
Institute of Medical & Social Care Research, Y Wern, Normal Site, Holyhead Road, Bangor, Gwynedd LL57 2PZ
Telephone: 01248 388095 Email: nworth@bangor.ac.uk Website: http://nworth-ctu.bangor.ac.uk/

Statistical analysis plan (SAP) for the Respiratory Health in Neonatal Outcomes (RHiNO) trial: a randomised, double blind, placebo-controlled trial of inhaled treatment to establish the mechanisms of prematurity-associated airway obstruction and inflammation.

The analysis for the RHiNO trial has been split over three different SAPs with this being the second, representing the RCT portion of the study.

EudraCT: 2015-003712-20

This document has been written based on information contained in the study protocol version 10, dated 16 10 2018.

Chief Investigator:	Prof Sailesh Kotecha
Co-Investigators:	Prof John Henderson
	Dr Iolo Doull
	Dr Mark Williams
	Dr Zoë Hoare
	Prof Jim Wild
Trial Manager	Alison Jenkins
Data Manager	Alison Jenkins
Trial Statistician	Nia Goulden

Nia Goulden	N Goulden 03/09/2019
**Author's Name**	**Date and Signature**
Zoë Hoare	ZHoare 3/9/2019
**Second Statistical Advisor**	**Date and Signature**
Sailesh Kotecha	[illegible] 30/8/2019
**Chief Investigator**	**Date and Signature**

Document History

Updated version no.	Effective date	Authorship	Section changed	Summary of changes
1	17/11/2017	N. Goulden	New	
2	03/09/2019	N. Goulden	1, 3, 5	Update trial staff, clarify that percentage predicted FEV_1 will be analysed, Update to data quality and assumptions, Updated interim analysis consistent with protocol, Additional information to clarify analysis, change to assessing for 10% absolute difference consistent with updated protocol, not 12% relative difference

Acronyms and definition of terms

Acronym	Meaning
ALSPAC	Avon Longitudinal Study of Parents And Children
ANCOVA	Analysis of Covariance
ANOVA	Analysis of Variance
BMI	Body Mass Index
CEAC	Cost Effectiveness Acceptability Curves
CHU-9D	Child Health Utility – nine dimensions
CO_2	Carbon dioxide
CONSORT	Consolidation Standards Of Reporting Trials
CTIMP	Clinical Trial of an Investigational Medicinal Product
EIB	Exercise Induced Bronchoconstriction
ERV	Energy Recovery Ventilation
FeNO	Fractional exhaled Nitric Oxide
FEV_1	Forced Expiratory Flow in One-Second
FRC	Functional Residual Capacity
FVC	Forced Vital Capacity

G_{aw}	Airway Conductance
IDSMC	Independent Data Safety Monitoring Committee
MFEF	Maximal Forces Expiratory Flow
NICE	National Institute for health and Care Excellence
NWORTH	North Wales Organisation for Randomised Trials in Health
O_2	Oxygen
PEF	Peak Expiratory Flow
QC	Quality Control
RANOPS	Respiratory and Neurological Outcomes in Pre Term Children
R_{aw}	Airway Resistance
RCT	Randomised Controlled Trial
RER	Respiratory Exchange Ratio
RHiNO	Respiratory Health Outcomes in Neonatal
RV	Residual Volume
SAP	Statistical Analysis Plan
SOP	Standard Operating Procedure
TLC	Total Lung Capacity
TMF	Trial Master File
TMG	Trial Management Group
TSC	Trial Steering Committee

Table of Contents

Acronyms and definition of terms 3
1. Statistical analysis plan authorship 6
2. Introduction 6
2.1 Background and Rationale 6
2.2 Trial Objectives 6
2.3 Trial Design 8
2.4 CONSORT Diagram 8
3. Statistical Principles 8
3.1 Sample size justification 8
3.2 Randomisation 9
3.3 Levels of confidence and p-values 9
3.4 Protocol Deviations and Violations 9
3.5 Missing Data 10
3.6 Outliers 10
3.7 Assumption Checking 10
3.8 Analysis Syntax 11
3.9 Data Quality Assessment 12
4. Data 12
4.1 Outcome measures 12
4.2 Definitions and calculations of outcome measures 13
4.3 Safety data 13
Unblinding 13
5. Statistical analyses 14
5.1 Analysis Time Frame 14
Interim Analysis 14
5.2 CONSORT Analysis 14
5.3 Descriptive statistics 15
5.4 Phenotype analysis 15
Bronchoconstriction after exercise 16
5.5 Intervention analysis (Primary Outcome) 17
Intervention Analysis (Secondary Outcomes) 18
5.6 Drug Responses 18
5.7 Subgroup analyses 19
5.8 Sensitivity analyses 19
6. Health economics analysis 20
6.1 Aims 20
6.2 Analysis 20
6.3 Sensitivity analyses 21
7. Software 21
8. References 21
9. Appendices 22

1. Statistical analysis plan authorship

The analysis plan has been authored by Nia Goulden, Trial Statistician as of 18th May 2017. The majority of the writing has been completed by Nicola Totton, Trial Statistician until 17th May 2017. There has also been input from Zoë Hoare (Principal Statistician), John Lowe (Trial and Data Manager until 11/01/2017), Alison Jenkins (Trial and Data Manager from 1th December 2017) and Sailesh Kotecha (Chief Investigator). The health economics (section 6) has been authored by Lucy Bryning and Carys Jones with input from Rhiannon Tudor Edwards. The draft plan will be circulated to the IDSMC for comment prior to being signed off. The baseline analysis will be completed by Michael Cousins as part of his PhD and will be overseen by Zoë Hoare and Nia Goulden. Michael has had input into this analysis plan and has approved the final version. The remaining analysis, looking at the drug intervention, will be completed by Nia Goulden.

2. Introduction

2.1 Background and Rationale

Survival of pre-term infants has markedly improved with modern developments in neonatal care, however, it is recognised that these infants may still have adverse long-term respiratory outcomes into childhood and beyond[1]. Although repeated studies show that such children have decreased lung function and increased respiratory symptoms[2], it is unclear what the exact mechanisms of disease are and how they respond to treatment.

This SAP relates to the RCT section of RHiNO study, which consists of two lab visits for the children, 12 weeks apart.

2.2 Trial Objectives

The main objectives of the RCT part of the RHiNO study are to:

1. Compare baseline demographics, measures of lung function (including static and dynamic measures), allergic status (skin prick testing) and measures of inflammation (eNO) between the pre-term born children with airway

obstruction, pre-term born control children and term born control children.

2. Compare pre and post exercise lung function measures to assess bronchoconstriction at baseline, and additionally after post exercise bronchodilator (salbutamol), between pre-term born children with airway obstruction, pre-term born control children and term born control children. This will be achieved by comparing percentage predicted FEV_1, and other measures of obstructive airway disease, pre and 15-20 minutes post exercise, in pre-term born children with airway obstruction, pre-term born control children and term born control children.
3. Assess if a 12-week treatment of an anti-inflammatory (Flixotide) or an anti-inflammatory coupled with a bronchodilator (Flixotide/Serevent combination) improves airway obstruction when compared to placebo in pre-term born children with obstructive airway disease. This will be achieved by comparing the difference in percentage predicted FEV_1 before and after treatment, in the three treatment groups of pre-term born children with obstructive airway disease, to assess whether there has been an improvement in airway obstruction.
4. Assess if a 12-week treatment of an anti-inflammatory (Flixotide) or an anti-inflammatory coupled with a bronchodilator (Flixotide/Serevent combination) modifies the underlying measures of obstructive airway disease when compared to placebo in pre-term born children with obstructive airway disease. This will be achieved by an exploratory analysis, comparing the difference in measures of obstructive airway disease before and after treatment, in the three treatment groups of pre-term born children with obstructive airway disease, to assess whether there has been any modification to the measures of obstructive airway disease.
5. Compare pre and post exercise lung function measures to assess bronchoconstriction at baseline, and additionally after post exercise bronchodilator (salbutamol), between the three treatment groups of pre-term born children with airway obstruction. This will be achieved by comparing percentage predicted FEV_1, and other measures of obstructive airway disease, pre and 15-20 minutes post exercise in the three treatment groups. These

measurements will be assessed both before and after treatment.

6. Evaluate any factors that could be considered a predictor to drug responses. This will be achieved by defining each child as having a positive response, negative response or no response to treatment. A regression analysis will evaluate which of the measures of obstructive airway disease and demographic information predict the outcome.

2.3 Trial Design

This part of the study is designed as a single-centre, double blind, double-dummy, randomised, placebo controlled trial.

2.4 CONSORT Diagram

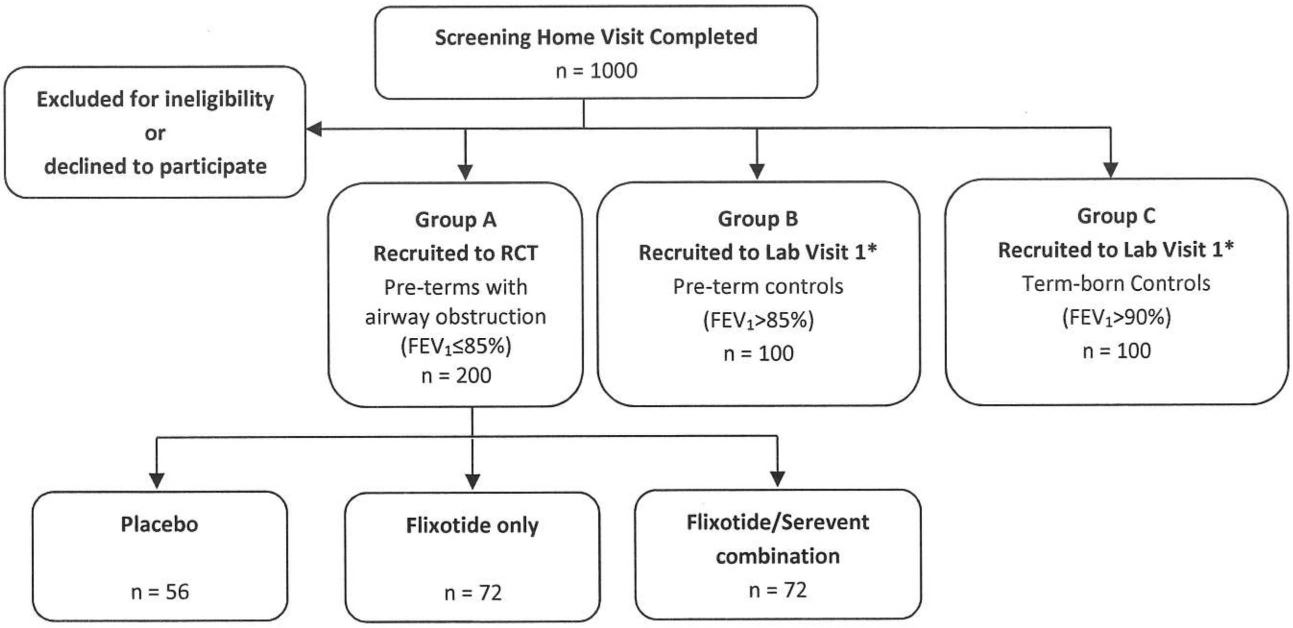

* Those children recruited as pre-term or term-born control complete only lab visit 1.

3. Statistical Principles

3.1 Sample size justification

Using a one-way ANOVA to show a 10% absolute difference with a conservative standard deviation of 10, α=0.05 and power=80%, the study is recommended to have 53 completers. Allowing for 20% attrition leaves 67 participants.

3.2 Randomisation

Children will be randomised to one of three groups within the RCT. The RANOPS study[3] found that 13.3% of very pre-term children were using corticosteroid inhaler medication and therefore 86.7% are anticipated to be steroid-free. Those **not** currently on steroids will be allocated to either: placebo, Flixotide or Flixotide/Serevent combination, whereas those **already using** corticosteroids will be allocated to Flixotide or Flixotide/Serevent combination. This gives an approximate final allocation ratio of 1:1.3:1.3 of placebo: Flixotide: Flixotide/Serevent combination.

Because the sizes of the groups are unequal it will not be possible to maintain complete blinding to allocations. Since the placebo group is smaller than the flixotide and flixotide/serevent combination groups, it will be possible to identify the placebo group.

3.3 Levels of confidence and p-values

All applicable statistical tests will be two-sided and will performed using a 5% significance level and all confidence intervals presented will be 95% and two-sided.

3.4 Protocol Deviations and Violations

A protocol deviation is an unintended failure to adhere to the protocol; examples include errors in applying inclusion/exclusion criteria or missed follow-up visits due to error. A protocol violation is an intended failure to adhere to the protocol such as wrong treatment being prescribed or administered or incorrect data being collected and documented. A table containing any protocol deviations or violations will be summarised within the final analysis, this will include details on the level of deviation as described in the RHiNO Monitoring Plan.

3.5 Missing Data

Every effort will be taken to minimise missing data by effective training of the research staff that will be collecting the information. Even so, a check will be completed to evaluate whether any missing data is missing completely at random. If this is the case then no further action will be taken to account for the missing items. It is possible that the lab visits may not be fully completed by some children and therefore the data will not be missing at random. In this case, an assessment will be made to evaluate any predictors of non-completers; if found, these will be included into analysis models as covariates. To deal with the missing data, multiple imputation will be used, where appropriate.

3.6 Outliers

Outliers identified from the descriptive analyses will be examined by rechecking the data. No outliers will be discarded if they can be verified or are within range. If any outliers are dropped from the dataset it will be reported and full reasoning given.

3.7 Assumption Checking

A check of the continuous variables within this part of the study will be completed to ensure that they are normally distributed, in order to confirm they are being treated in the correct way.

For the regression we will check that the residuals from the model are normally distributed. A scatterplot will be produced of the standardised residuals against the predicted values to test for homoscedasticity. There will also be a check that there is a linear relationship between the dependent variable and each of the independent variables, and that there is no multicolinearity.

For ANOVA tests we will check the assumptions required by the test. We will check that the residuals of the models are approximately normally distributed and that there is homogeneity of variance. A scatterplot will be produced of the standardised residuals against the predicted values to test for homoscedasticity. In the event of

these assumptions being violated it will be necessary to transform the data in order to satisfy the assumptions of the test. In the event of the residuals being skewed to the right, or the variance increasing with an increasing independent variable, a transformation using a lower power, such as square root, cube root or a log transformation will be used. In the event of the residuals being skewed to the left, or variance decreasing with increasing independent variable, a transformation using a higher power, such as square root or cube root transformation will be used. This should also address any violations of non-normality of data or homogeneity of variance.

For the ANCOVA tests, in addition to the checks performed for ANOVAs, we will need to produce scatterplots of the covariates against the dependent variables for each level of the independent variables. This should show a linear relationship between the covariate and dependent variable. The lines of best fit should be parallel, so that there is no interaction between the covariate and the independent variable. In the event of this assumption being violated it will be necessary to add the interaction term of the covariate and dependent variable to the ANCOVA.

It is likely that some of the measures being collected will not be normally distributed. In this case we will consider using the z-score of the measure if possible. Alternatively we will consider using a generalised linear model with appropriate distribution family and link function specified, which will be determined from examining the final dataset. The most appropriate method will be determined from examining the final data.

3.8 Analysis Syntax

All analysis will be completed using syntax/code within the program being used to ensure reproducibility of the results. This will be completed in accordance with 5.WI.03 Analysis code verification, which stipulates that for Clinical Trials of Investigational Medicinal Products (CTIMPs), the analysis code will be checked by a statistical verifier. The analysis presented here is considered as simple and so the procedure for verifying simple analysis can be followed.

3.9 Data Quality Assessment

As at screening, it is thought that there may be some inconsistencies with the spirometry results, although this is expected to be considerably less than at screening. The lung function data from the device will be quality checked by Mark Williams. These checks will be completed in line with the European Respiratory Society (ERS)/American Thoracic Society (ATS) guidelines and any concerns marked on the monitoring spreadsheet.

Data are all assessed for quality control, and only the quality controlled data will be used.

4. Data

For full details on the data collection, flow and storage please refer to the current version of the RHiNO Data Management Plan.

4.1 Outcome measures

The data to be collected primarily relate to measures of lung function. Further data are to be collected on quality of life and health economic outcomes.

Outcomes will be assessed in two ways:

a) Phenotype analysis – this is to evaluate the differences between children born prematurely with reduced lung function and both pre-term born and term-born controls (i.e. normal lung function). These comparisons will be completed using lab visit 1 data as this is the only time point that the control children will be attending. These are represented as group A, B and C respectively in section 2.4 above.

b) Intervention analysis – this is to compare the differences between those children that have been recruited into the RCT (and are therefore a subset of the previous population). These children are then further split into the 12 week treatment that they received (Placebo, Flixotide only, Flixotide/Serevent combination). Data will be analysed using an intention to treat approach.

4.2 Definitions and calculations of outcome measures

See Table in appendix 1 for full details.

4.3 Safety data

Any safety events from the interventional trial will be reported with details of severity, cause and outcome included. The number and percentage of patients that have suffered an adverse event will also be presented descriptively. No formal statistical testing will be undertaken on this data.

Unblinding

Any emergency unblinding that is required during the trial for specific participants due to adverse events will be completed in accordance with trial specific RHiNO SOP on the Unblinding Procedure. Should the IDSMC request to be unblinded to the group allocations, they will follow the procedure for emergency unblinding at NWORTH to find the treatment allocations. The final unblinding for the results will only be done after all analysis, as stipulated in this plan, has been completed. The unblinding form (found in the Appendix of SOP 5.03 Randomisation systems) will be completed by the Trial Statistician and handed to the NWORTH IT team who will then provide the group details to the team within the next TMG or TSC. This will be done by creating a sealed envelope with the group allocations inside to be open by the Chair. This unblinding will be included in the minutes of the meeting and the unblinding form completed and stored in the TMF.

5. Statistical analyses

5.1 Analysis Time Frame

TASK	EXPECTED DATE
First participant completes lab visit 1	June/July 2017
**FINAL PARTICIPANT COMPLETES LAB VISIT 1**	August 2019
First participant completes lab visit 2	2017
**FINAL PARTICIPANT COMPLETES LAB VISIT 2**	November 2019
Data cleaning completed	December 2019
Analysis completed	March 2020

Interim Analysis

An interim analysis is planned for 6 months after the first randomisation (or with a minimum of 50 participants enrolled) to the trial. An estimation of the standard deviation that was used within the sample size calculation (baseline percentage predicted FEV_1 at lab visit 1) will be completed to ensure it is within a sensible range. This analysis will be completed in a blinded fashion and will use data from all participants that have been randomised into the RCT. If the standard deviation is smaller than that used in the sample size calculation (15), then no further action will be taken and the trial will continue to recruit the initially proposed number of children. If the standard deviation is larger than expected, the sample size will be re-calculated and the information provided to the IDSMC and to the TSC to decide if the recruitment target should be increased.

5.2 CONSORT Analysis

The analysis will consider the points from the CONSORT checklist[4] to ensure that all topics are being covered. Values for eligibility rates, recruitment rates and attrition rates will be reported using the CONSORT data collected within the study.

Furthermore, details on reasons for ineligibility and non-recruitment will be reported within a table along with their related participant frequencies and percentages. Information on withdrawals and non-respondents will be presented including reasons where applicable and time points during the trial.

5.3 Descriptive statistics

Descriptive statistics of the data will be presented, all continuous measures will be reported with mean values and standard deviations and categorical variables presented with counts and related percentages. If data are not normally distributed then medians and interquartile ranges will be reported.

5.4 Phenotype analysis

One of the aims of the project is to evaluate the phenotype and characteristics of the children who are pre-term born with reduced lung function (Group A) against pre-term born children with normal lung function (Group B) and term-born children with normal lung function (Group C), these groups are identified in Figure 1.

This analysis will be exploratory and completed on data from lab visit 1, as this is the only lab visit that the control groups will attend. Differences between these groups will be assessed on a number of characteristics (described along with the related variables below) and will be analysed using an ANOVA model and post-hoc tests as applicable. The variables of interest are:

- Role of atopy; assessed as a positive response to skin prick test (wheal size greater than 3mm for one or more allergens);
- Eosinophilic airway inflammation; assessed using fraction of exhaled nitric oxide (FeNO)
- Airway inflammation; assessed using the proportion of cells, excluding epithelial cells, firstly neutrophils and secondly eosinophils. Additionally, concentration of inflammatory markers in sputum supernatant (IL-8) will be compared between the three groups at baseline.

- Oxidant injury; assessed using biomarkers found in exhaled breath condensate (8-isoprostane);
- Cardiovascular; assessed using systolic and diastolic blood pressure, mean arterial pressure, central augmentation index and pulse wave velocity;
- Anthropometric; assessed using weight, height, body mass index (BMI), fat mass, fat-free mass and muscle mass;
- Lung function (flow and volume/resistance); assessed using percentage predicted FEV_1, FEV_1/FVC ratio, PEF, $FEF_{25-75\%}$, FRC_{pleth}, FRC_{He}, TLC, ERV, RV, RV/TLC, R_{aw}, G_{aw}. The R_{aw} and G_{aw} values will be corrected for lung volume.

Bronchoconstriction after exercise

The following variables will be assessed in the same manner as those in the phenotype analysis described above[5]:

- Observed decrease in percentage predicted FEV_1 from pre- to post-exercise, this will be displayed graphically;
- Proportion with exercise induced bronchoconstriction (EIB); maximum absolute decrease in percentage predicted FEV_1 greater than 10 percent from pre- to post-exercise;
- Reversibility; defined as an absolute improvement of greater than 10 percent predicted FEV_1 from 15-20 min post exercise measure to post-salbutamol percentage predicted FEV_1;
- Proportion with absolute improvement of greater than 10 percent predicted FEV_1 from 15-20 min post exercise measure to post-salbutamol percentage predicted FEV_1;
- Exercise capacity; assessed using max respiratory rate, relative workload, O2 uptake/kg, CO2 production, minute ventilation, RER, breathing reserve max.

Amendments will be made for multiple comparisons at this stage of the analysis, as described below.

5.5 Intervention analysis (Primary Outcome)

The main analysis of the RHiNO study is to evaluate the effects on lung function of the two drug interventions (Flixotide and Flixotide/Serevent combination) compared with placebo. The primary outcome is percentage predicted FEV_1 of the child which is assessed at baseline (lab visit 1) and again after the 12-week intervention period (lab visit 2). The protocol states that the primary outcome is the difference in percentage predicted FEV_1 after 12 weeks of therapy between the active groups and placebo. This was based on an intention to utilise a difference of difference approach, however this will not now be used. An ANCOVA model will assess any differences between all three groups on the percentage predicted FEV_1 at lab visit 2, therefore FEV_1 at both time points is still being analysed within the model. Covariates will be included in the model relating to: the participants' percentage predicted FEV_1 from lab visit 1, whether they were already taking steroids (stratification variable), and any variables found from the screening analysis that have been shown to be a predictor for lung function will be considered for inclusion in the model. The groups will be randomly allocated (Flixotide, Flixotide/Serevent combination or placebo) and the primary analysis will be based on intention-to-treat.

It is likely that some of the measures being collected will not be normally distributed. In this case we will consider using the z-score of the measure if possible. Alternatively we will consider using a generalised linear model with appropriate distribution family and link function specified, which will be determined from examining the final dataset. The most appropriate method will be determined from examining the final data.

Post-hoc t-tests will be used where applicable. This will comprise comparisons of both interventions with placebo, and a comparison of both interventions. The placebo group will have fewer participants than the intervention groups, therefore it will be possible to identify the placebo group for this analysis. The threshold of significance of the t-tests will be amended using a Games Howell correction to adjust for multiple comparisons.

Intervention Analysis (Secondary Outcomes)

Similar models to that described above will be applied to the following secondary outcome variables that are being collected at the two lab visits:

- Lung function (flow and volume/resistance); assessed using percentage predicted FEV_1, FEV_1/FVC ratio, PEF, MFEF, FRC_{pleth}, FRC_{He}, TLC, ERV, RV, RV/TLC, R_{aw}, G_{aw};
- Inflammation and oxidant injury; assessed using fraction of exhaled nitric oxide (FeNO) and concentrations of biomarkers found in exhaled breath condensate (8-isoprostane);
- Exercise induced bronchoconstriction (EIB); defined as a absolute decline in percentage predicted FEV_1 greater than 10 percent predicted @15-20 mins post exercise. This is assessed both before and after treatment;
- Reversibility; defined as an absolute improvement of greater than 10 percent predicted FEV_1 from 15-20 min post exercise measure to post-salbutamol percentage predicted FEV_1. This is assessed both before and after treatment.

5.6 Drug Responses

After the RCT has finished, it will be determined for each of the children on an active treatment (i.e. Flixotide or Flixotide/Serevent combination) whether they responded to the drug or not. Children will be assigned to having either a positive response, no response or a negative response to the drug and this variable will be added to the database. This will be decided based on an absolute change in percentage predicted FEV_1 greater than or equal to 10 percent. Children who have an absolute increase of greater than or equal to 10 percent will be classified as a positive response, an absolute decrease of greater than or equal to 10 percent will be classified as a negative response and an absolute change of less than 10 percent will be classified as no response.

A regression will be completed using this variable relating to drug responses as the dependent variable. Independent variables to be initially included in the model will be: assigned treatment, whether they were previously taking steroids and any

variables which were determined to affect lung function from the screening analysis model. Multiple direction modelling will be used to create the final regression model. Interactions will be identified where there is theoretical evidence to suggest relevance.

5.7 Subgroup analyses

There will be a subgroup analysis of the baseline data. This analysis may be performed earlier than the full analysis including the follow-up data. An analysis of variance (ANOVA) or analysis of covariance (ANCOVA) will be used to compare the measures collected between the pre-terms with airway obstruction, pre-terms with no airway obstruction and healthy term-born children. Post hoc tests and correlations will also be conducted. Additionally a regression model will determine which parameters have an effect on other data, such as the spirometry and body plethysmography data.

5.8 Sensitivity analyses

Sensitivity analysis will be completed on the QC data as described in section 3.9.

There is likely to be a seasonal effect for some of the variables i.e. they will be different in the children at different times of year. We will include a variable to code for the season when the child attended the laboratory visit.

The overall treatment compliance will be reported for the three groups. Compliance will be assessed on an intention to treat basis, meaning that compliance will be reported according to the group that the child was randomised to.

Any mis-allocations of treatment will be assessed. If there are more than 5% mis-allocations, then a per protocol analysis will be conducted, in order to assess the effect of the treatment that was actually received. The intention to treat analysis will analyse the data using the groups that children were randomised to. The per protocol analysis will analyse the data using the group that represents what was received by the child.

Additionally, sensitivity analysis is planned on the health economics data as described in section 6.3.

6. Health economics analysis

6.1 Aims

The aim of the study is to investigate the use of traditional health economic measures to assess the cost-effectiveness of the inhaled treatment of prematurity-associated airway obstruction and inflammation.

Specifically the pilot economic evaluation will assess:

1. Using the CHU-9D[6] as the primary effectiveness outcome, what is the incremental cost-effectiveness of the combination inhaled treatment (Flixotide/Serevent combination) compared to the placebo condition?
2. Using the CHU-9D as the primary effectiveness outcome, what is the incremental cost-effectiveness of the single inhaled treatment (Flixotide) compared to the placebo condition?
3. Using the CHU-9D as the primary effectiveness outcome, what is the incremental cost-effectiveness of the combination inhaled treatment (Flixotide/Serevent combination) compared to the single inhaled treatment (Flixotide)?
4. What are the patterns of health care service use and associated costs for the study participants?
5. What are the patterns of school absenteeism and associated costs for the study participants?

6.2 Analysis

We propose a cost consequence analysis with embedded pilot cost-utility analysis to map out a whole range of outcomes including school attendance, time off work for parents and carers, quality of life measures (CHU-9D) and physiological outcomes.

If appropriate, we will present using bootstrapping to produce cost-effectiveness planes, and cost-effectiveness acceptability curves (CEAC), to healthcare policymakers

and local commissioners the probability that the intervention is cost-effective at different payer thresholds.

We will bootstrap (5,000 replications) differences in cost and outcomes to produce a 95% confidence interval around these differences.

6.3 Sensitivity analyses

Sensitivity analyses will be conducted to vary the costs of variables (e.g. the cost of the inhaler) in accordance with NICE guidelines[7].

7. Software

All quantitative analysis will be completed using SPSS v25 and R version 3.5.1.

8. References

1. Bolton CE, Bush A, Hurst JR, et al. Are early life factors considered when managing respiratory disease? A British Thoracic Society survey of current practice. *Thorax*. 2012;67(12):1110. doi:10.1136/thoraxjnl-2012-202637.
2. Fawke J, Lum S, Kirkby J, et al. Lung function and respiratory symptoms at 11 years in children born extremely preterm: The EPICure study. *Am J Respir Crit Care Med*. 2010;182(2):237-245. doi:10.1164/rccm.200912-1806OC.
3. Edwards MO, Kotecha SJ, Lowe J, Richards L, Watkins WJ, Kotecha S. Management of Prematurity-Associated Wheeze and Its Association with Atopy. *PLoS One*. 2016;11(5). doi:10.1371/journal.pone.0155695.
4. Moher D, Hopewell S, Schulz KF, et al. CONSORT 2010 explanation and elaboration: Updated guidelines for reporting parallel group randomised trials. *Int J Surg*. 2012;10(1):28-55. doi:10.1016/j.ijsu.2011.10.001.
5. Joshi S, Powell T, Watkins WJ, Drayton M, Williams EM, Kotecha S. Exercise-induced bronchoconstriction in school-aged children who had chronic lung disease in infancy. *J Pediatr*. 2013;162(4):813-818.e1. doi:10.1016/j.jpeds.2012.09.040.
6. Stevens K. Assessing the performance of a new generic measure of health-related quality of life for children and refining it for use in health state valuation. *Appl Health Econ Health Policy*. 2011;9(3):157-169. http://www.embase.com/search/results?subaction=viewrecord&from=export&id=L361646560%5Cnhttp://dx.doi.org/10.2165/11587350-000000000-00000.
7. Andronis L, Barton P, Bryan S. Sensitivity analysis in economic evaluation: An audit of NICE current practice and a review of its use and value in decision-making. *Health Technol Assess (Rockv)*. 2009;13(29). doi:10.3310/hta13290.

Referenced documents:

1. RHiNO Monitoring Plan
2. RHiNO Data Management Plan

9. Appendices

Appendix 1 – Validated outcome measures summary table

Outcome measure	Definition	Scoring	Subscales	Missing value rules	Thresholds
CHU9D	A paediatric generic preference based measure of HRQoL.<br><br>A 9 item HRQoL scale looking at how the respondent feels today.	Each of nine items has a response scored 1-5, with 1 representing perfect health and 5 representing worst health.<br><br>Final scores will be calculated using the preference weightings (UK and Australian) giving a utility index value anchored between 0 – 1.	No subscales.	No missing value rules.	No thresholds defined.
