# Supplementary material for: Inhaled Corticosteroids Alone and in Combination With Long-Acting β2 Receptor Agonists to Treat Reduced Lung Function in Preterm-Born Children: A Randomized Clinical Trial
Source: JAMA Pediatr. 2021 Dec 13;176(2):1–9. doi: 10.1001/jamapediatrics.2021.5111 (PMC8669602; doi:10.1001/jamapediatrics.2021.5111)
Supplement: Supplement 2. — eMethods 1. Physiologic Testing eMethods 2. Inhaler Intervention, Randomization, and Statistical Methods eTable 1. Randomized Groups eTable 2. Comparisons Between Attenders and Nonattenders eTable 3. Reasons for Withdrawal eTable 4. Results of ANCOVA of Spirometry Measures eTable 5. Sensitivity Analyses Showing ANCOVA Results of Spirometry Measures of Children Who Were Corticosteroid Naive at Randomization eTable 6. Spirometry at Baseline, Postexercise and After Postexercise Bronchodilator eFigure. %FVC and FEV1/FVC Ratio at Baseline, After Exercise, and After Postexercise Bronchodilator eReferences [file jamapediatr-e215111-s002.pdf]

Supplementary Online Content

Goulden N, Cousins M, Hart K, et al. Inhaled corticosteroids alone and in combination with long-acting β_2 receptor agonists to treat reduced lung function in preterm-born children: a randomized clinical trial. *JAMA Pediatr*. Published online December 13, 2021. doi:10.1001/jamapediatrics.2021.5111

eMethods 1. Physiologic Testing

eMethods 2. Inhaler Intervention, Randomization, and Statistical Methods

eTable 1. Randomized Groups

eTable 2. Comparisons Between Attenders and Nonattenders

eTable 3. Reasons for Withdrawal

eTable 4. Results of ANCOVA of Spirometry Measures

eTable 5. Sensitivity Analyses Showing ANCOVA Results of Spirometry Measures of Children Who Were Corticosteroid Naive at Randomization

eTable 6. Spirometry at Baseline, Postexercise and After Postexercise Bronchodilator

eFigure. %FVC and FEV_1/FVC Ratio at Baseline, After Exercise, and After Postexercise Bronchodilator

eReferences

This supplementary material has been provided by the authors to give readers additional information about their work.

eMethods 1. Physiologic Testing

- *Fractional exhaled Nitric Oxide (FE_{NO})*

FE_{NO} was performed using an exhaled nitric oxide analyser (NIOX VERO, Circassia, UK). Children were instructed in its use prior to performing test. The device required a warm-up period after being switched on, following which a warning would be issued if the sensor or breathing handle were out of date. Providing there were no issues with the above, the child would breathe in deeply through a filter applied to the breathing handle before exhaling at a steady rate and pressure until the test was complete. The child would have to breathe out for 10 seconds in total, although if the child had difficulty exhaling at the required speed for this duration, a shorter test of 6 seconds was accepted. An animation was used to help the children achieve the desired flow rate, consisting of blowing a cloud from one side of the screen to the other, without letting it drop off the screen or fly too high. The child performed 2 tests. Both results were documented, and the highest FE_{NO} level was used in the analysis.

- *Skin prick testing*

Skin prick testing was performed using Multi-Test PC lancets (Lincoln Diagnostics, USA). A Dipwell Tray (Lincoln Diagnostics, USA) was pre-prepared with the following allergens: cat dander; dermatophagoides pterynyssinus; grass mix; dog dander; aspergillus fumigatus; and cladosporium herbarum; as well as a positive histamine control and a negative control (Immunotek, Spain). The procedure was explained to the child and their forearm was cleaned gently with water, after ensuring the skin was free from eczema or any similar skin conditions. The Multi-Test PC lancet was inserted into the Dipwell Tray ensuring all touch-posts were coated with allergen solution. The lancet was slowly removed from the tray, and gently applied to the skin. Following one second of gentle pressure, the lancet was pressed firmly onto the skin with gentle rotation of the lancet device up and down and side to side before removal. Successful application left the imprints of the touch posts on the skin. Any excess allergen fluid on the skin was gently removed with tissue paper ensuring no cross-contamination of sites. A timer was set for 15 minutes. Children were encouraged not to scratch if the arm got itchy. After 15 minutes the arm was inspected for any wheals that developed; the raised aspects of the wheals were drawn around with pen and tape was used to lift the pen mark and stuck to a data sheet. A ruler was then used to measure the widest diameter of any of the wheals. A test was deemed positive if the wheal was greater than 3 mm, along with a positive histamine control test.

- *Spirometry*

Definitive spirometry was performed using the MasterScreen Body and PFT systems with SentrySuite measurement software version 2.17 (Vyaire Medical, Germany). ERS/ATS guidelines for obtaining suitable spirometry were used for as a guide for performing the test and test acceptability[1]. An explanation and a demonstration on how to perform the test was done before the child attempted the spirometry. Spirometry was performed with the child sat upright and wearing a nose clip. They

were instructed to take the biggest breath in possible, before blowing out as hard and as fast as they could. Children were vocally encouraged to continue breathing out until they appeared to have reached their residual volume and evidenced by no change in volume on the volume-time curve. A minimum of 3 tests were performed, aiming for the intra-test criteria as per Miller *et al*[1]. Spirometry was stopped once satisfactory testing was obtained, or if the child did not wish to continue or if the child was unable to perform adequate spirometry. The investigators were blinded to the treatment allocation at both visits. QC was performed to ensure accuracy of results. Daily volume calibrations and weekly flow calibrations were performed using a three-litre syringe. Results were measured at BTSP and Global Lung Initiative reference values were used to adjust for height, ethnicity, gender and age [2]. Spirometry was repeated at 4 separated times following completion of the exercise test: at 5-10 minutes; 15-20 minutes; 25-30 minutes; 40-45 minutes. After the final post-exercise spirometry, 400 micrograms of salbutamol (Salamol, TEVA UK Limited) was given via MDI using a Volumatic spacer (GSK, UK). Children were instructed to take 10 breaths in and out after each actuation of salbutamol, ensuring the spacer's valve clicked with each breath. Repeat spirometry was performed 15 minutes after administration of the salbutamol.

- *Cardiopulmonary exercise testing*

Cardiopulmonary exercise testing was performed on a Pediatric Cycle Ergometer (Lode, Netherlands) linked to a Masterscreen CPX system (Vyaire Medical, Germany). Children wore a fitted facemask and respiratory parameters were measured using a turbine and gas sampling tube. Data were recorded in a breath-by-breath exercise programme on JLab version 5.72 (Vyaire Medical, Germany). Heart rate was recorded using a Polar H10 heart rate sensor (Polar, UK). Oxygen saturations were monitored with a Nellcor oxygen saturation monitor (Medtronic, USA). A ramp protocol was devised to facilitate the exercise testing. This involved 1 minute of baseline measurements at rest, 3 minutes of minimally loaded cycling (7 Watts), then at an increasing rate of 1 Watt every 6 seconds (10 Watts per minute). The child was vocally encouraged to continue exercise until they could no longer consistently maintain cadence >60 rpm, with increasing encouragement as the load got higher. Perceived exertion rating was obtained every 3 minutes and at the point the child could no longer continue. 2 minutes of minimally loaded pedalling concluded the test. A test was deemed to be 'maximal' if they met 2 or more of the four criteria: Respiratory Exchange Ratio (RER) >1.00; HR ≥80% predicted (220 bpm – age); ≥9/10 on OMNI scale (pictorial scale for rating of perceived exertion[3]; peak oxygen uptake ($\dot{V}O_2$) plateau based on visual analysis. Minute ventilation, peak O_2 uptake and CO_2 production results were averaged from the last 15 seconds of peak exercise. Maximum load, heart rate and respiratory rate were the highest recorded value at the peak of exercise. Ventilatory reserve was calculated by the following equation: 1-(minute ventilation/maximal voluntary ventilation)*100, where MVV = FEV_1 x 35[4]. An automated volume calibration and gas analyser calibration were performed on each day of

testing, in line with manufacturer's instructions.

- *Withholding medication prior to testing*

Participants were asked to withhold the following medications and foods prior to testing:

- Long acting $ß_2$ agonists for 48 hours before visit
- Inhaled corticosteroids for 24 hours before visit
- Short-acting $ß_2$ agonists for 8 hours before visit (unless symptomatic)
- Leukotriene receptor antagonists for 48 hours before the visit
- Caffeine for 24 hours before the visit
- Antihistamines for 48 hours before the visit
- Consumption of food or drink (except water) in the last hour
- Consumption of foods containing nitrate/nitrites on the day of testing

eMethods 2. Inhaler intervention, Randomization, and Statistical Methods

Inhaler Intervention

A double-dummy metered dose inhaler (MDI) design was necessary to optimally double-blind the inhalers since a single inhaler design was not possible due to the presence or absence of a counter in the different metered dose inhalers. Thus, each child was randomised to either placebo/placebo, fluticasone propionate (50µg) (GSK, UK)/placebo, or fluticasone propionate (50µg)/salmeterol (25µg) (GSK, UK) inhalers, given two puffs twice daily. After extensive discussion, including with two independent experts, given the lack of evidence of effectiveness of ICS treatment[10 11], it was concluded that children on ICS treatment who had not had recent respiratory exacerbations, hospital admissions for respiratory reasons or were deemed to be ICS dependent should be washed out of their corticosteroids under supervision over 4 weeks prior to randomisation to an active arm of the study (i.e., to either ICS/placebo or ICS/LABA combination).

The children were monitored for any adverse events during the 12-week treatment period and were reassessed after this period undergoing repeat spirometry and exercise testing. The trial was overseen by an independent trial and safety monitoring committee.

Randomisation

St Mary's Pharmaceutical Unit (Cardiff, UK) independently blinded, packaged, and labelled the inhalers according to the randomisation schedule provided by the North Wales Organisation for Randomised Trials in Health (NWORTH, Bangor, UK) which also provided the clinical trial support. Children not on pre-existing corticosteroids were randomised to placebo/placebo, ICS/placebo or ICS/LABA with a 1:1:1 allocation ratio. Children on corticosteroids at the time of randomisation were washed out of their corticosteroids and were randomised to either ICS/placebo or ICS/LABA with a 1:1 allocation ratio. Since it was estimated that 86.7% of children would not be on corticosteroids at randomisation the anticipated overall allocation ratio would be 1:1.3:1.3 for placebo, ICS, ICS/LABA respectively. The randomisation was performed by dynamic allocation[20].

Masking

All participants and staff associated with the trial were blinded to treatment allocation. A procedure was in place for unblinding for any significant adverse event.

Statistical Analyses and Power Calculation

Sample size calculation

Children not on corticosteroids at the time of randomisation were randomised to placebo/placebo, ICS/placebo or ICS/LABA with a 1:1:1 allocation ratio. Children had their pre-treatment corticosteroids washed out were randomised to ICS/placebo or ICS/LABA with a 1:1 allocation ratio. The anticipated overall allocation ratio was estimated to be 1 : 1.3 : 1.3 for placebo, ICS, ICS/LABA respectively. The initial sample size calculation estimated that 144 children with %FEV_1 ≤85% with a mean %FEV_1 of 70% would be required to detect a 12% relative improvement in %FEV_1 using analysis of variance (ANOVA) at a power of 80%, %FEV_1 standard deviation of 15, and $p<0.05$. With an estimated 20% attrition, 180 children would need to be studied. The sample size calculation was revised (protocol version 3) before recruitment started, when the SD for %FEV_1 for 40 children with %FEV_1 ≤85% was noted to be 9.5. Using one-way ANOVA to show a 12% relative increase in %FEV_1, with a standard deviation for %FEV_1 of 9.5, $\alpha=0.05$ and power=80% and baseline %FEV_1 of 70% would require 67 children with complete data or 84 children after 20% attrition. During recruitment, the standard deviation of the data was re-reviewed (protocol 10), and it was suggested that an improvement of 10% absolute increase in %FEV_1 was a more clinically appropriate outcome. A revised power calculation using a conservative standard deviation for %FEV_1 of 10, α of 0.05 and power of 80%, suggested that 53 participants with completed data would be required.

Missing Data

For baseline spirometry, there were no missing pre-treatment data and 5 (9.4%) were missing post-treatment from those who withdrew. At the pre-treatment visit, two participants did not perform the exercise test due to time constraints, one did not feel well and four did not meet the criteria for

a maximal test. At the post-treatment visit three participants did not perform the exercise test due to time constraints, and four participants did not meet the criteria for a maximal test. Thus, there were 0 (0%), 7 (13.2%) and 6 (11.3%) pre-treatment and 5 (9.4%), 12 (22.6%) and 12 (22.6%) post-treatment missing data respectively for baseline, post-exercise, and post-exercise bronchodilators spirometry measures. Multiple imputation estimates equivalent to the highest percentage as recommended by White *et al* were used for missing data[21]. For ANCOVA models 10 imputations were generated and for repeated measures ANOVA 23 imputations were generated. Imputations were generated using a linear regression imputation model which included the factors and covariates to be included in the analysis model. These imputation estimates were then used in the analysis models in Stata[22] and the results pooled using Rubin's[23] rules.

Data Analysis

All data analyses were performed using Stata 15. A p-value <0.05 was considered significant. Adjustments for multiple comparisons were made using the Games Howell method[11] for analysis of covariance (ANCOVA) models. All analysis was conducted according to the principle of intention to treat.

Analysis of Covariance (ANCOVA)

ANCOVA was used in accordance with our predefined statistical analysis plan and guidelines provided by the Food and Drug Administration[24] and by the European Medicine's Agency[25] to assess the pre-/post-treatment differences. For each spirometry variable, the group allocation (ICS, ICS/LABA or placebo), relevant pre-treatment spirometry measure, gestational age, pre-treatment inhaled corticosteroid status (either weaned from pre-existing ICS treatment or not), sex (male or female), BPD (No BPD, BPD at 28 days or BPD at 36 weeks' gestation) and intrauterine growth restriction (IUGR or no IUGR) were added to the analysis model. Sensitivity analysis was performed for children who were not previously on ICS treatment. Assumptions of ANCOVA were tested prior to final model fitting. The group factor is of most interest in this case, therefore only interactions of pre-treatment result and gestational age with group were included where required. Scatterplots of

covariates with the dependent variable were produced, split by group. If the regression line was similar in all groups, then no interaction was included in the analysis model but if the regression line was different for the different groups, then an interaction was included.

Repeated Measures Analysis of Variance

Repeated measures analysis of variance was performed to assess change in spirometry measures from baseline to post-exercise, and from post-exercise to post-exercise bronchodilator spirometry, across the three treatment groups at pre-treatment and post-treatment visits.

eTable 1: Randomized groups

Inhaled corticosteroid status	ICS	ICS/LABA	Placebo	Total
Not currently taking corticosteroids	15	15	14	44
Washed-out from corticosteroids	5	4	0	9
**Group Total**	**20**	**19**	**14**	**53**

eTable 2: Comparisons between attenders and nonattenders

	Nonattenders	Attenders
	53	53
**Male**	28 (53%)	24 (45%)
**Age (y)**	10.1 (1.3)*	10.8 (1.3)
**Gestational Age (wks)**	30.5 (2.6)	29.7 (2.9)
**Birthweight (g)**	1535 (502)	1392 (567)
**Birthweight (z-score)**	-0.04 (1.4)	-0.22 (1.38)
**Height (cm)**	140.4 (8.6)	143.4 (11.24)
**Height (z-score)**	0.29 (1.2)	0.05 (1.2)
**Weight (kg)**	34.1 (9.3)	37.2 (11.0)
**Weight (z-score)**	0.07 (1.3)	0.028 (1.4)
**BMI**	17.1 (3.2)	17.7 (3.3)
**BMI (z-score)**	-0.15 (1.4)	-0.16 (1.4)

eTable 3: Reasons for withdrawal

Reason for withdrawal	ICS	ICS/LABA	Placebo	Total
The mother worried about her daughter taking inhalers as may have negative effects on her daughter	1	0	0	1
Child developed a cough within 24 hours of beginning trial medication so stopped and parents decided not to restart trial medication.	1	0	0	1
Two children did not wish to take trial medication despite parental encouragement	0	1	1	2
Poor compliance by child and difficulty in contacting the parents for follow up visit.	0	1	0	1

eTable 4: Results of ANCOVA of spirometry measures

		Coefficient	SE	t	P	95% CI	
						Lower	Upper
**Percentage Predicted FEV$_1$**	Group (placebo vs ICS)	5.98	43.44	0.14	0.89	-82.10	94.07
	Group (placebo vs ICS/LABA)	159.02	48.78	3.26	**0.002**	60.13	257.91
	Corticosteroids (Not weaned vs weaned)	3.79	3.88	0.98	0.34	-4.07	11.66
	Sex (Female vs Male)	1.62	3.12	0.52	0.61	-4.72	7.96
	BPD (None vs BPD28 days)	2.04	5.62	0.36	0.72	-9.41	13.49
	BPD (None vs BPD36 weeks)	4.10	4.51	0.91	0.37	-5.07	13.27
	IUGR (No IUGR vs IUGR)	-1.76	3.90	-0.45	0.65	-9.74	6.21
	Pre-treatment value	0.61	0.26	2.37	**0.02**	0.09	1.14
	Gestation	0.22	0.15	1.46	0.15	-0.09	0.54
	Group (ICS vs placebo) * Pre value	-0.02	0.39	-0.05	0.96	-0.81	0.77
	Group (ICS/LABA vs placebo) * Pre value	-0.97	0.40	-2.40	**0.02**	-1.78	-0.15
	Group (ICS vs placebo) * gestation	0.02	0.18	0.09	0.93	-0.35	0.38
	Group (ICS/LABA vs placebo) * gestation	-0.34	0.18	-1.89	0.07	-0.71	0.02
**Percentage Predicted FEF$_{25-75\%}$**	Group (placebo vs ICS)	-10.83	46.75	-0.23	0.82	-105.62	83.96
	Group (placebo vs ICS/LABA)	112.38	48.83	2.30	**0.03**	13.39	211.37
	Corticosteroids (Not weaned vs weaned)	4.75	5.12	0.93	0.36	-5.64	15.13
	Sex (Female vs Male)	2.12	4.05	0.52	0.60	-6.10	10.34
	BPD (None vs BPD28 days)	-7.36	7.05	-1.04	0.30	-21.68	6.96
	BPD (None vs BPD36 weeks)	-0.36	5.97	-0.06	0.95	-12.47	11.75
	IUGR (No IUGR vs IUGR)	-7.15	5.17	-1.38	0.18	-17.70	3.40
	Pre-treatment value	0.89	0.17	5.18	**< 0.001**	0.54	1.24
	Gestation	0.06	0.19	0.29	0.78	-0.34	0.45
	Group (placebo vs ICS) * Pre value	-0.29	0.29	-1.00	0.32	-0.87	0.30
	Group (placebo vs ICS/LABA) * Pre value	-0.23	0.24	-0.95	0.35	-0.71	0.26
	Group (placebo vs ICS) * gestation	0.16	0.23	0.69	0.50	-0.31	0.62
	Group (placebo vs ICS/LABA) * gestation	-0.39	0.23	-1.69	0.10	-0.85	0.08
**Percentage Predicted FVC**	Group (placebo vs ICS)	15.81	32.00	0.49	0.62	-48.99	80.61
	Group (placebo vs ICS/LABA)	33.57	32.79	1.02	0.31	-32.79	99.92
	Corticosteroids (Not weaned vs weaned)	0.20	3.50	0.06	0.96	-6.89	7.28
	Sex (Female vs Male)	3.53	2.69	1.31	0.20	-1.92	8.98
	BPD (None vs BPD28 days)	-0.07	4.76	-0.01	0.99	-9.73	9.59
	BPD (None vs BPD36 weeks)	1.53	3.92	0.39	0.70	-6.41	9.48
	IUGR (No IUGR vs IUGR)	1.86	3.44	0.54	0.59	-5.14	8.86
	Pre-treatment value	1.10	0.15	7.55	**< 0.001**	0.80	1.40
	Gestation	0.18	0.13	1.36	0.18	-0.09	0.44
	Group (ICS vs placebo) * gestation	-0.07	0.15	-0.47	0.64	-0.39	0.24
	Group (ICS/LABA vs placebo) * gestation	-0.16	0.15	-1.03	0.31	-0.47	0.15
**FEV$_1$ / FVC ratio**	Group (placebo vs ICS)	0.10	0.24	0.43	0.67	-0.38	0.58
	Group (placebo vs ICS/LABA)	0.52	0.24	2.12	**0.04**	0.02	1.01
	Corticosteroids (Not weaned vs weaned)	0.02	0.02	0.84	0.41	-0.03	0.06
	Sex (Female vs Male)	0.02	0.02	1.16	0.26	-0.02	0.06
	BPD (None vs BPD28 days)	-0.04	0.03	-1.22	0.23	-0.10	0.03
	BPD (None vs BPD36 weeks)	0.01	0.03	0.28	0.78	-0.05	0.06
	IUGR (No IUGR vs IUGR)	-0.02	0.02	-0.67	0.51	-0.06	0.03
	Pre-treatment value	1.08	0.15	7.18	**< 0.001**	0.77	1.38
	Gestation	0.00	0.00	-0.58	0.57	0.00	0.00
	Group (placebo vs ICS) * Pre value	-0.51	0.20	-2.49	**0.02**	-0.92	-0.09
	Group (placebo vs ICS/LABA) * Pre value	-0.51	0.20	-2.55	**0.02**	-0.92	-0.10
	Group (placebo vs ICS) * gestation	0.00	0.00	1.39	0.17	0.00	0.00
	Group (placebo vs ICS/LABA) * gestation	0.00	0.00	-0.27	0.79	0.00	0.00
**Percentage Predicted PEFR**	Group (placebo vs ICS)	-19.93	55.06	-0.36	0.72	-131.86	91.99
	Group (placebo vs ICS/LABA)	126.61	51.48	2.46	**0.02**	22.08	231.14
	Corticosteroids (Not weaned vs weaned)	5.84	5.46	1.07	0.29	-5.28	16.97
	Sex (Female vs Male)	3.64	4.16	0.88	0.39	-4.80	12.09
	BPD (None vs BPD28 days)	-4.36	7.18	-0.61	0.55	-8.95	10.24
	BPD (None vs BPD36 weeks)	-1.91	5.94	-0.32	0.75	-13.96	10.13
	IUGR (No IUGR vs IUGR)	-5.22	5.26	-0.99	0.33	-15.93	5.49
	Pre-treatment value	0.43	0.16	2.67	**0.01**	0.10	0.76
	Gestation	0.14	0.20	0.70	0.49	-0.26	0.54
	Group (placebo vs ICS) * gestation	0.15	0.27	0.55	0.59	-0.39	0.69
	Group (placebo vs ICS/LABA) * gestation	-0.50	0.24	-2.08	**0.05**	-0.99	-0.01

eTable 5: Sensitivity analyses showing ANCOVA results of spirometry measures of children who were corticosteroid naive at randomisation

		Coefficient	SE	t	P	95% CI	
						Lower	Upper
**Percentage Predicted FEV_1**	Group (placebo vs ICS)	4.85	48.70	0.10	0.92	-94.81	104.52
	Group (placebo vs ICS/LABA)	155.54	52.07	2.99	**0.006**	48.89	262.19
	Sex (Female vs Male)	3.48	3.69	0.94	0.35	-4.11	11.08
	BPD (None vs BPD28 days)	3.73	6.59	0.57	0.58	-9.87	17.33
	BPD (None vs BPD36 weeks)	5.46	5.32	1.03	0.31	-5.49	16.41
	IUGR (No IUGR vs IUGR)	-2.09	4.67	-0.45	0.66	-11.69	7.52
	Pre value	0.59	0.27	2.16	**0.04**	0.03	1.14
	Gestation	0.24	0.17	1.45	0.16	-0.10	0.59
	Group (ICS vs placebo) * Pre value	-0.01	0.42	-0.02	0.99	-0.86	0.85
	Group (ICS/LABA vs placebo) * Pre value	-0.91	0.43	-2.09	**0.05**	-1.79	-0.02
	Group (ICS vs placebo) * gestation	0.01	0.21	0.06	0.95	-0.41	0.44
	Group (ICS/LABA vs placebo) * gestation	-0.34	0.19	-1.75	0.09	-0.73	0.06
**Percentage Predicted $FEF_{25-75\%}$**	Group (placebo vs ICS)	-17.40	49.97	-0.35	0.73	-119.76	84.95
	Group (placebo vs ICS/LABA)	130.78	48.48	2.70	**0.01**	31.61	229.95
	Sex (Female vs Male)	4.69	4.53	1.03	0.31	-4.65	14.03
	BPD (None vs BPD28 days)	-2.67	7.89	-0.34	0.74	-18.90	13.56
	BPD (None vs BPD36 weeks)	1.58	6.59	0.24	0.81	-11.96	15.13
	IUGR (No IUGR vs IUGR)	-8.72	5.72	-1.52	0.14	-20.49	3.06
	Pre value	0.86	0.17	5.06	**< 0.001**	0.51	1.20
	Gestation	0.11	0.20	0.56	0.58	-0.30	0.52
	Group (placebo vs ICS) * Pre value	-0.30	0.29	-1.03	0.31	-0.90	0.30
	Group (placebo vs ICS/LABA) * Pre value	-0.32	0.24	-1.34	0.19	-0.81	0.17
	Group (placebo vs ICS) * gestation	0.19	0.25	0.73	0.47	-0.33	0.71
	Group (placebo vs ICS/LABA) * gestation	-0.43	0.23	-1.88	0.07	-0.89	0.04
**Percentage Predicted FVC**	Group (placebo vs ICS)	22.15	38.54	0.57	0.57	-56.55	100.85
	Group (placebo vs ICS/LABA)	36.52	35.97	1.02	0.32	-36.84	109.89
	Sex (Female vs Male)	4.61	3.44	1.34	0.19	-2.45	11.67
	BPD (None vs BPD28 days)	0.88	5.82	0.15	0.88	-11.06	12.82
	BPD (None vs BPD36 weeks)	1.89	4.87	0.39	0.70	-8.11	11.89
	IUGR (No IUGR vs IUGR)	2.28	4.28	0.53	0.60	-6.50	11.05
	Pre value	1.17	0.19	6.21	**< 0.001**	0.78	1.55
	Gestation	0.18	0.15	1.23	0.23	-0.12	0.48
	Group (ICS vs placebo) * gestation	-0.10	0.19	-0.55	0.59	-0.48	0.28
	Group (ICS/LABA vs placebo) * gestation	-0.17	0.17	-1.00	0.33	-0.51	0.18
**FEV_1 / FVC ratio**	Group (placebo vs ICS)	0.02	0.25	0.07	0.95	-0.49	0.53
	Group (placebo vs ICS/LABA)	0.62	0.25	2.47	**0.02**	0.11	1.14
	Sex (Female vs Male)	0.03	0.02	1.33	0.20	-0.02	0.07
	BPD (None vs BPD28 days)	-0.03	0.04	-0.70	0.49	-0.10	0.05
	BPD (None vs BPD36 weeks)	0.01	0.03	0.35	0.73	-0.05	0.07
	IUGR (No IUGR vs IUGR)	-0.02	0.03	-0.60	0.55	-0.07	0.04
	Pre value	1.05	0.15	6.98	**< 0.001**	0.74	1.36
	Gestation	0.00	0.00	-0.40	0.69	0.00	0.00
	Group (placebo vs ICS) * Pre value	-0.46	0.21	-2.17	**0.04**	-0.90	-0.02
	Group (placebo vs ICS/LABA) * Pre value	-0.57	0.21	-2.77	**0.01**	-0.99	-0.15
	Group (placebo vs ICS) * gestation	0.00	0.00	1.47	0.15	0.00	0.00
	Group (placebo vs ICS/LABA) * gestation	0.00	0.00	-0.45	0.66	0.00	0.00
**Percentage Predicted PEFR**	Group (placebo vs ICS)	-55.49	66.41	-0.84	0.41	-192.88	81.89
	Group (placebo vs ICS/LABA)	121.75	50.35	2.42	**0.02**	18.58	224.92
	Sex (Female vs Male)	2.10	4.68	0.45	0.66	-7.54	11.74
	BPD (None vs BPD28 days)	-1.21	7.97	-0.15	0.88	-17.61	15.20
	BPD (None vs BPD36 weeks)	-0.01	6.87	0.00	1.00	-14.21	14.19
	IUGR (No IUGR vs IUGR)	-1.42	6.42	-0.22	0.83	-14.79	11.95
	Pre value	0.51	0.17	3.01	**0.006**	0.16	0.86
	Gestation	0.18	0.20	0.89	0.38	-0.24	0.60
	Group (placebo vs ICS) * gestation	0.33	0.32	1.01	0.33	-0.35	1.00
	Group (placebo vs ICS/LABA) * gestation	-0.49	0.23	-2.11	**0.04**	-0.97	-0.01

eTable 6: Spirometry at baseline, postexercise and after postexercise bronchodilator

	Group	Visit	Baseline	Post-exercise	Post exercise-BD
$\%FEV_1$ (SD)	ICS	Pre	75.1 (8.0)	73.1 (10.2)	84.5 (10.5)
		Post	81.1 (12.3)	82.0 (14.1)	86.8 (14.6)
	ICS/LABA	Pre	77.9 (7.9)	78.1 (4.5)	84.9 (5.2)
		Post	86.2 (6.4)	86.0 (5.1)	88.4 (4.9)
	Placebo	Pre	72.4 (11.2)	72.9 (11.2)	82.4 (9.9)
		Post	71.2 (12.1)	69.5 (12.1)	79.4 (9.1)
$\%FEF_{25\text{-}75\%}$ (SD)	ICS	Pre	48.0 (15.2)	N/A	68.0 (16.1)
		Post	57.6 (15.1)		73.6 (19.9)
	ICS/LABA	Pre	54.6 (19.4)		73.9 (17.9)
		Post	70.8 (17.0)		79.1 (18.8)
	Placebo	Pre	48.1 (21.7)		64.7 (17.3)
		Post	48.2 (23.4)		69.3 (27.7)
%FVC (SD)	ICS	Pre	91.0 (10.5)	86.2 (10.9)	90.1 (13.7)
		Post	91.9 (14.3)	90.0 (14.7)	90.0 (15.7)
	ICS/LABA	Pre	91.8 (8.3)	88.7 (9.6)	89.1 (10.8)
		Post	91.8 (11.0)	90.6 (10.1)	91.2 (10.9)
	Placebo	Pre	90.0 (7.3)	85.5 (6.1)	90.2 (14.7)
		Post	88.8 (12.2)	83.4 (8.6)	85.4 (11.1)
FEV_1/FVC ratio (SD)	ICS	Pre	0.73 (0.10)	0.75 (0.11)	0.83 (0.07)
		Post	0.78 (0.08)	0.80 (0.07)	0.85 (0.06)
	ICS/LABA	Pre	0.75 (0.11)	0.77 (0.10)	0.84 (0.08)
		Post	0.83 (0.08)	0.84 (0.08)	0.85 (0.08)
	Placebo	Pre	0.71 (0.12)	0.75 (0.13)	0.81 (0.09)
		Post	0.71 (0.14)	0.74 (0.15)	0.82 (0.11)

eFigure: (a) %FVC and (b) FEV$_1$/FVC ratio at baseline, after exercise, and after postexercise bronchodilator

(a)

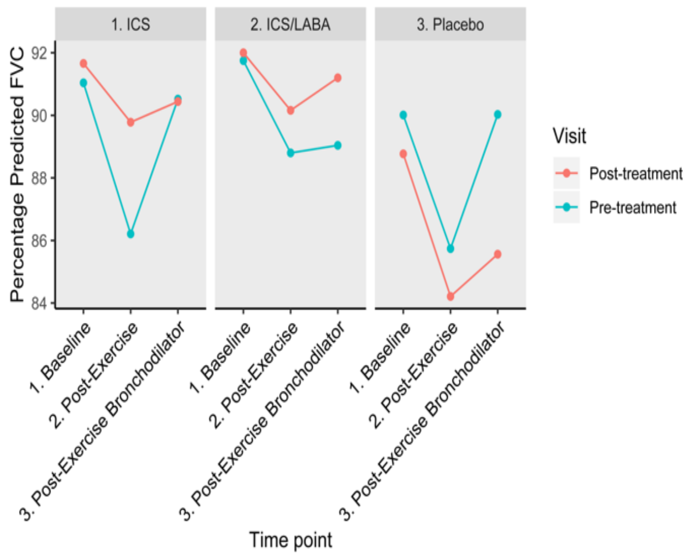

(b)

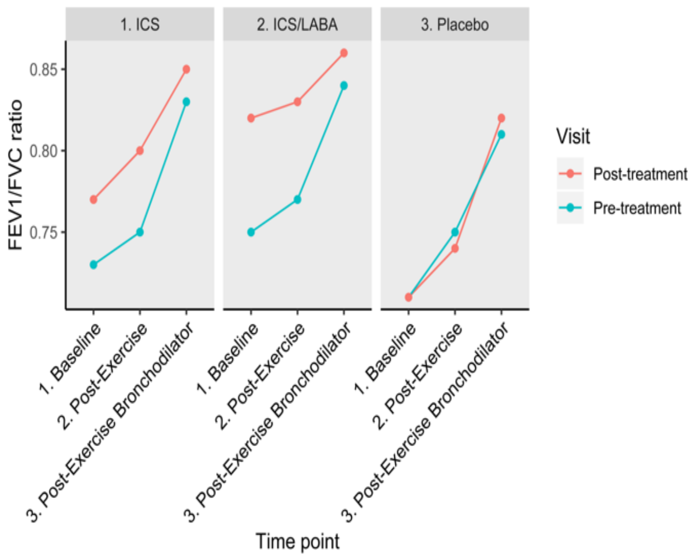

	**Pre-treatment**		**Post-treatment**		**Pre-treatment vs Post-treatment**		
	Baseline vs Post-exercise	Post-exercise vs Post BD	Baseline vs Post-exercise	Post-exercise vs Post BD	Baseline vs baseline	Post-exercise vs post-exercise	Post BD vs Post BD
	**Percentage Predicted FVC**						
ICS	**0.05**	0.13	0.50	0.81	0.79	0.18	0.97
ICS/LABA	0.18	0.92	0.47	0.70	0.91	0.59	0.37
Placebo	0.12	0.13	0.18	0.71	0.64	0.65	0.15

	**Pre-treatment**		**Post-treatment**		**Pre-treatment vs Post-treatment**		
	Baseline vs Post-exercise	Post-exercise vs Post BD	Baseline vs Post-exercise	Post-exercise vs Post BD	Baseline vs baseline	Post-exercise vs post-exercise	Post BD vs Post BD
	**Percentage Predicted FEV$_1$/FVC**						
ICS	0.34	**0.001**	0.31	**0.03**	**0.03**	**0.03**	0.34
ICS/LABA	0.12	**0.002**	0.77	0.27	**<0.001**	**0.01**	0.32
Placebo	0.07	**0.02**	0.26	**0.02**	0.81	0.85	0.68

The figures show the %FVC and FEV$_1$/FVC at baseline, after exercise and after post-exercise bronchodilator, and the tables show the associated p-values from comparisons of means of the %FVC and FEV$_1$/FVC from repeated measures ANOVA. Abbreviation: Post-Ex BD – post-exercise bronchodilator.

eReferences

1. Miller MR, Hankinson J, Brusasco V, et al. Standardisation of spirometry. *Eur Respir J.* 2005;26(2):319-338.
2. Quanjer PH, Stanojevic S, Cole TJ, et al. Multi-ethnic reference values for spirometry for the 3-95-yr age range: the global lung function 2012 equations. *Eur Respir J.* 2012;40(6):1324-1343.
3. Barkley JE, Roemmich JN. Validity of the CALER and OMNI-bike ratings of perceived exertion. *Med Sci Sports Exerc.* 2008;40(4):760-766.
4. Joshi S, Powell T, Watkins WJ, Drayton M, Williams EM, Kotecha S. Exercise-induced bronchoconstriction in school-aged children who had chronic lung disease in infancy. *J Pediatr.* 2013;162(4):813-818 e811.
