# Supplementary material for: Inhaled Corticosteroids Alone and in Combination With Long-Acting β2 Receptor Agonists to Treat Reduced Lung Function in Preterm-Born Children: A Randomized Clinical Trial
Source: JAMA Pediatr. 2021 Dec 13;176(2):1–9. doi: 10.1001/jamapediatrics.2021.5111 (PMC8669602; doi:10.1001/jamapediatrics.2021.5111)
Supplement: Supplement 3. — Data Sharing Statement [file jamapediatr-e215111-s003.pdf]

Data Sharing Statement

Data
Data available: Yes
Data types: Other (please specify)
Additional Information: Data wil be available after two years of publication due to data sharing agreements in place.
How to access data: Please contact corresponding author.
When available: beginning date: 08-01-2023, end date: 07-31-2025

Supporting Documents
Document types: None

Additional Information
Who can access the data: Anyone requesting the data.
Types of analyses: For specific question to be addressed.
Mechanisms of data availability: After signed data access agreement.
Any additional restrictions: Only anonymised data can be made available.
